# Synthesis of Chromium(II) Complexes with Chelating Bis(alkoxide) Ligand and Their Reactions with Organoazides and Diazoalkanes

**DOI:** 10.3390/molecules25020273

**Published:** 2020-01-09

**Authors:** Sudheer S. Kurup, Richard J. Staples, Richard L. Lord, Stanislav Groysman

**Affiliations:** 1 Department of Chemistry, Wayne State University, 5101 Cass Ave., Detroit, MI 48202, USA; sudheer.kurup@wayne.edu; 2 Department of Chemistry, Michigan State University, 578 S Shaw Ln, East Lansing, MI 48824, USA; staples@chemistry.msu.edu; 3 Department of Chemistry, Grand Valley State University, 1 Campus Dr, Allendale, MI 49401, USA

**Keywords:** alkoxides, azides, carbodiimides

## Abstract

Synthesis of new chromium(II) complexes with chelating bis(alkoxide) ligand [OO]^Ph^ (H_2_[OO]^Ph^ = [1,1′:4′,1′’-terphenyl]-2,2′’-diylbis(diphenylmethanol)) and their subsequent reactivity in the context of catalytic production of carbodiimides from azides and isocyanides are described. Two different Cr(II) complexes are obtained, as a function of the crystallization solvent: mononuclear Cr[OO]^Ph^(THF)_2_ (in toluene/THF, THF = tetrahydrofuran) and dinuclear Cr_2_([OO]^Ph^)_2_ (in CH_2_Cl_2_/THF). The electronic structure and bonding in Cr[OO]^Ph^(THF)_2_ were probed by density functional theory calculations. Isolated Cr_2_([OO]^Ph^)_2_ undergoes facile reaction with 4-MeC_6_H_4_N_3_, 4-MeOC_6_H_4_N_3_, or 3,5-Me_2_C_6_H_3_N_3_ to yield diamagnetic Cr(VI) bis(imido) complexes; a structure of Cr[OO]^Ph^(N(4-MeC_6_H_4_))_2_ was confirmed by X-ray crystallography. The reaction of Cr_2_([OO]^Ph^)_2_ with bulkier azides N_3_R (MesN_3_, AdN_3_) forms paramagnetic products, formulated as Cr[OO]^Ph^(NR). The attempted formation of a Cr–alkylidene complex (using N_2_CPh_2_) instead forms chromium(VI) bis(diphenylmethylenehydrazido) complex Cr[OO]^Ph^(NNCPh_2_)_2_. Catalytic formation of carbodiimides was investigated for the azide/isocyanide mixtures containing various aryl azides and isocyanides. The formation of carbodiimides was found to depend on the nature of organoazide: whereas bulky mesitylazide led to the formation of carbodiimides with all isocyanides, no carbodiimide formation was observed for 3,5-dimethylphenylazide or 4-methylphenylazide. Treatment of Cr_2_([OO]^Ph^)_2_ or H_2_[OO]^Ph^ with NO^+^ leads to the formation of [1,2-b]-dihydroindenofluorene, likely obtained via carbocation-mediated cyclization of the ligand.

## 1. Introduction

Organoazides constitute efficient and sustainable precursors for the formation of reactive nitrene functionality, which engenders many useful transformations, including C–H bond amination, aziridination, formation of azoarenes and carbodiimides, and others [1,2,3,4,5,6,7]. Transition metals generally enable milder conditions for the formation of nitrenes and higher selectivity in their subsequent transfer. The majority of nitrene-transfer studies are focused on middle and late transition metals, which generally feature weaker metal–imido bonds and therefore exhibit more reactive nitrene functionalities [8,9,10,11,12,13,14,15,16,17,18,19,20,21]. In contrast, early transition metals (particularly chromium) typically exhibit less reactive nitrene functionalities due to the stability of multiple metal-nitrogen bonds [22,23,24,25,26,27,28]. However, recent studies demonstrated the ability of coordinatively unsaturated chromium(IV)–imido to transfer nitrene to olefins or isocyanides to form aziridines and carbodiimides, respectively [29,30,31]. C–C and C–H bond activation reactions featuring reactive chromium(IV)–imido were also reported [32]. As part of our ongoing investigation of nitrene- and carbene-transfer chemistry mediated by 3d metal complexes in bulky alkoxide ligand environments [33,34], we have reported the synthesis and reactions of chromium–imido complexes featuring two bulky monodentate alkoxides [OC*^t^*Bu_2_Ph] [31]. We have demonstrated that Cr^IV^(OC*^t^*Bu_2_Ph)_2_(NR’) complexes, which were formed for bulky R’ groups (R’ = Mes, 2,6-Et_2_C_6_H_3_, 1-adamantyl), enabled catalytic transfer of the nitrene group [NR’] to isocyanides to form carbodiimides. In contrast, the reaction of less bulky *para*-substituted aryl azides N_3_R’ (R’ = 4-MeC_6_H_4_, 4-MeOC_6_H_4_, 4-CF_3_C_6_H_4_) formed Cr(VI) complexes Cr^VI^(OR)_2_(NR’)_2_ that were catalytically inactive. Recently, we have reported a new chelating bis(alkoxide) ligand [OO]^Ph^ [35] that created a similar bis(alkoxide) ligand environment but warranted a higher degree of stability of the resulting iron complex Fe[OO]^Ph^, and consequently led to a broader range of catalytic nitrene transfer compared with related Fe(OR)_2_ systems [36,37]. Herein, we report our synthetic, structural, and catalytic studies on the chemistry of Cr^II^[OO]^Ph^ with organoazides and diazoalkanes and compare this reactivity with the previously reported Cr^II^(OC*^t^*Bu_2_Ph)_2_ system.

## 2. Results and Discussion

### 2.1. Synthesis and Structures of Cr(II) Precursors

**Scheme 1:**
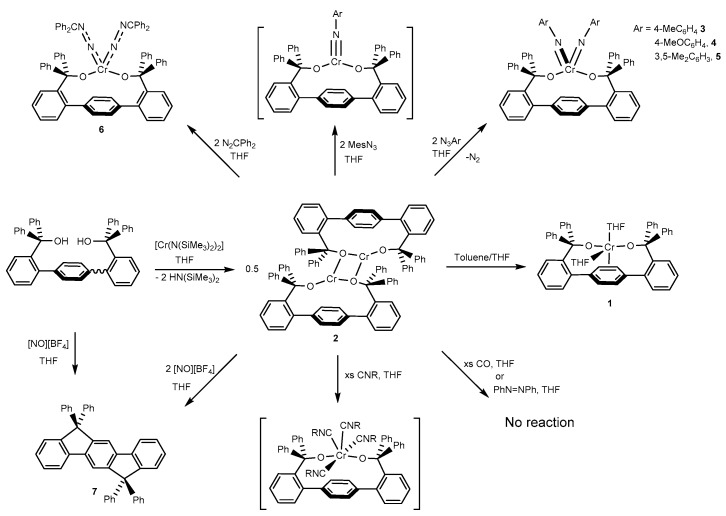
Synthesis and reactivity of Cr(II) complexes in the chelating bis(alkoxide) ligand environment.

Treatment of the chromium(II) amide precursor with one equivalent of H_2_[OO]^Ph^ in THF produces a green solid. Recrystallization of the product from the toluene/THF mixture yields mononuclear pale green Cr[OO]^Ph^(THF)_2_ (**1**) related to the mononuclear Cr(OC*^t^*Bu_2_(3,5-Ph_2_C_6_H_3_))_2_(THF)_2_ [38]. In contrast, recrystallization of the product from a mixture of two polar solvents (CH_2_Cl_2_ and THF) forms pale green–blue homodinuclear complex Cr_2_[OO]^Ph^_2_ (**2**, Scheme 1) isolated in 79% yield. Homodinuclear complex **2** is structurally related to the previously reported alkoxide-bridged Cr_2_(OC*^t^*Bu_2_Ph)_4_ [31], Cr_2_(OC^t^Bu_3_)_2_(μ^2^-OC*^t^*Bu_2_H)_2_ [39], and siloxide-bridged Cr_2_(OSi*^t^*Bu_3_)_4_ [40]. We note that monodentate alkoxides [OC*^t^*Bu_2_Ph] and [OC*^t^*Bu_2_(3,5-Ph_2_C_6_H_3_)] formed dinuclear or mononuclear complexes selectively [31,38], whereas the chelating ligand appears to support both types of geometries in the solid state, depending on the nature of the crystallization medium.

**Figure 1:**
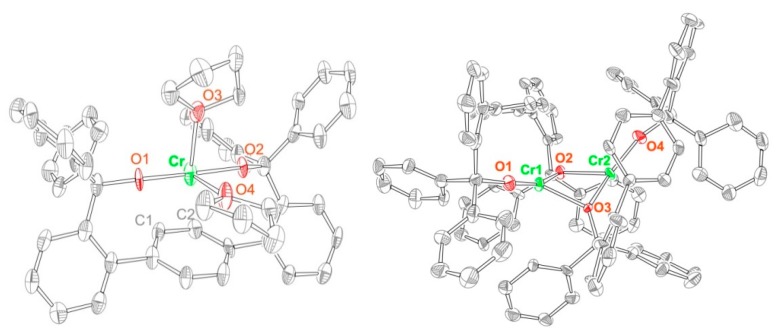
X-ray structures of **1** (left) and **2** (right), 50% probability ellipsoids. H atoms and co-crystallized solvent molecules were omitted for clarity. Selected bond distances (Å) and angles (°) for **1**: Cr O1, 1.924(6), Cr O2, 1.935(6), Cr O3, 2.156(7), Cr O4, 2.277(6), Cr --- Ph 2.49(1), O1 Cr1 O2, 173.6(2), O1 Cr1 O3, 86.7(2), O2 Cr1 O3, 90.0(3), O1 Cr1 O4, 85.9(2), O2 Cr1 O4, 88.5(2), O3 Cr1 O4, 88.1(2). Selected bond distances (Å) and angles (°) for **2**: Cr1 O1, 1.788(5), Cr2 O4, 1.799(5), Cr1 O2, 1.976(5), Cr1 O3, 2.013(6), Cr2 O3, 1.981(5), Cr2 O2, 2.049(5), Cr1 --- Cr2, 2.896(2), O2 Cr1 O3, 81.2(2), O1 Cr1 O2, 150.2(3), O1 Cr1 O3, 120.1(2).

**Figure 2:**
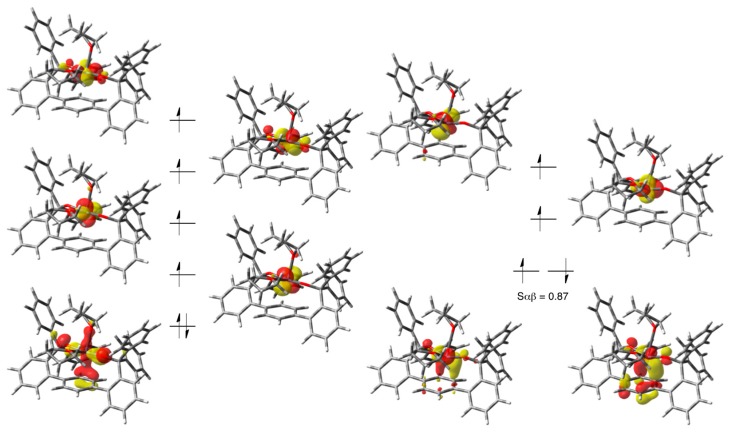
Corresponding orbital diagrams for **1_Q_** (left) and **1_T_** (right) at the BP86-D3/def2-SVP level of theory. Orbitals are plotted with an isosurface value of 0.05 au.

The structures of complexes **1** and **2** are given in Figure 1. The structure of **1** is similar to the previously reported structure of Fe[OO]^Ph^(THF)_2_ [35]. However, the structure of Fe[OO]^Ph^(THF)_2_ exhibited a very long distance between the metal and the central phenyl (>3 Å), indicating no interaction [35]. In contrast, the distance between the chromium center and the central phenyl in **1** is 2.49(1) Å, which suggests bonding between the metal and the phenyl ring. The inter-alkoxide RO-Cr-OR and THF-Cr-THF angles are 173.6(2)° and 88.1(2)°, respectively. Two independent molecules of homodinuclear complex **2** were found in the asymmetric unit exhibiting similar structural parameters; only one is shown. Each [OO]^Ph^ ligand provides one terminal and one bridging alkoxide donor; no coordination to the central phenyl is observed in this case (Cr---Ph ≥ 3.0 Å). While the overall geometry and metrics in **2** are similar to those of Cr_2_(OC*^t^*Bu_2_Ph)_4_ and related complexes [32,39,40], the metal centers in **2** exhibit significant distortion of trigonal planar geometry, as indicated by the considerable difference in O-Cr-O angles (Figure 2) and displacement of Cr from the trigonal plane (varies between 0.105 and 0.348 Å).

To better understand the electronic structure and bonding in **1**, density functional theory (DFT) calculations were performed. Singlet (**1_S_**), triplet (**1_T_**), and quintet (**1_Q_**) states were optimized. **1_Q_** is lowest in free energy, followed by **1_T_** at 19.7 kcal/mol, and **1_S_** at 28.6 kcal/mol. The optimized structure of **1_Q_** agrees well with the crystallographically determined structure and has bond lengths of Cr–O1 = 1.915 Å, Cr–O2 = 1.928 Å, Cr–O3 = 2.203 Å, Cr–O4 = 2.281 Å, Cr–C1 = 2.475 Å, and Cr–C2 = 2.525 Å; both **1_T_** and **1_S_** are inconsistent with the x-ray data (Table S1, SI). Based on the spin density localized at Cr, **1_Q_** is best described as a high-spin Cr(II) species. Similar to what was observed in the optimization of different spin states of Fe[OO]^Ph^(THF)_2_ [35], the higher-energy, lower spin states **1_T_**/**1_S_** exhibit much shorter Cr–phenyl contacts of 2.1–2.2 Å. Mayer bond orders for Cr–C1/C2 are calculated to be 0.25 and 0.23 in **1_Q_**. In comparison, quintet Fe[OO]^Ph^(THF)_2_ (with Fe–C distances of 2.8–2.9 Å) has smaller bond orders of 0.06 and 0.09, whereas **1_T_** has larger bond orders of 0.64 and 0.65. For perspective, M–O_THF_ bond orders are 0.2–0.3 and the M–O_alkoxide_ bond orders are 0.6–0.9. Frontier orbital diagrams of **1_Q_** and **1_T_** (Figure 2) illustrate the Cr–phenyl bonding in both spin states. The much stronger bonding in **1_T_** is due to the *d*-π backbonding that is possible with intermediate-spin Cr(II) ion. This back-bonding is not significant in **1_Q_** due to half-occupation of that *d*-orbital in the high-spin Cr(II) ion. Collectively, these computational data suggest a weak covalent interaction between Cr and the bridging phenyl moiety in **1**.

We have also investigated the structures of **1** and **2** in solution. Both compounds give rise to paramagnetic NMR spectra, as anticipated. The UV–Vis spectra of **1** and **2** (collected in THF) are very similar, exhibiting a peak at approximately 710 nm, and a shoulder at approximately 510 nm (Figures S39 and S40, SI). The solution magnetic moment of **1** (3.0(3) μB, C_6_D_6_) was found to be significantly lower than the expected spin-only value of 4.9 μB. Recalculated for the structure of Cr_2_([OO]^Ph^)_2_, the observed magnetic moment is 2.6(2) μB (per Cr), which is close to the observed value of 2.5(3) μB reported for Cr_2_(OC*^t^*Bu_2_Ph)_2_ [31]. These data suggest that **1** undergoes dimerization in solution to form **2**.

### 2.2. Reactions of Chromium(II) Complexes with Organoazides, Azoarenes and Diazoalkanes

**Figure 3:**
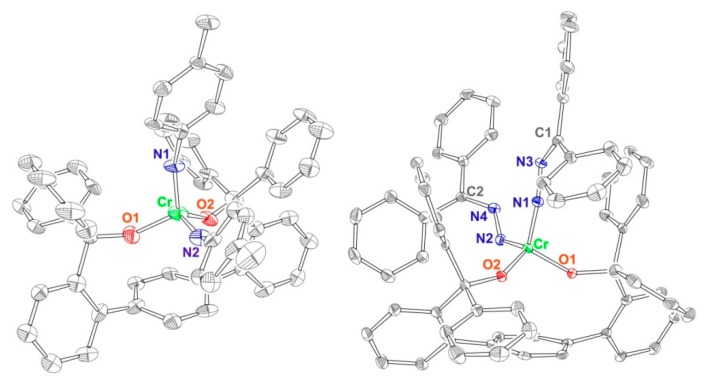
X-ray structure of **3** (left) and **6** (right), 50% probability ellipsoids. H atoms and co-crystallized solvent molecules were omitted for clarity. Selected bond distances (Å) for **6**: Cr O1 1.807(2), Cr O2 1.777(2), Cr N1 1.645(3), Cr N2 1.716(3), N1 N3 1.305(4), N2 N4 1.301(4). Selected bond angles (°): O2 Cr O1 110.0(1), N1 Cr N3 99.7(2), N1 Cr O2 110.5(1), N2 Cr O2 109.2(1), N1 Cr O1 113.1(1), Cr N1 N3 159.8(2), Cr N2 N4 131.2(2).

Following the synthesis of the chromium(II) precursor, its reactivity with organoazides was investigated. Complex **2** was utilized as a precursor in group-transfer reactions. Similar to Cr_2_(OC*^t^*Bu_2_Ph)_4_ [31], **2** was found to exhibit profoundly distinct reactivity with non-bulky (*4*-tolyl, *4*-methoxyphenyl, and 3,5-dimethylphenyl) vs. bulky (mesityl, 1-adamantyl) organoazides. Treating a green solution of **2** with two equivalents of *para*-tolylazide in THF leads to a dark-brown solution, from which diamagnetic **3** is isolated in 69% yield by recrystallization from THF/ether. Similarly, treating **2** with two equivalents of 4-MeOC_6_H_4_N_3_ or 3,5-Me_2_C_6_H_3_N_3_ forms brown solutions containing diamagnetic products **4** and **5** in 54% and 59%, respectively. We were not able to obtain X-ray quality crystals of **4** and **5**, however, their NMR and UV–Vis data (see SI) suggests similarity to **3**. The overall connectivity of **3** was confirmed by X-ray structure determination (Figure 3). The structure reveals a distorted tetrahedral geometry, consistent with Cr(VI) bis(imido) formulation. No chromium-phenyl interaction is observed in this case, with the chromium-phenyl distance exceeding 3.5 Å. As the structure was of relatively low quality, its metric parameters will not be further discussed.

In contrast, the reaction of the chromium(II) precursor with two equivalents of bulkier mesityl azide (per Cr) produces a paramagnetic brown solution, containing approximately one equivalent of unreacted azide. Similarly, treating chromium(II) precursor with two equivalents of 1-adamantyl azide also produces paramagnetic product, along with unreacted azide. We were not able to obtain X-ray quality crystals of the products. The formation of trigonal Cr(IV) mono(imido) complexes is proposed, based on similar formation of brown paramagnetic Cr^IV^(OC*^t^*Bu_2_Ph)_2_(NMes) and Cr^IV^(OC*^t^*Bu_2_Ph)_2_(NAd) upon reaction of [Cr(OC*^t^*Bu_2_Ph)_2_] with excess azide. We note that selective formation of Cr(IV) mono(imido) complexes upon reaction with bulkier organoazides was also observed for the chromium(II) system ligated by two monodentate siloxides, [Cr(OSi*^t^*Bu_3_)_2_] [40].

In addition to the reactions of Cr(II) precursor with organoazides, we have also studied its reactivity with azobenzene, with the goal to produce Cr(VI) bis(imido) via azobenzene cleavage. Cleavage of the N=N double bond in azoarenes at a single metal center is rare [41,42,43,44]. Heating the mixture of complex **2** with azobenzene to 60 °C in C_6_D_6_ for 24 h did not lead to the formation of Cr^VI^[OO]^Ph^(NPh)_2_ as indicated by ^1^H NMR spectroscopy.

Next, the reactivity of the Cr(II) bis(alkoxide) precursor with diazoalkanes was investigated, targeting formation of Cr(IV)/Cr(VI)-alkylidene complexes. Cr–alkylidene complexes are rare [26,45,46], and their reactivity is relatively unexplored. The related cobalt(II) bis(alkoxide) complex Co(OC*^t^*Bu_2_Ph)_2_(THF)_2_ formed high-valent cobalt-alkylidene upon reaction with N_2_CPh_2_ and N_2_C(Ph)(CO_2_Me) [47], whereas the corresponding iron complex Fe(OC*^t^*Bu_2_Ph)_2_(THF)_2_ reductively coupled diazoalkanes/diazoesters via terminal nitrogens to form bridging tetrazene species [48]. A different reactivity mode was observed for the chromium bis(alkoxide). Treatment of **2** with diphenyldiazomethane results in the formation of brown Cr[OO]^Ph^(NNCPh_2_)_2_ species (**6**), which was characterized by X-ray crystallography. The complex appears unstable in both solid state and solution. Our repeated attempts to collect the NMR spectrum of the X-ray quality crystals produced spectra with varying but significant decomposition products (benzophenone azine, free ligand); peaks attributable to the complex were broad and not informative. Wolczanski and coworkers have reported similar reactivity of Cr_2_(OSi*^t^*Bu_3_)_4_ with N_2_CPh_2_ to yield Cr(OSi*^t^*Bu_3_)_2_(N_2_CPh_2_)_2_ complex [40] and their complex also demonstrated limited stability.

The structure of **6** and selected metrics are given in Figure 3. The chromium center exhibits distorted tetrahedral geometry. Interestingly, the Cr–N bonds are different, one being significantly shorter (1.645(3) Å) than the other (1.716(3) Å). The shorter bond could be indicative of a higher metal-nitrogen bond order (triple), while the longer one suggests a metal-nitrogen double bond. The Cr–N–N bond angles of the [NNCPh_2_] functionality are consistent with these formulations: for the triply bonded [Cr≡N1-N3] hydrazido, the angle is closer to linear (159.8(2) °), whereas for the doubly bonded [Cr=N2-N4] the angle is smaller (131.2(2) °). The N1-N3 and N2-N4 bonds are similar (1.304(4) vs. 1.301(4) Å), being intermediate between single and double. Similar dependence of the Cr–N–N angle within diphenylmethylenehydrazido functionalities on the chromium-nitrogen bond order was reported by Wolczanski, Cundari, and coworkers for the related Cr(OSi*^t^*Bu_3_)_2_(=N_2_CPh_2_)_2_ [40]. The major difference between the present structure and the structure reported by Wolczanski is that in Cr(OSi*^t^*Bu_3_)_2_(=N_2_CPh_2_)_2_ the Cr–N bonds were similar (1.664(2) and 1.666(2) Å), leading to similar Cr–N–N bond angles (150.2(2) and 150.4(3) °).

**Figure 4:**
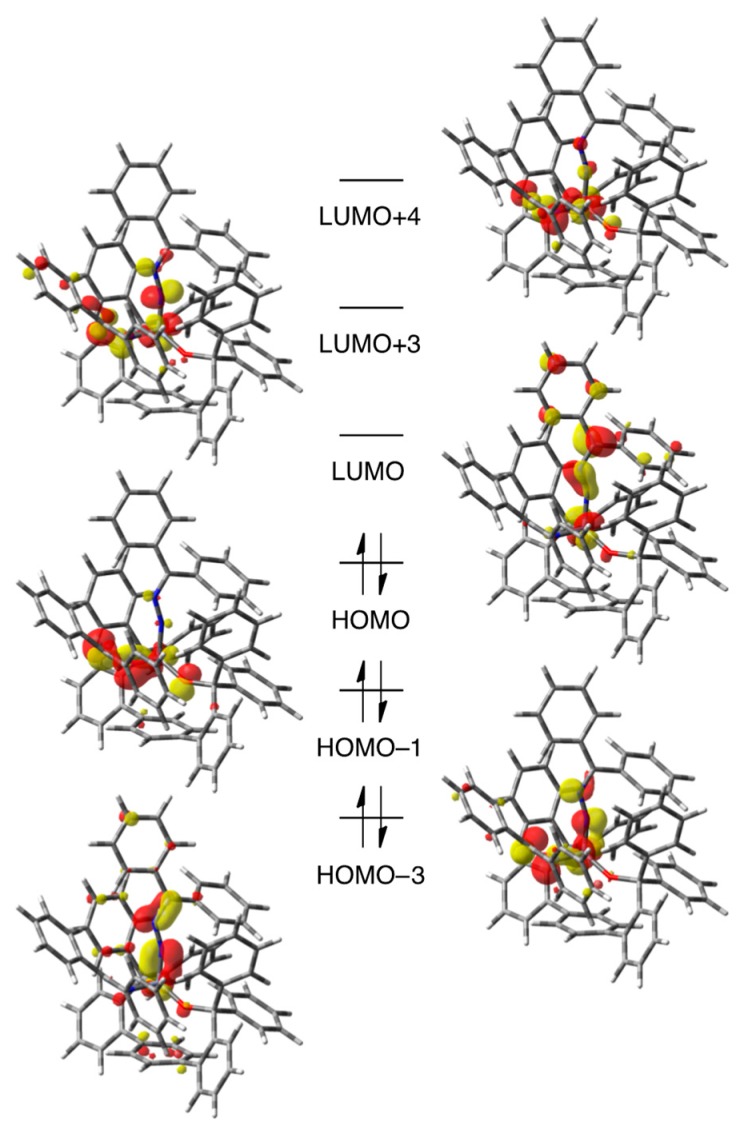
Frontier orbital diagram for **6_S_** at the BP86-D3/def2-SVP level of theory. Orbitals are plotted with an isosurface value of 0.05 au.

DFT calculations on **6** were performed to analyze the electronic structure of this species. Singlet (**6_S_**), triplet (**6_T_**), and quintet (**6_Q_**) states were optimized and the singlet was found to be lowest in free energy followed by the triplet (+15.1 kcal/mol) then the quintet (+33.9 kcal/mol). Only the optimized structure of **6_S_** is consistent with the experimental X-ray structure (Table S3, SI) and has no unpaired electrons. As the localized frontier orbitals in Figure 4 demonstrate, there are two -π-bonding interactions with the more linear diazoalkane ligand (HOMO–1/LUMO+3, HOMO–3/LUMO) compared to one with the less linear diazoalkane ligand (HOMO/LUMO+4), though the HOMO–1/LUMO+3 pair shows interaction with both diazoalkane ligands. The modest difference in Mayer bond orders of 1.61 vs. 1.52 for Cr–N1 vs. Cr–N2 suggests that both Cr–N bonds are between doubly and triply bonded, but that bonding to N1 is stronger.

### 2.3. Reactivity of Cr(II) with CO, CNR and NO^+^

Following the investigation of the reactivity of Cr(II) bis(alkoxide) precursor with organoazides and diazoalkanes, its reactivity with isocyanides, carbon monoxide, and nitrosonium was explored next. It was previously shown that Cr_2_(OC*^t^*Bu_2_Ph)_4_ reacted with isocyanides to form Cr^II^(OC*^t^*Bu_2_Ph)_2_(CNR)_4_ (R = 2,6-Me_2_C_6_H_3_); formation of Cr^II^(OC*^t^*Bu_2_Ph)_2_(CNR)_4_ enabled subsequent development of the catalytic cycle for the production of carbodiimides. Hoping that the more stable and sterically demanding chelating bis(alkoxide) system will enable better catalytic reactivity in CNR and perhaps CO transfer, reactions of Cr[OO]^Ph^ with xylyl isocyanide and carbon monoxide were investigated. The reactivity of this species with nitrosonium ((NO)(BF_4_)) was also studied, in light of the isoelectronic nature between NO^+^ and CO/CNR.

Addition of excess xylyl isocyanide to the light-green solution of **2** (dissolved in THF) forms a brown solution immediately. ^1^H NMR spectrum of the product indicates its paramagnetic nature. Although our multiple attempts to obtain X-ray quality crystals of the isocyanide product did not lead to fruition, the UV–Vis spectrum of the crude product is similar to the UV–Vis spectrum of isolated Cr^II^(OC*^t^*Bu_2_Ph)_2_(CN(2,6-Me_2_C_6_H_3_))_4_ (peaks at approximately 400 and 460 nm, Figure S44) suggesting similarity of the products. In contrast, no color change was observed after treating the solution of **2** in THF with CO. Subsequent recrystallization from a toluene/THF mixture formed instead the crystals of compound **1**, further confirming the lack of reactivity with CO.

**Figure 5:**
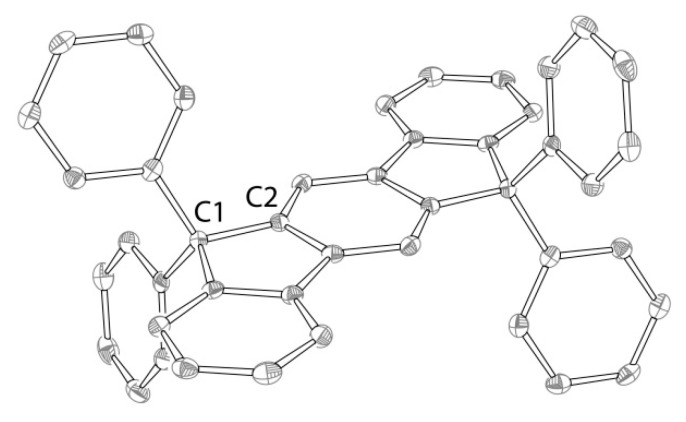
X-ray structure of **7**, 50% probability ellipsoids. H atoms and co-crystallized solvent molecules were omitted for clarity.

**Scheme 2:**
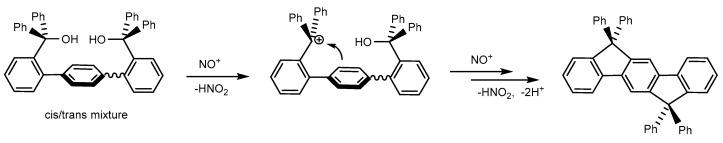
Proposed cyclization mechanism of H_2_[OO]^Ph^.

The reaction of **2** with NO^+^ took an unexpected turn. Addition of (NO)(BF_4_) to the green solution of **2** (THF) led to the development of cloudy yellow solution over the course of several hours. Subsequent recrystallization from THF yields colorless crystals of 6,6,12,12-tetraphenyl-6,12-dihydroindeno[1,2-b]fluorene **7** (Figure 5) isolated in 92% yield. The reaction course does not depend on the presence of Cr: treatment of the ligands precursor H_2_[OO]^Ph^ with (NO)(BF_4_) yields the same product **7**. Cyclization of terphenyl bis(carbinol)s via carbocation intermediates to give various substituted dihydroindenofluorenes had been previously reported [49,50]. We surmise that the reaction proceeds via nitrosonium abstraction of hydroxyl (to yield nitrous acid) followed by cyclization, as described in Scheme 2.

### 2.4. Catalytic Reactivity in the Formation of Carbodiimides

**Scheme 3:**
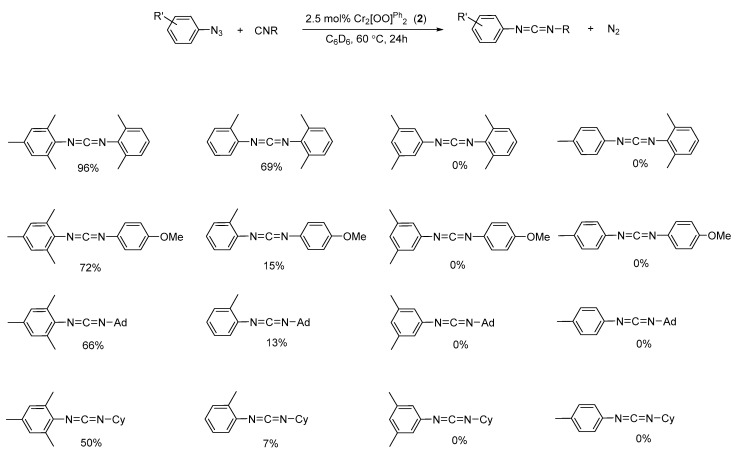
Catalytic formation of carbodiimides mediated by **2**.

Given the ability of the Cr^II^[OO]^Ph^ precursor to undergo stoichiometric reactions with organoazides and isocyanides, we investigated its catalytic reactivity in formation of carbodiimides. A matrix of four representative aryl azides of varying steric bulk (mesityl, 2-methylphenyl, 3,5-dimethylphenyl, and 4-methylphenyl) and four representative isocyanides (xylyl, 4-methoxyphenyl, 1-adamantyl, and cyclohexyl) was investigated (Scheme 3). All reactions were carried out in C_6_D_6_ at 60 °C for 24 h; the nature of the reaction products and their yields were determined by ^1^H NMR spectroscopy. The outcome of the catalytic reaction appears to be a primary function of the organoazide. Catalytic formation of carbodiimides is observed for the bulkier mesityl azide. The reaction of mesityl azide with xylyl isocyanide gives nearly quantitative yield; good to moderate yields (72–50%) are observed for other isocyanides. For the *ortho*-monosubstituted 2-methylphenyl azide, the reaction with xylyl isocyanide produces carbodiimide in a good yield, whereas other isocyanides give low yields of the corresponding products. No carbodiimide formation is observed for 3,5-dimethylphenyl azide and 4-methylphenyl azide. These results are rationalized by the nature of the chromium–imido intermediate, as was described in our previous study [31]. Whereas bulkier azide forms a chromium(IV)–imido, which is capable of isocyanide coordination and subsequent N-C bond formation, tetrahedral chromium(VI) bis(imido) (formed with non-bulky aryl azides) does not coordinate an isocyanide and therefore does not catalyze N-C bond formation.

## 3. Materials and Methods

### 3.1. General

CrCl_2_ and LiN(Si(CH_3_)_3_)_2_ were purchased from Strem chemicals and Sigma-Aldrich, respectively, and used as received. Previously reported methods were used to synthesize Cr(N(SiMe_3_)_2_)_2_(THF)_2_ and the ligand precursor H_2_[OO]^Ph^ [35,51]. Deuterated solvents were purchased from Cambridge Isotope Laboratories and stored over 3 Å molecular sieves. Non-deuterated solvents were purchased from Sigma-Aldrich chemicals and purified using MBraun solvent purification system. Characterization of compounds was carried out using ^1^H and ^13^C NMR spectroscopy, high-resolution mass spectrometry and elemental analysis. Selected chemicals were characterized by X-ray crystallography. NMR spectra of carbodiimides and complexes were recorded using Agilent 400 MHz and Agilent DD2 600 MHz spectrometers, respectively, in C_6_D_6_ at room temperature at the Lumigen Instrument Center. Chemical shifts and coupling constants (J) are reported in parts per million and Hertz, respectively. Elemental analyses were carried out by Midwest Microlab LLC under air-free conditions. A Thermo Fisher Scientific LTQ Orbittrap XL mass spectrometer at the Lumigen Instrument Center was used for high resolution mass spectra. IR spectra of powdered samples were recorded on a Shimadzu IR Affinity-1 FT-IR spectrometer outfitted with a MIRacle10 attenuated total reflectance accessory with a monolithic diamond crystal stage and pressure clamp. A Shimadzu UV-1800 spectrometer was used to collect UV–Vis spectra. All air-sensitive reactions were carried out in a nitrogen-filled glovebox.

### 3.2. Synthesis of Cr Complexes ***1***–***6*** and Compound ***7***

**Cr_2_([OO]^Ph^)_2_ and Cr[OO]^Ph^(THF)_2_ (1 and 2).** A solution of ligand precursor H_2_[OO]^Ph^ (0.034 g, 0.057 mmol) in THF (5 mL) was added to a solution of Cr(N(SiMe_3_)_2_)_2_(THF)_2_ (0.030 g, 0.057 mmol) in THF (5 mL). The color of the reaction changed from purple to light green over a course of 4 h. The solvent was removed under vacuum and the resulting residue recrystallized in DCM-THF mixture at −35 °C to obtain light green crystals of **2** in 79% yield (0.029 g, 0.023 mmol). Anal. Calcd for C_88_H_64_Cr_2_O_4_: C, 81.97, H, 5.00. Found: C, 80.82, H, 6.23. λ_max_ (ε_M_ (L^−1^ cm^−1^ mol^−1^)) 706 nm (28000). IR (cm^−1^): 2947 (m), 2337 (m), 1334 (w), 1151 (w), 987 (m), 840 (m), 756 (s), 702 (s). A similar procedure was used to synthesize Cr[OO]^Ph^ (THF)_2_ (**1**) but recrystallization was done in toluene with small amount of THF. Calcd for C_52_H_46_CrO_4_: C, 79.17, H, 6.13. Found C, 78.37, H, 5.39. IR (cm^−1^) 2916 (m), 2353 (m), 1458 (m), 1365 (w), 1319 (w), 1149 (w), 1026 (m), 995 (w), 833 (m), 779 (s), 740 (m), 709 (m), 686 (m).

**Figure 6:**
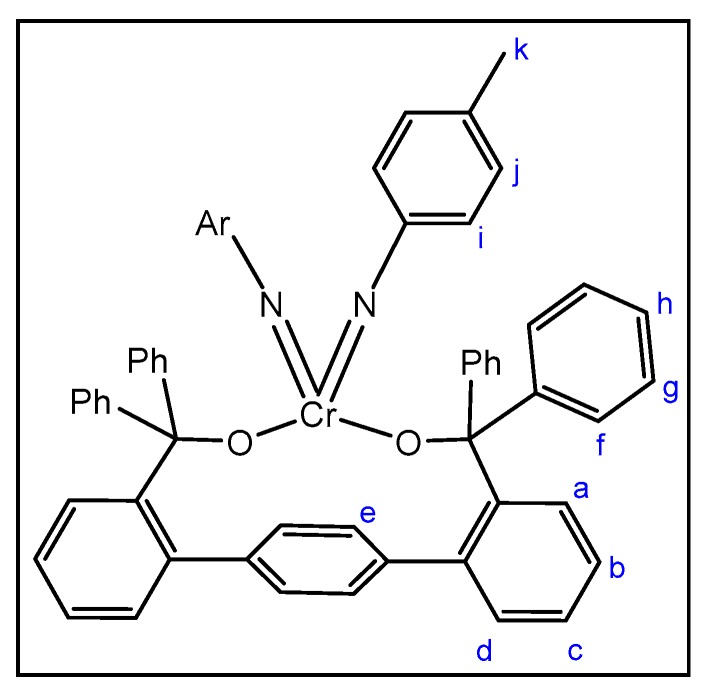
Assignment of ^1^H NMR signals for **3** (see previous paragraph for details).

**Synthesis of Cr[OO]^Ph^(N(4-CH_3_C_6_H_4_))_2_ (3, Figure 6).** A solution of 4-azidotoluene (0.020 g, 0.15 mmol) in THF was added to a 5 mL solution of Cr_2_([OO]^Ph^)_2_ (0.050 g, 0.038 mmol). Upon addition, the solution color changed from light green to brown; the release of N_2_ was also observed. The reaction was stirred for 24 h. The solvent was removed under vacuum and the solid residue was recrystallized from ether-THF mixture at −35 °C to afford dark brown crystals in 69% yield (0.046 g, 0.054 mmol). ^1^H NMR (600 MHz, C_6_D_6_, 298 K): δ 7.58 (d, 2H, *J_HH_* = 7.6 Hz, H_a_), 7.6 (d, 8H, *J_HH_* = 6.1 Hz, H_f_), 7.41 (s, 4H, H_e_), 7.10-7.12 (m, 2H, H_b_), 7.03-7.07 (m, 12H, H_g_ & H_h_), 6.93-6.96 (m, 4H, H_c_ & H_d_), 6.53 (d, 4H, *J_HH_* = 7.6 Hz, H_j_, 6.15 (d, 4H, *J_HH_* = 8.2 Hz, H_i_), 1.92 (s, 6H, H_k_). ^13^C NMR (150 MHz, C_6_D_6_, 298 K): δ 160.69, 151.19, 146.27, 142.97, 142.93, 137.30, 131.11, 130.92, 129.17, 128.22, 127.92, 127.55, 127.52, 127.37, 126.39, 125.57, 124.26, 95.51, 20.72.

**Figure 7:**
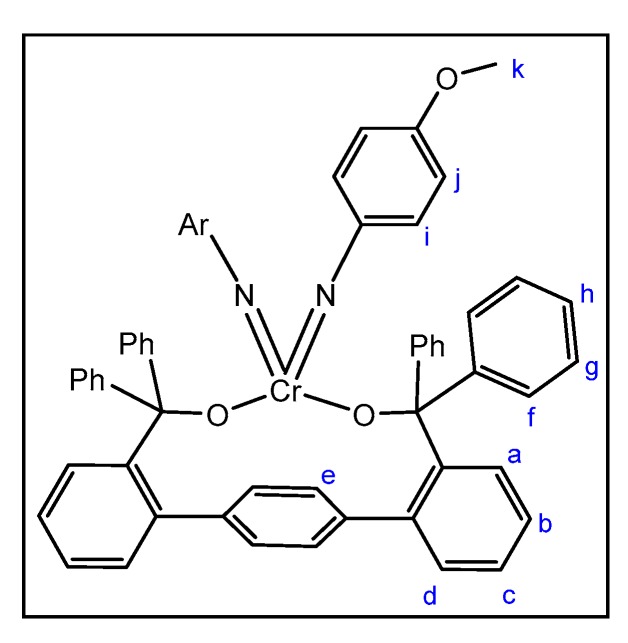
Assignment of ^1^H NMR signals for **4** (see previous paragraph for details).

**Cr[OO]^Ph^(N(4-CH_3_OC_6_H_4_))_2_ (4, Figure 7).** A solution of 4-azidoanisole (0.023 g, 0.15 mmol in THF) was added to a 5 mL THF solution of Cr_2_([OO]^Ph^)_2_ (0.050 g, 0.038 mmol). Color changed from light green to brown with release of N_2_ and the reaction was stirred for 24 h. The solvent was removed under vacuum and the resulting residue was recrystallized from ether/THF mixture at −35 °C to give a brown solid, which was washed with ether to afford the product in 54% yield (0.54 g, 0.043 mmol). ^1^H NMR (600 MHz, C_6_D_6_, 298 K): δ 7.57–7.61 (m, 10H, H_a_ and H_f_), 7.44 (s, 4H, H_e_), 7.10–7.12 (m, 2H, H_b_), 7.02–7.08 (t, 8H, *J*_HH_ = 7.0 Hz, H_g_), 7.01–7.03 (t, 4H, *J*_HH_ = 7.0 Hz, H_h_), 6.97 (m, 4H, H_c_ and H_d_), 6.31 (d, 4H, *J*_HH_ = 8.8 Hz, H_j_), 6.27 (d, 4H, *J*_HH_ = 8.8 Hz, H_i_), 3.07 (s, 6H, H_k_). ^13^C NMR (150 MHz, C_6_D_6_, 298 K): δ 158.54, 157.54, 151.30, 146.41, 143.00, 131.08, 130.95, 129.21, 127.92, 127.71, 127.54, 127.52, 127.31, 126.32, 126.13, 125.54, 112.79, 95.07, 54.91.

**Figure 8:**
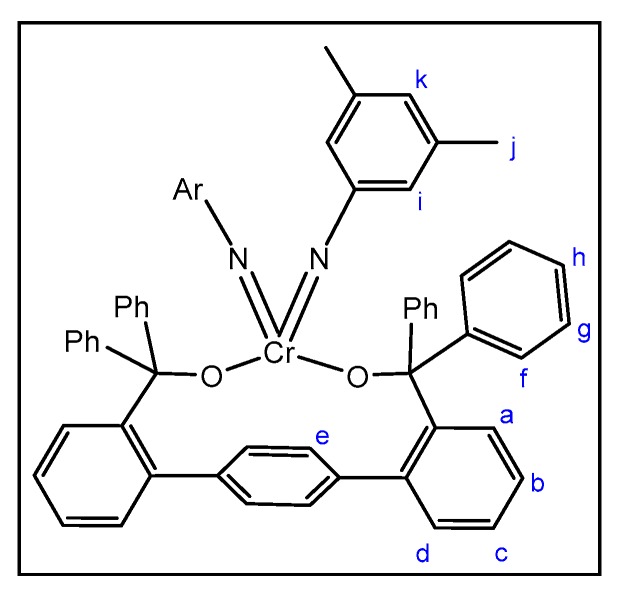
Assignment of ^1^H NMR signals for **5** (see previous paragraph for details).

**Cr[OO]^Ph^(N(3,5-Me_2_C_6_H_3_))_2_ (5, Figure 8).** A solution of 3,5-dimethylphenyl azide (0.022 g, 0.15 mmol) in THF) was added to THF solution (5 mL) of Cr_2_([OO]^Ph^)_2_ (0.050 g, 0.038 mmol). Color changed from light green to brown with release of N_2_ and the reaction was stirred for 24 h. The solvent was removed under vacuum and the residue was recrystallized from ether-THF at −35 °C to form a brown solid, which was washed with ether to afford the product in 57% (0.038 g, 0.043 mmol). ^1^H NMR (600 MHz, C_6_D_6_, 298 K) δ 7.57–7.61 (m, 10H, H_a_ and H_f_), 7.42 (s, 4H, H_e_), 7.09–7.11, (2H, t, *J*_HH_ = 6.4 Hz, H_b_), 7.00–7.08 (m, 12H, H_g_ and H_h_), 6.93 (t, 2H, *J_HH_* = 7.6 Hz, H_c_), 6.90 (d, 2H, *J_HH_* = 8.8 Hz, H_d_), 6.31 (s, 2H, H_k_), 5.84 (s, 4H, H_i_), 1.82 (s, 12H, H_j_). ^13^C NMR (150 MHz, C_6_D_6_, 298K): δ 162.5, 151.20, 146.26, 142.97, 136.83, 131.11, 130.94, 129.21, 129.14, 128.98, 128.19, 127.92, 126.96, 126.75, 126.44, 126.20, 125.55, 121.99, 95.52. 20.37.

**Cr[OO]Ph(N_2_CPh_2_)_2_ (6).** A (5 mL) THF solution of Cr_2_([OO]^Ph^)_2_ (0.030 g, 0.023 mmol) was reacted with diphenyldiazomethane (0.018 g, 0.092 mmol) for 4 h. The light green color of the complex changed immediately to purple. The solution was stirred for 6 h at RT. The solvent was removed under vacuum and the residue was recrystallized from ether-THF mixture at −35 °C. Brown color crystals were isolated in 52% yield (0.025 g, 0.024 mmol). Calcd for C_70_H_52_CrN_4_O_2_×C_4_H_8_O×0.5C_6_H_12_: C, 80.60, H, 5.80. Found C, 79.52, H, 5.57.

**Figure 9:**
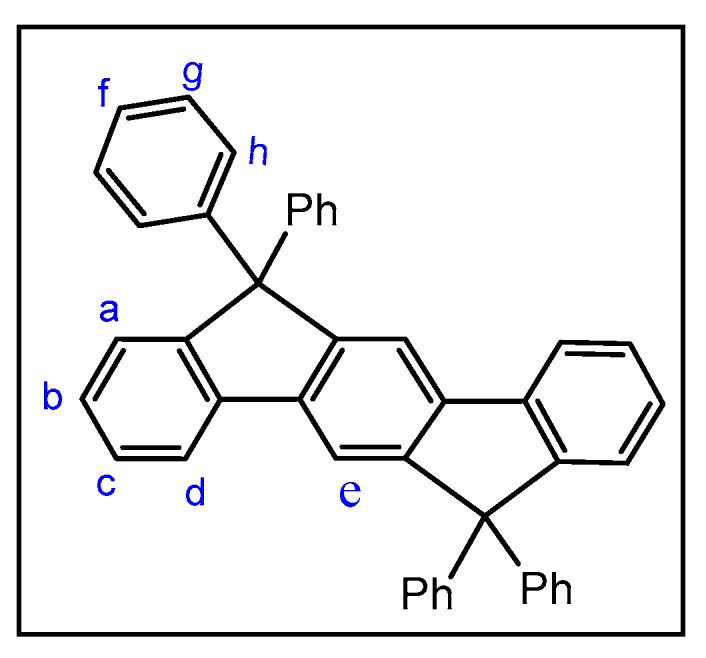
Assignment of ^1^H NMR signals for **7** (see previous paragraph for details).

**6,6,12,12-tetraphenyl-6,12-dihydroindeno[1,2-b]fluorene (7, Figure 9).** 1. Reaction of Cr_2_([OO]^Ph^)_2_ with (NO)(BF_4_): A solution of (NO)(BF_4_) (0.010 g, 0.093 mmol) in THF (5 mL) was added to a THF solution (5 mL) of Cr_2_([OO]^Ph^)_2_ (0.030 g, 0.023 mmol). The green color solution became cloudy and turned yellow over the course of 6 h. The solvent was removed under vacuum and the residue recrystallized with ether-THF mixture. Colorless crystals were obtained in 92% yield (0.023 g, 0.040 mmol). 2. Reaction of H_2_[OO]^Ph^ with (NO)(BF_4_): A solution of (NO)(BF_4_) (0.012 g, 0.1 mmol) in THF (5 mL) was added to a THF solution (5 mL) of H_2_[OO]^Ph^ (0.030 g, 0.051 mmol). The colorless solution turned light brown over a period of 6 h. The solvent was removed under vacuum and the residue recrystallized in CH_2_Cl_2_. Colorless crystals of **7** were obtained in 77% yield (0.021 g, 0.039 mmol). ^1^H NMR (600 MHz, CD_2_Cl_2_, 298 K): δ 7.81 (s, 2H, He), 7.71 (d, *J*_HH_ = 7.6 Hz, 2H, H_a_), 7.38 (d, 2H, *J*_HH_ = 7.6, H_d_), 7.32 (t, 2H, *J*_HH_ = 7.6 Hz, H_b_), 7.26 (m, 22H, H_c_, H_f_, H_g_, H_h_). Related compound (lacking the phenyl substituents) and its NMR characterization were previously reported [50]. ^13^C NMR (125 MHz, CD_2_Cl_2_, 298K): δ 151.48, 151.12, 145.92, 140.21, 139.93, 128.27, 128.13, 127.58, 127.48, 126.69, 126.07, 120.16, 117.81, 65.24. HRMS (m/z): Calcd, [C_44_H_30_]^+^ 558.2347, found 558.2333.

**General Procedure for Catalytic Studies.** C_6_D_6_ solution containing 20 equivalents of an organoazide, 24 equivalents of an isocyanide and 2 equivalents of 1,3,5-trimethoxybenzene (internal standard) was mixed with 2.5 mol% catalyst in C_6_D_6_. The reaction was heated at 60 °C for 24 h. The products were identified, and the yields were calculated using ^1^H NMR spectroscopy.

### 3.3. X-ray Crystallographic Details

Structures of complexes **1**, **2**, **3**, and **6**, and 6,6,12,12-tetraphenyl-6,12-dihydroindeno- [1,2-b]fluorene (**7**) were confirmed by X-ray structure determination; data collection and refinement details are given in Table 1. The crystals were mounted on a Bruker APEXII/Kappa three circle goniometer platform diffractometer equipped with an APEX-2 detector. The structures of **1**, **3**, **6**, and **7** were collected using MoKα (λ = 0.71073 Å) at Wayne State University. The structure of **2** was collected using CuKα radiation (1.54178 Å) in the Center for Crystallographic Research in the Department of Chemistry, Michigan State University. The data were processed and refined using the APEX2 software. Structures were solved by direct methods in SHELXS and refined by standard difference Fourier techniques in the SHELXTL program suite (6.10 v., Sheldrick G. M., and Siemens Industrial Automation, 2000). Hydrogen atoms were placed in calculated positions using the standard riding model and refined isotropically; all other atoms were refined anisotropically. The structure of **6** contained half a molecule of cyclohexane and a molecule of THF in the asymmetric unit. The structure of **7** co-crystallized with CH_2_Cl_2_ which was fully refined. The structure of **3** contained multiple severely disordered THF solvent molecules in the asymmetric unit, leading to the overall poor quality of the diffraction data. We had only limited success in modelling the disorder in THF solvent, thus, SQUEEZE program was applied to remove the disordered solvent. One of the *para*-tolyl groups in the structure of **3** was found to be disordered; the disorder was successfully modeled by finding two alternative conformations. The structure of **1** was also of relatively poor quality, due to the very small size of the crystals. Despite data collection at 60 s per frame, little diffraction was observed beyond ~1 Å. The structure of **1** contained several toluene molecules per asymmetric unit, one of which was found to be disordered over two conformations. The structure of **2** contained significant amount of highly disordered THF solvent, which was removed using SQUEEZE.

### 3.4. Computational Details

DFT calculations were performed using Gaussian 09 [52]. All calculations were performed at the BP86-D3/def2-SVP level of theory [53,54,55,56,57], employing density fitting, ultrafine grids [58], and Becke–Johnson damping for the dispersion corrections. Optimized structures were confirmed to be minima by ensuring all harmonic frequencies were real. All wavefunctions were verified to be stable. Orbital analyses were performed in GaussView 6.0.16 [59], and Mayer bond orders were calculated using Multiwfn 3.5 [60].

## 4. Conclusions

We have demonstrated that chelating bis(alkoxide) ligand [OO]^Ph^, featuring a terphenyl spacer between the alkoxide donors, forms mononuclear (Cr[OO]^Ph^(THF)_2_) or dinuclear (Cr_2_[OO]^Ph^_2_) Cr(II) complexes, depending on the crystallization conditions. These results suggest that [OO]^Ph^ exhibits a larger steric profile compared with two monodentate alkoxides [OC*^t^*Bu_2_Ph] that formed a Cr_2_(OC*^t^*Bu_2_Ph)_4_ dimer invariably. Reactions of this complex with organoazides form diamagnetic Cr(VI) bis(imido) complexes or paramagnetic Cr(IV) mono(imido) complexes. The outcome of the reaction depends on the sterics of the organoazide: mesityl azide formed Cr(IV) mono(imido) but 3,5-dimethylphenyl/4-methylphenyl azides formed Cr(VI) bis(imido) complexes. With diphenyldiazomethane, a chromium bis(diphenylmethylenehydrazido) product forms. Treatment of the Cr(II) precursor with isocyanide likely forms a Cr(II) isocyanide adduct, whereas no reaction is observed with CO. Isoelectronic nitrosonium abstracts hydroxyl to induce cyclization, producing 6,6,12,12-tetraphenyl-6,12-dihydroindeno[1,2-b]fluorene. Catalytic studies combining mixtures of isocyanides with organozides enable carbodiimide formation only for bulkier (mono- or di-orthosubstituted) aryl azides. This finding is consistent with our previous report, which demonstrated that the C–N bond formation in carbodiimide is preceded by isocyanide coordination to the metal and thus occurs at the Cr(IV) mono(imido) intermediate only. Our future studies will focus on the development of new chelating bis(alkoxide) ligands, which could enable catalytic reactivity for a wider range of substrates.

## Figures and Tables

**Table 1:** X-ray crystallographic details for complexes **1**–**3**, **6**, and compound **7**.

Complex	1	2	3	6	7
formula	C_52_H_46_CrO_4_ × 2.5C_7_H_8_	2C_88_H_64_Cr_2_O_4_	C_58_H_46_CrN_2_O_2_	C_70_H_52_CrN_4_O_2_ × 0.5C_6_H_12_ × C_4_H_8_O	C_22_H_15_ × CH_2_Cl_2_
fw, g/mol	1020.25	2578.80	854.97	1147.34	364.27
crystal system	Triclinic	Triclinic	Monoclinic	Triclinic	Triclinic
space group	*P*-1	*P*-1	*P*21/c	*P*-1	*P*-1
*a* (Å)	9.946 (11)	13.9851 (4)	17.38 (15)	10.1831 (6)	8.7464 (5)
*b* (Å)	14.298 (16))	19.4602 (6)	13.03 (11)	13.0107 (7)	8.7610 (5)
*c* (Å)	19.19 (2)	30.4287 (10)	28.58 (19)	23.0961 (13)	12.4072 (7)
*α* (deg)	83.38 (3)	81.683 (2)	90.00	100.964 (3)	76.995 (2)
*β* (deg)	82.55 (2)	77.609 (2)	127.4 (4)	101.580 (3)	78.928 (2)
*γ* (deg)	82.44 (2)	74.125 (2)	90.00	99.999 (3)	70.505 (2)
*V* (Å^3^)	2668 (5)	7747.2 (4)	5142 (71)	2870.7 (3)	866.13 (9)
*Z*	2	4	4	2	2
*d_calcd_*, g/cm^3^	1.270	1.1055	1.104	1.327	1.397
*μ*, mm^−1^	0.266	2.673	0.263	0.257	0.377
*T* (K)	100(2)	172.99	100 (2)	100 (2)	100 (2)
*2θ*, deg	49.00	128.14	47.44	52.96	55.02
*R_1_^a^* [(I > 2σ)]	0.0981	0.0827	0.0727	0.0730	0.0388
*wR_2_^b^* [(I > 2σ)]	0.2070	0.1875	0.1512	0.1710	0.0803
GOF^c^ (F^2^)	0.973	0.7573	0.881	1.037	1.041

^a^*R*_1_ = ∑||*F*_o_ – |*F*_c_||/∑|*F*_o_|. ^b^ w*R*_2_ = (∑ (*w*(*F*_o_^2^ – *F*_c_^2^)^2^)/∑(*w*(*F*_o_^2^)^2^))^1/2^. ^c^ GOF = (∑*w*(*F*_o_^2^ – *F*_c_^2^)^2^/(*n* – *p*))^1/2^, where *n* is the number of data and *P* is the number of parameters refined.

## Supplementary Materials

The following are available online: NMR, MS, UV–Vis and IR spectra, cif files, and computational details. Cif files of the structures of **1**–**3**, **6**, and **7** were deposited at CCDC under the following numbers: 1970958–1970962.
