# Supplementary material for: Synthesis of Chromium(II) Complexes with Chelating Bis(alkoxide) Ligand and Their Reactions with Organoazides and Diazoalkanes
Source: Molecules. 2020 Jan 9;25(2):273. doi: 10.3390/molecules25020273 (PMC7024303; doi:10.3390/molecules25020273)
Supplement: Supplementary file 1 [file molecules-25-00273-s001.pdf]

Supporting Information for:

Synthesis of Chromium(II) Complexes with Chelating Bis(alkoxide) Ligand and Their Reactions with Organoazides and Diazoalkanes

Sudheer S. Kurup [1], Richard J. Staples [2], Richard L. Lord [3,*] and Stanislav Groysman [1,*]

Table of Contents

1. NMR Spectra 2

2. UV-vis Spectra 41

3. High Resolution Mass Spectrum of **7** 57

4. GC-MS Spectra 58

5. IR Spectra 56

6. Computational data 58

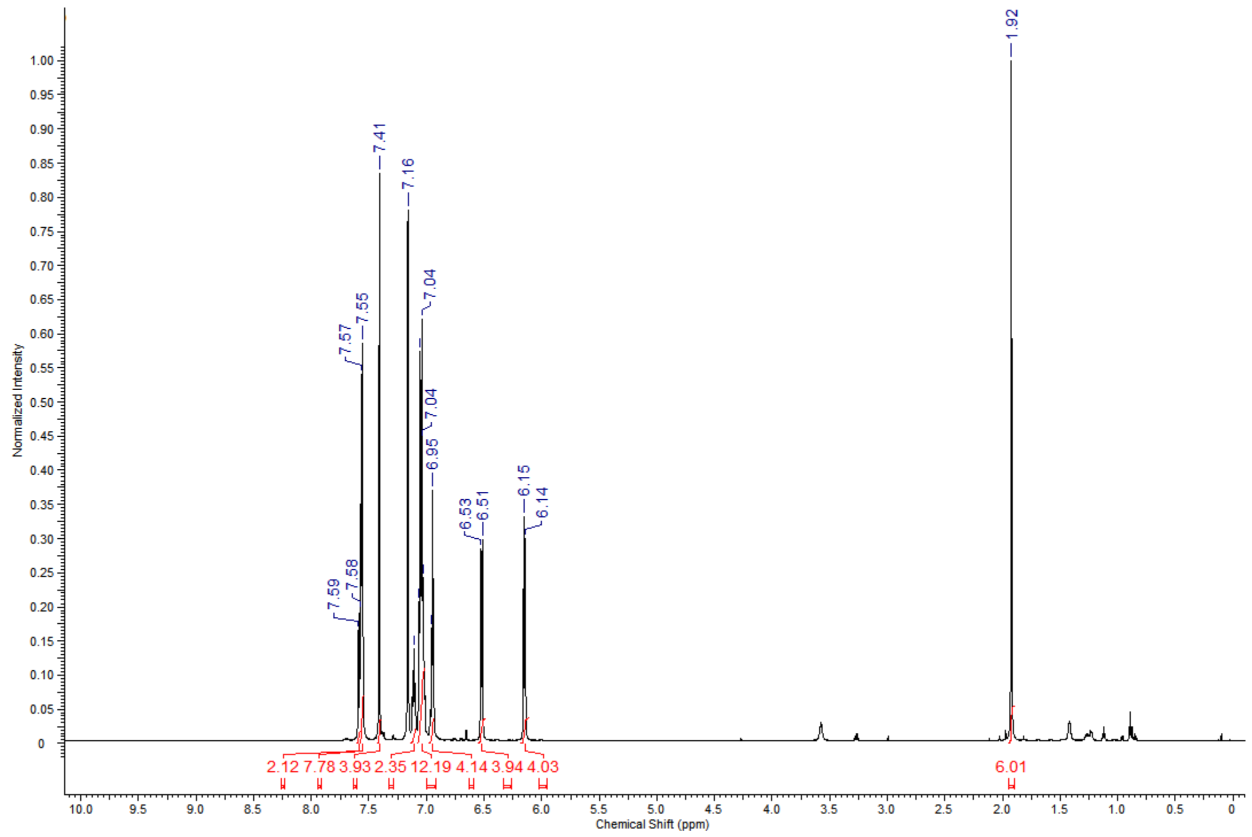

Figure S1. ^{1}H NMR of Cr[OO]^{Ph}(N(4-CH_3Ph))$_2$ (**3**).

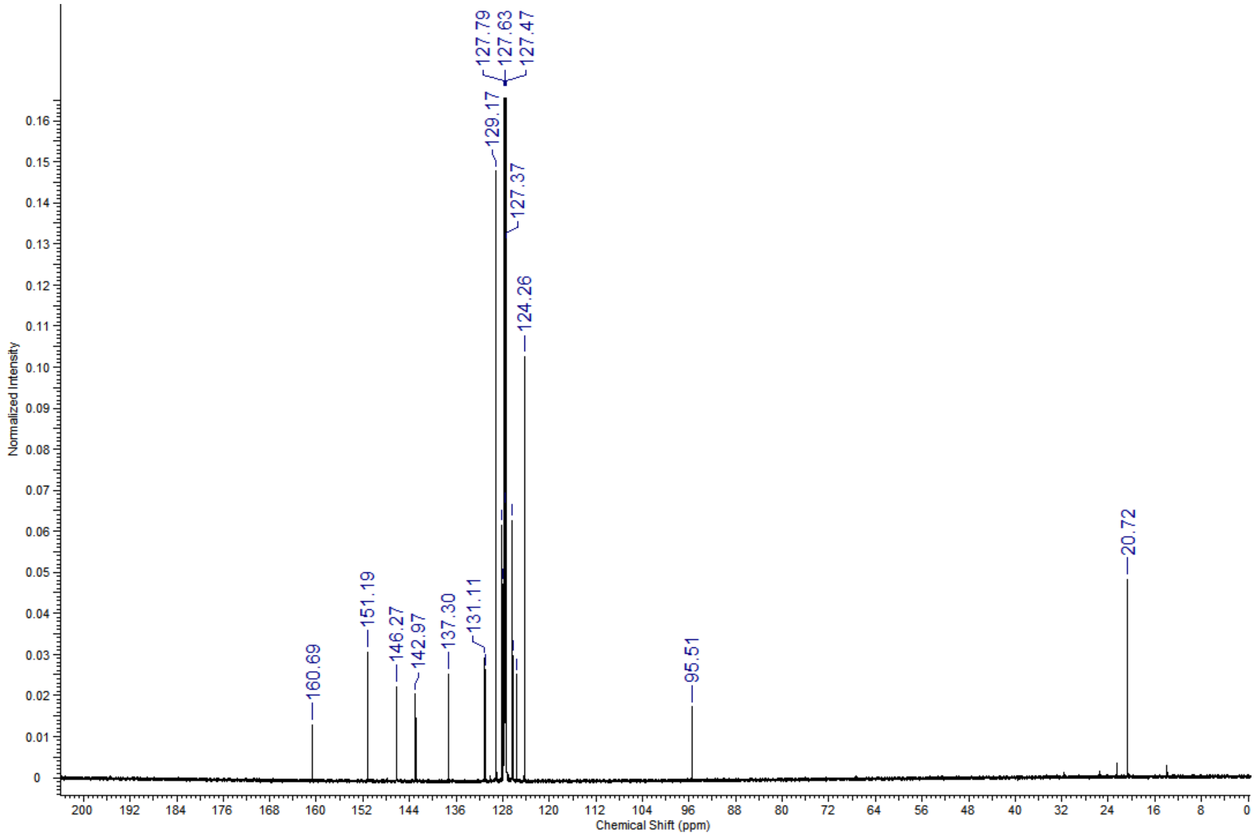

Figure S2. ^{13}C NMR Cr[OO]^{Ph}(N(4-CH_3Ph))$_2$ (**3**).

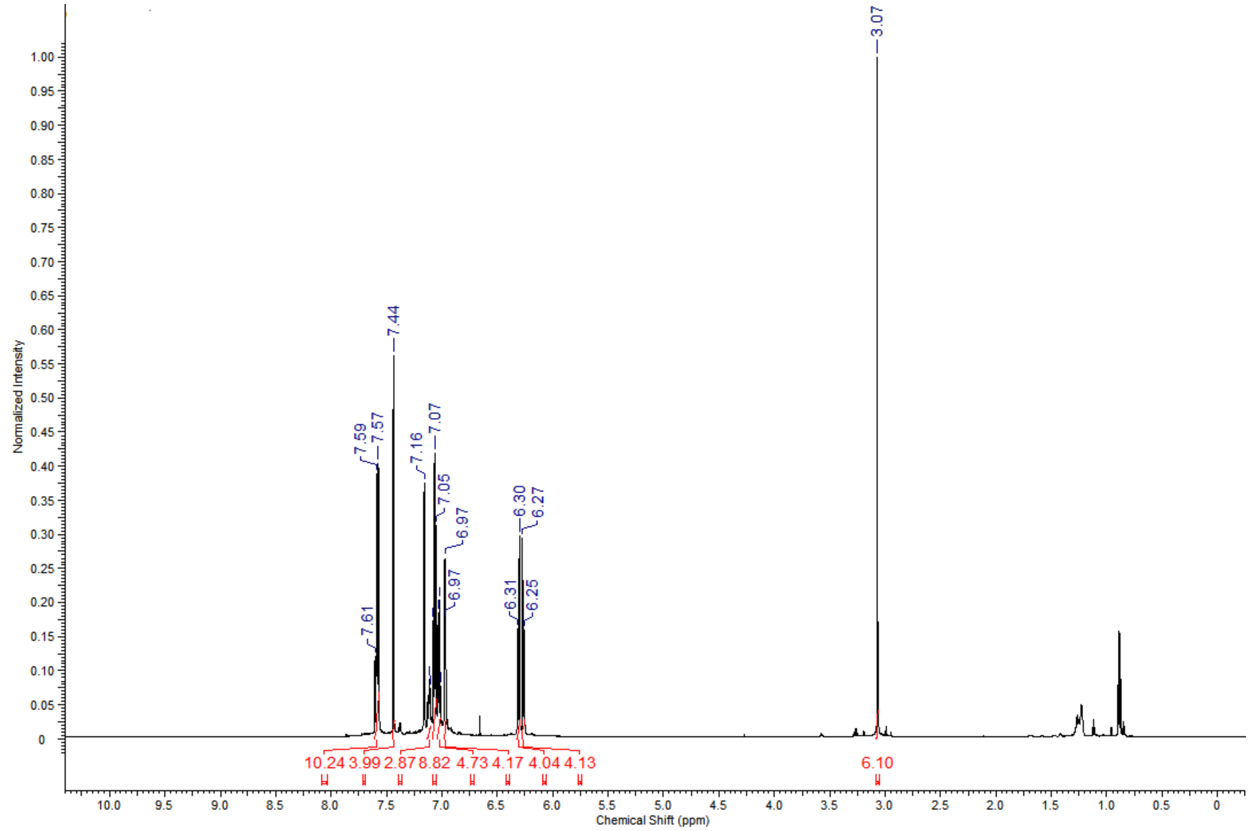

Figure S3. ^{1}H NMR of Cr[OO]^{Ph}(N(4-CH_3OPh))$_2$ (**4**).

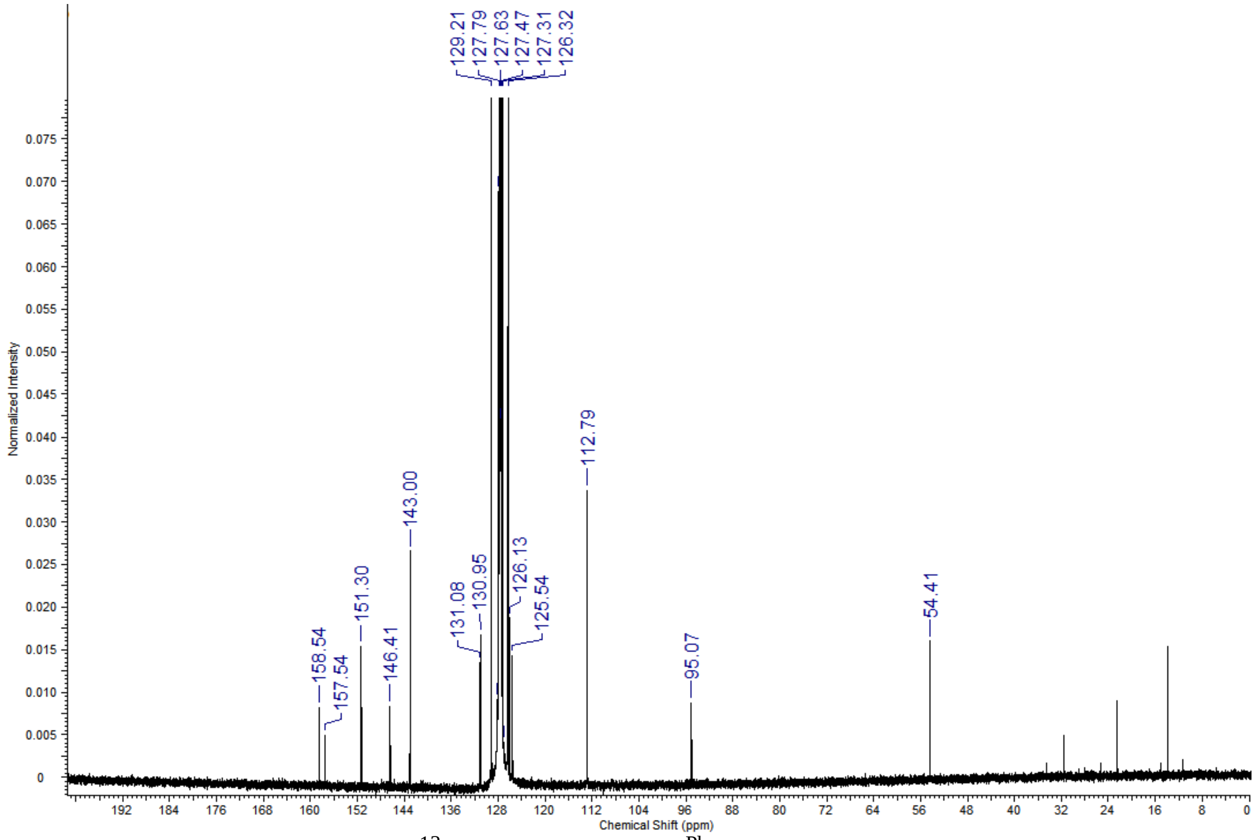

Figure S4. ^{13}C NMR of $Cr[OO]^{Ph}(N(4\text{-}CH_3OPh))_2$ (**4**).

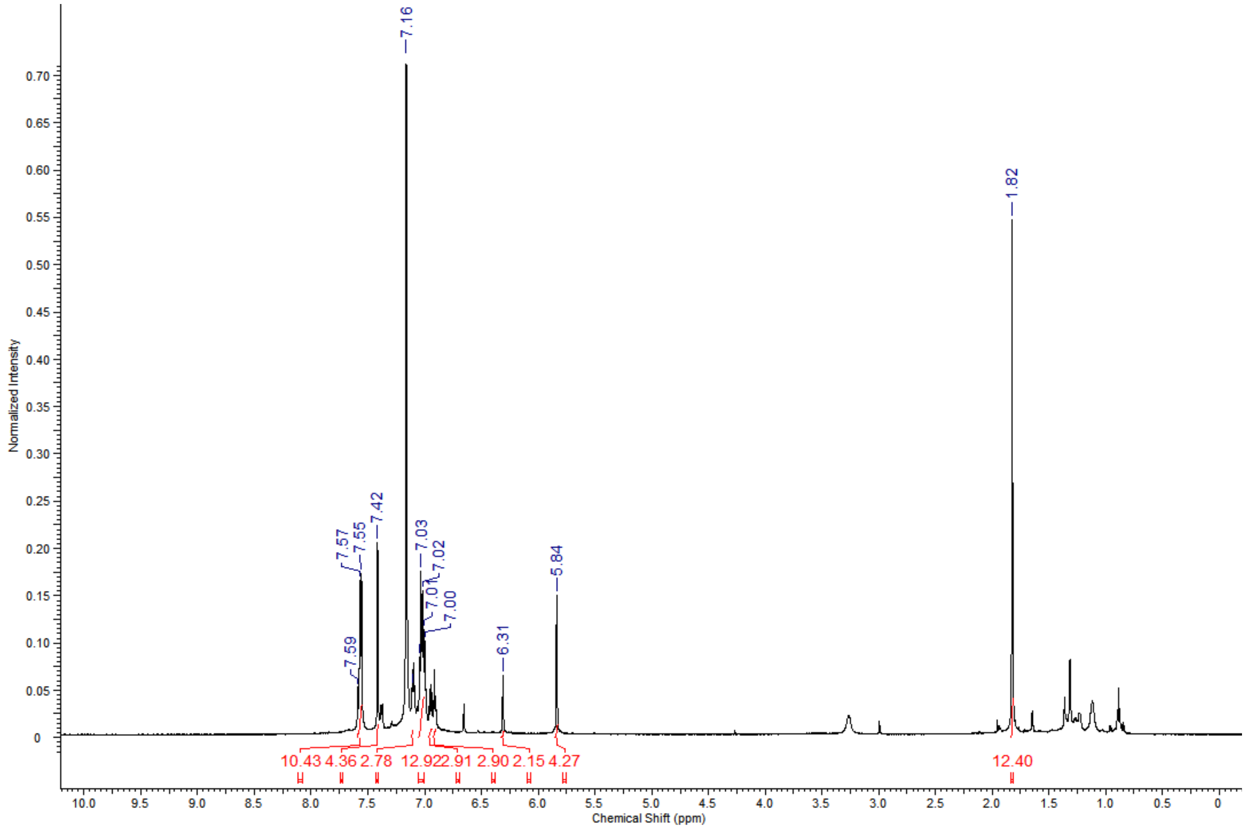

Figure S5. ^{1}H NMR of Cr[OO]^{Ph} (N(3,5-(CH_3)$_2$Ph))$_2$ (**5**).

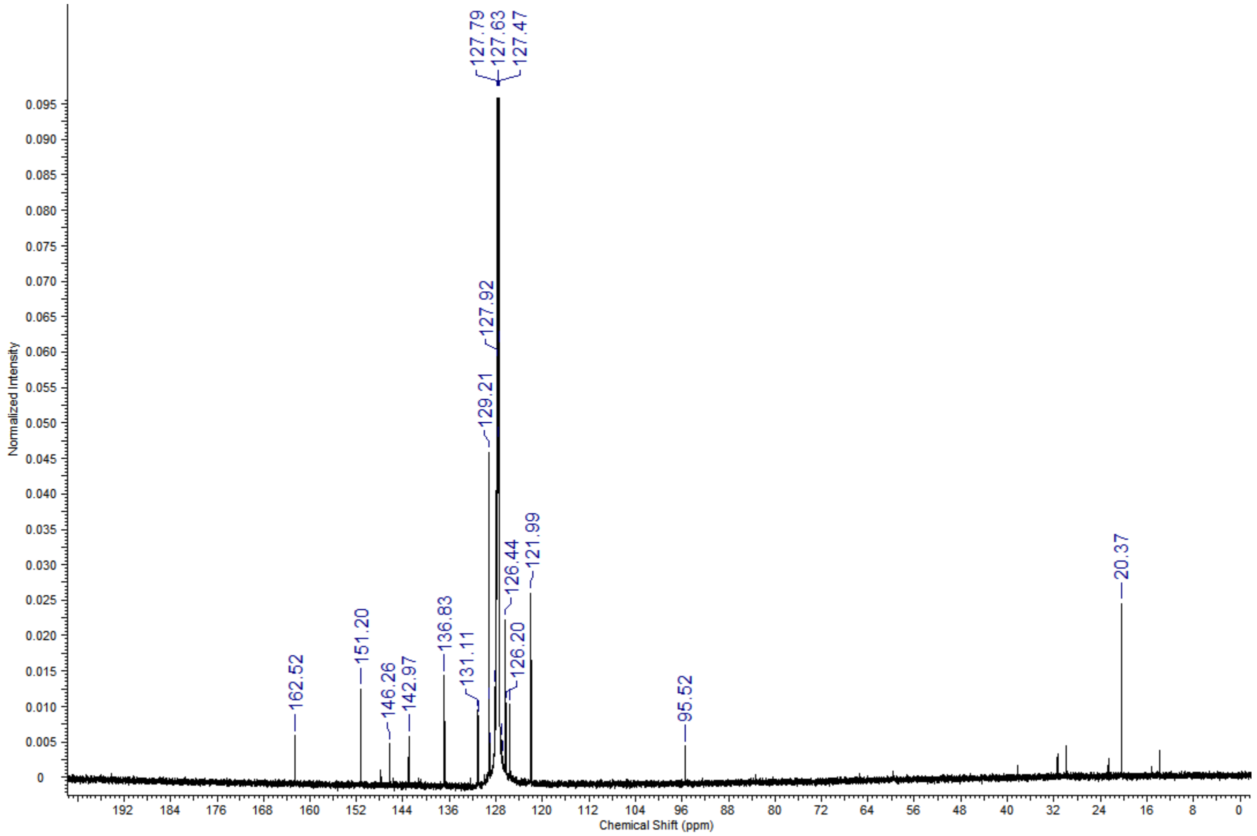

Figure S6. ^{13}C NMR of Cr[OO]^{Ph} (N(3,5-$(CH_3)_2$Ph))$_2$ (**5**).

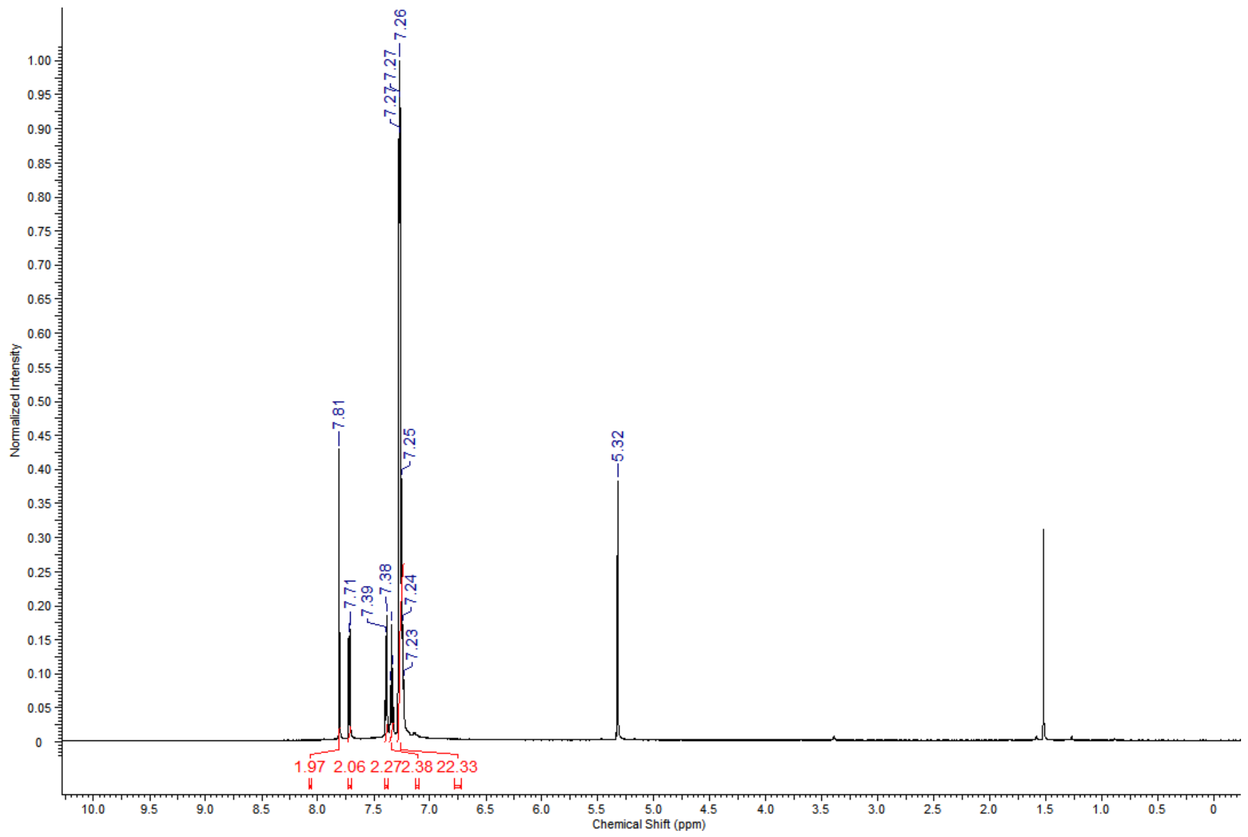

Figure S7. ^{1}H NMR of 6,6,12,12-tetraphenyl-6,12-dihydroindeno[1,2-b]fluorine (**7**).

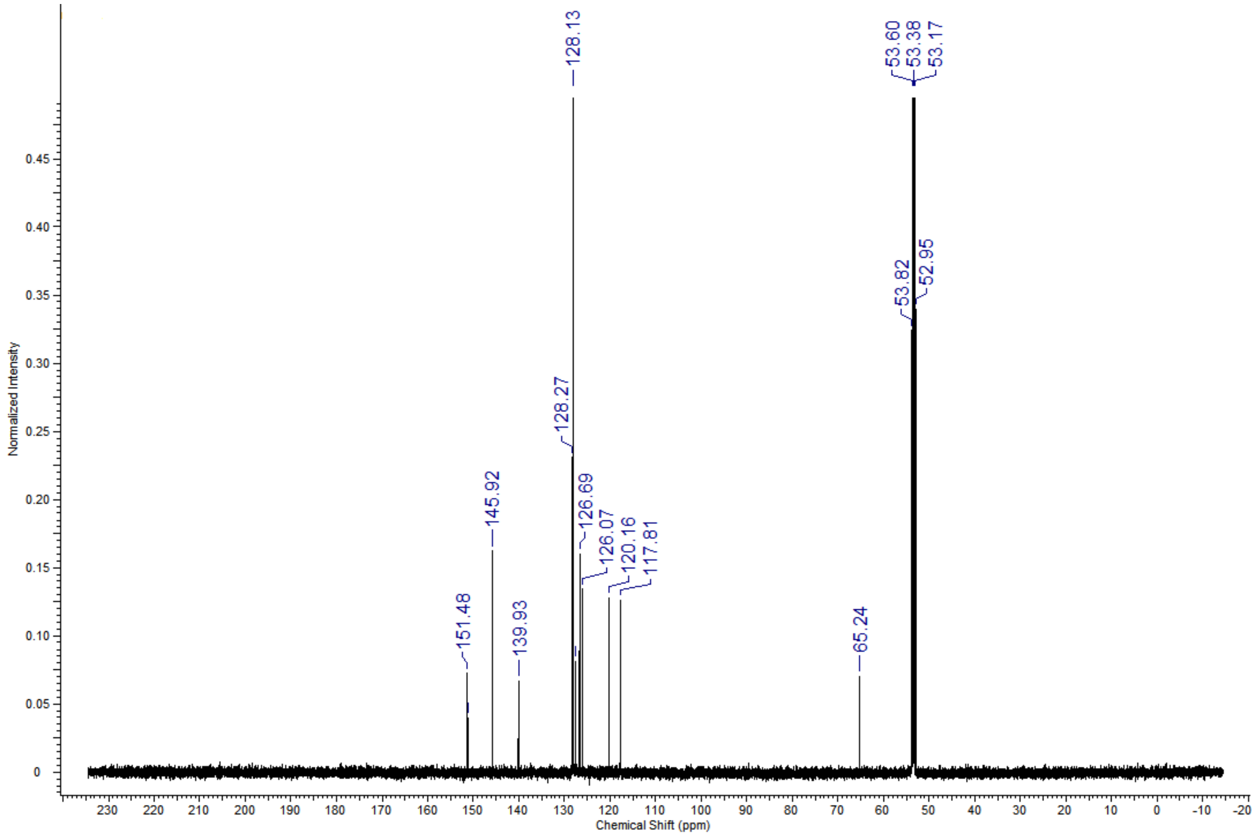

Figure S8. ^{13C} NMR of 6,6,12,12-tetraphenyl-6,12-dihydroindeno[1,2-b]fluorine (**7**).

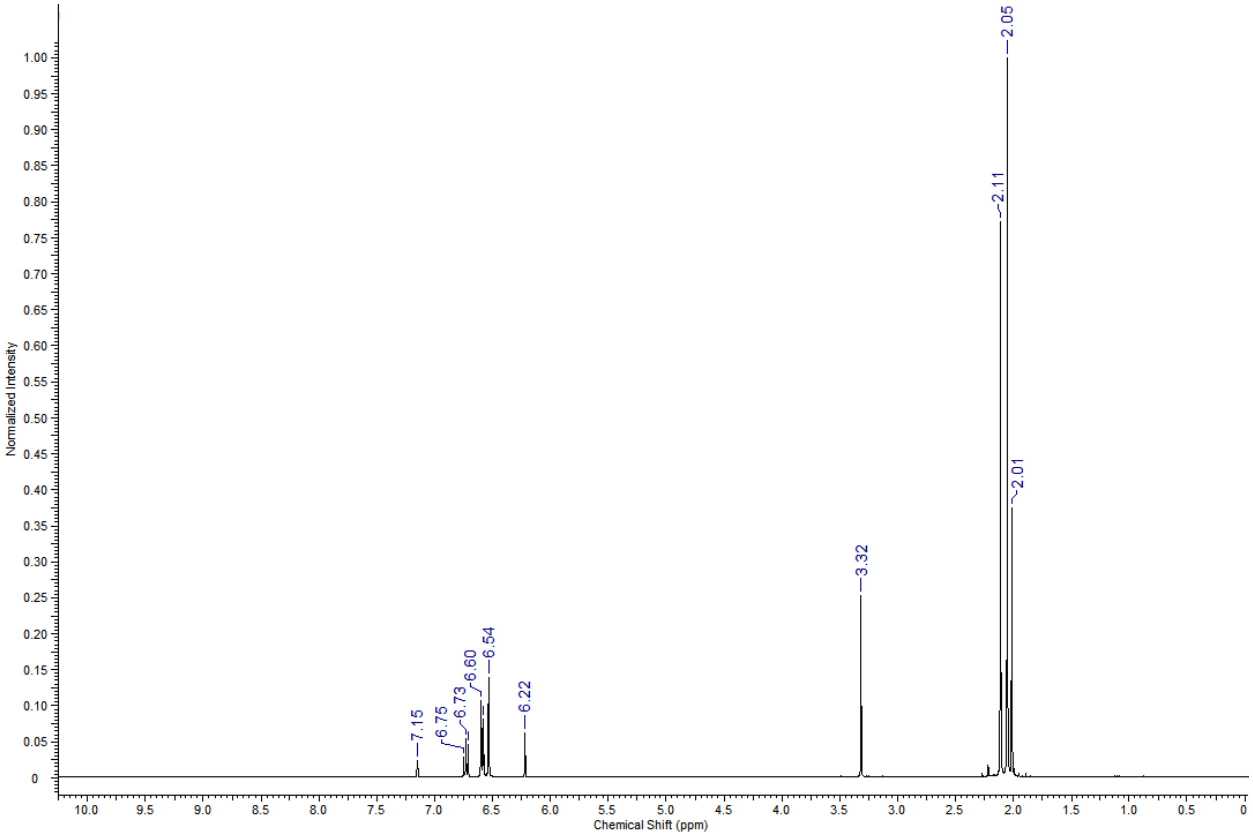

Figure S9. ^{1}H NMR of mesityl azide and xylyl isocyanide prior to the addition of the catalyst (the resonances at 3.32 and 6.22 ppm belong to the internal standard TMB).

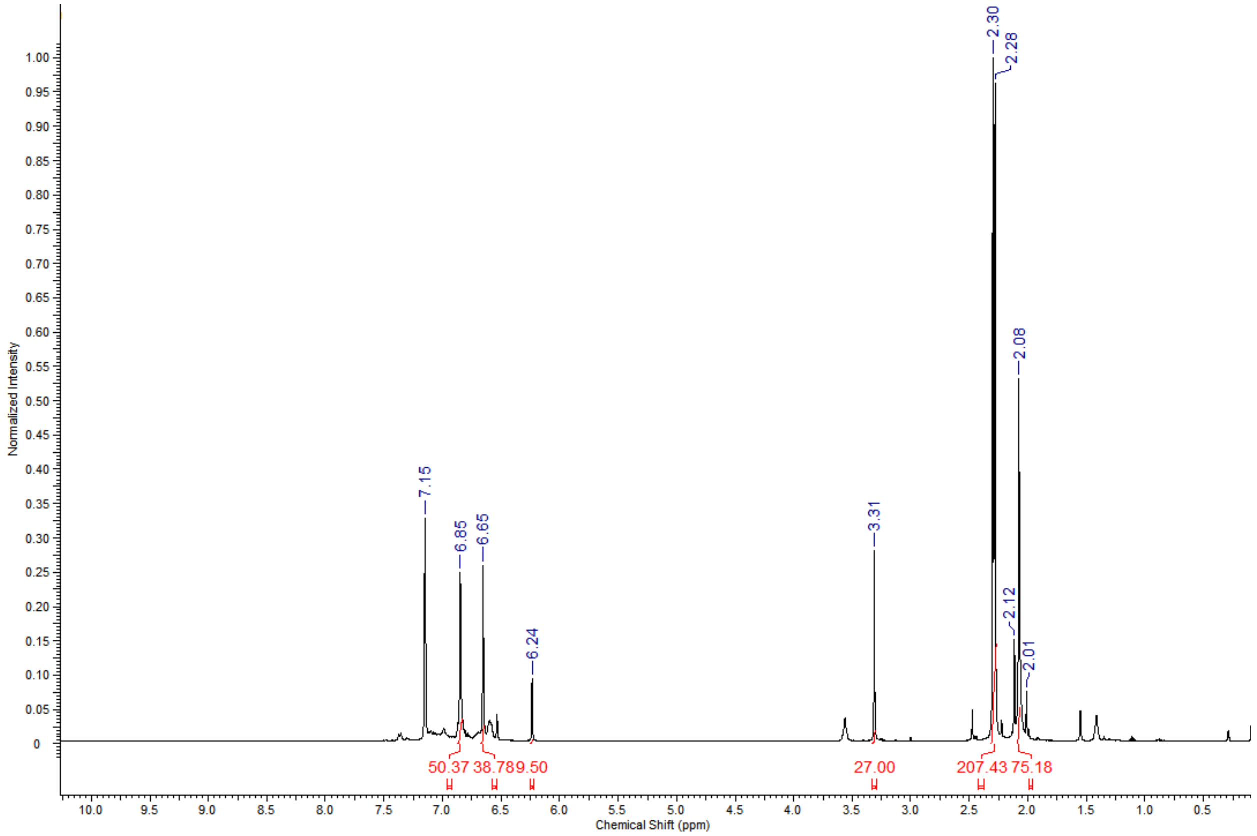

Figure S10. ^{1}H NMR of the catalytic reaction between mesityl azide, xylyl isocyanide, and **2**, demonstrating formation of respective carbodiimide.

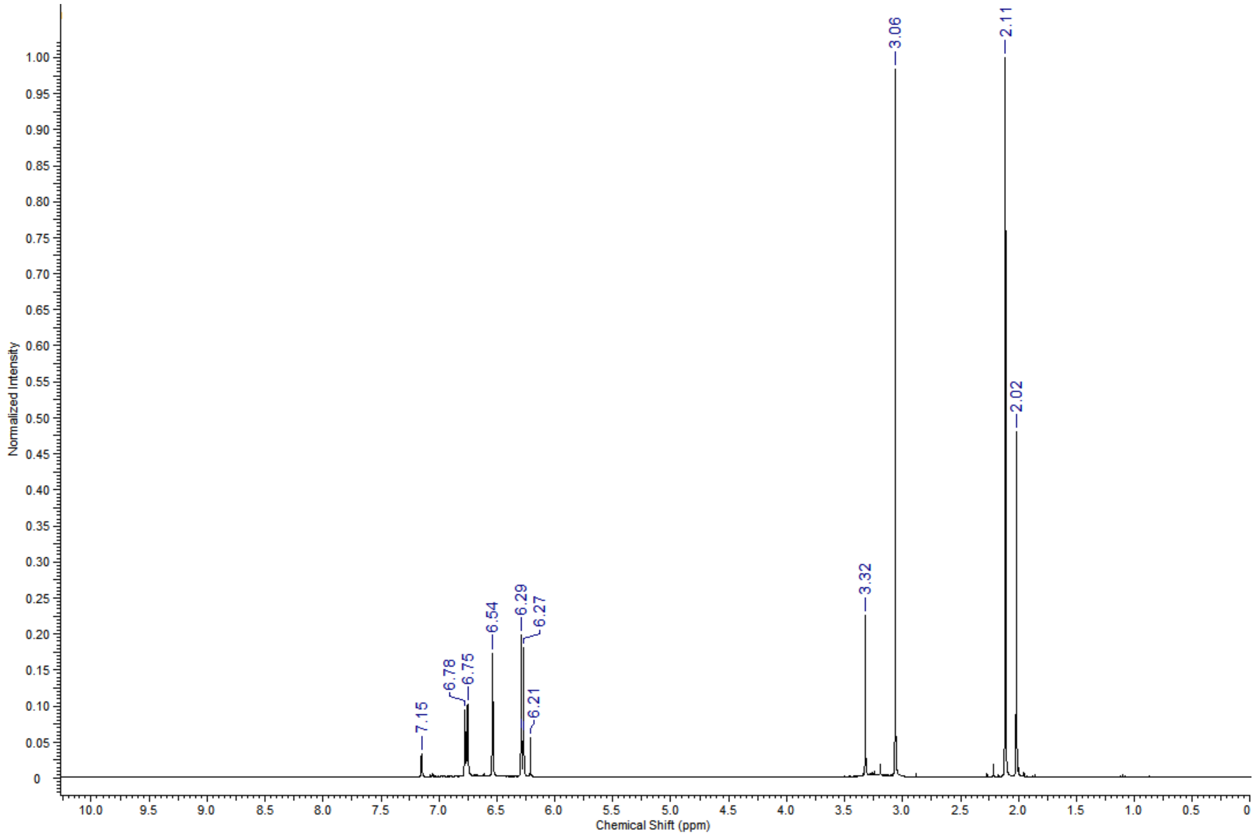

Figure S11. ^{1}H NMR of mesityl azide and 4-methoxyphenyl isocyanide prior to the addition of the catalyst (the resonances at 3.32 and 6.21 belong to the internal standard TMB).

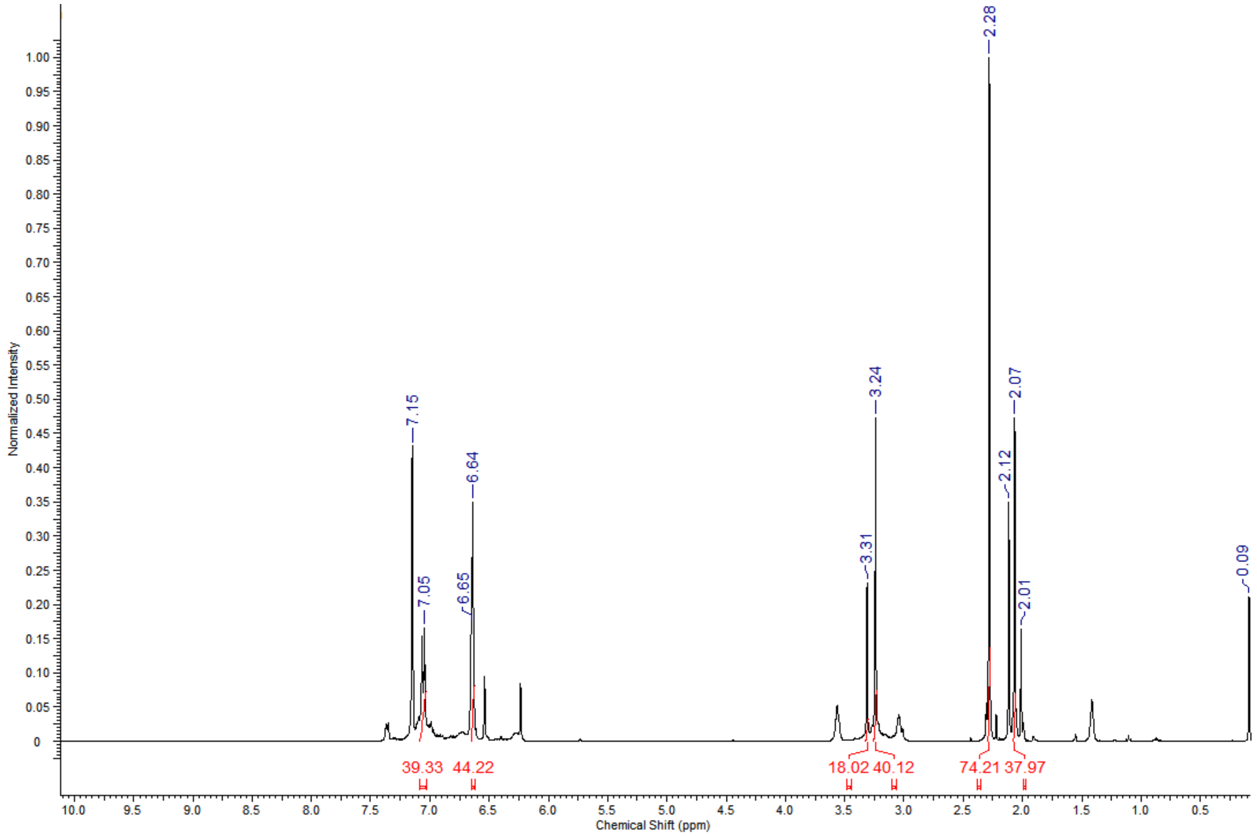

Figure S12. ^{1}H NMR of the catalytic reaction between mesityl azide, 4-methoxyphenyl isocyanide, and **2**, demonstrating formation of respective carbodiimide (see experimental for the reaction conditions).

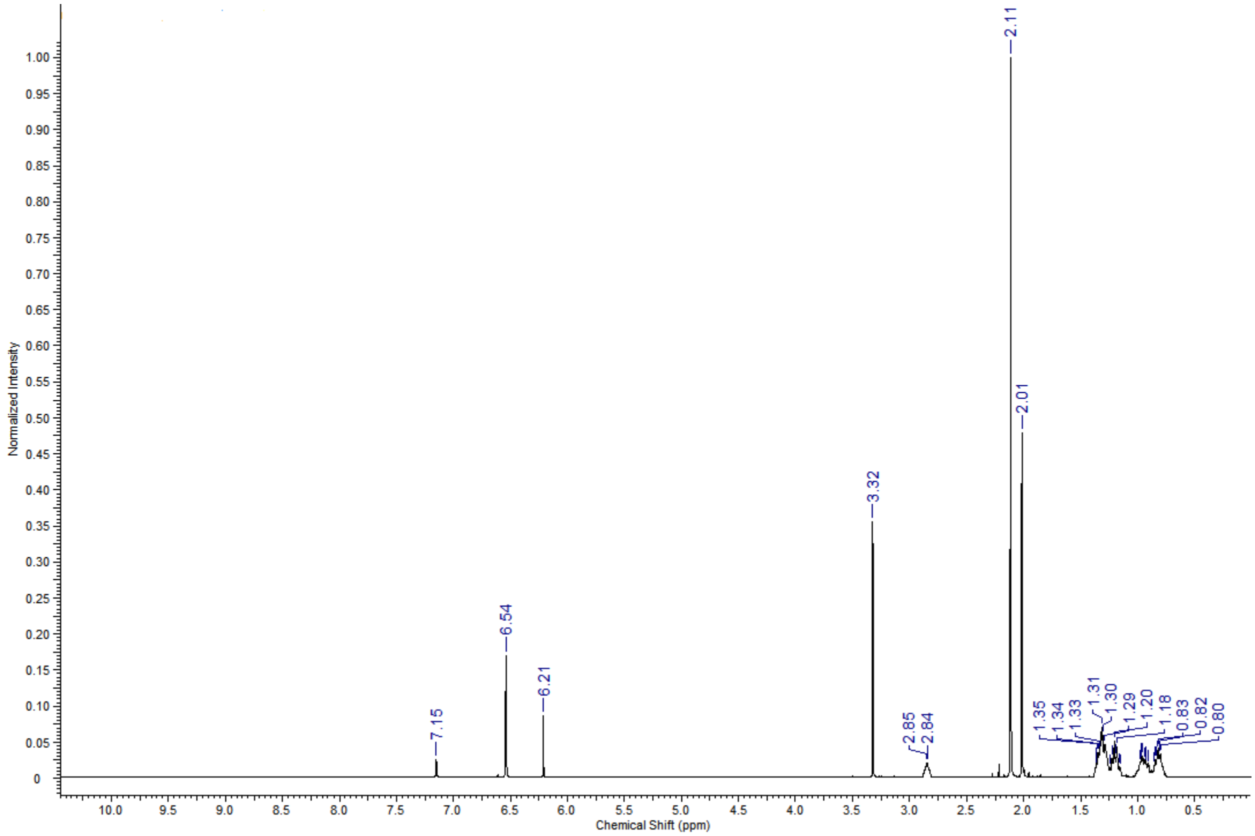

Figure S13. ^{1}H NMR of mesityl azide and cyclohexyl isocyanide prior to the addition of the catalyst (the resonances at 3.32 and 6.21 belong to the internal standard TMB).

.

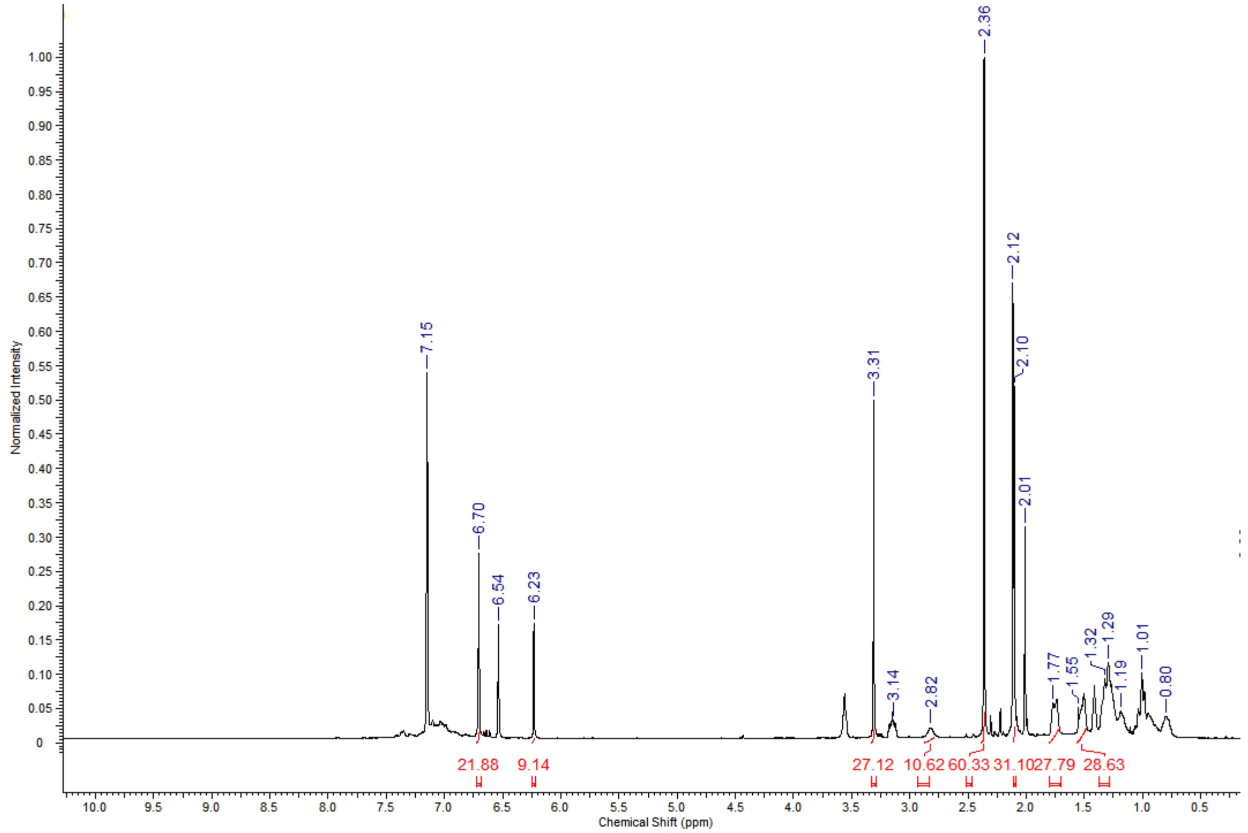

Figure S14. ^{1}H NMR of the reaction between mesityl azide, cyclohexyl isocyanide, and **2**, demonstrating formation of respective carbodiimide.

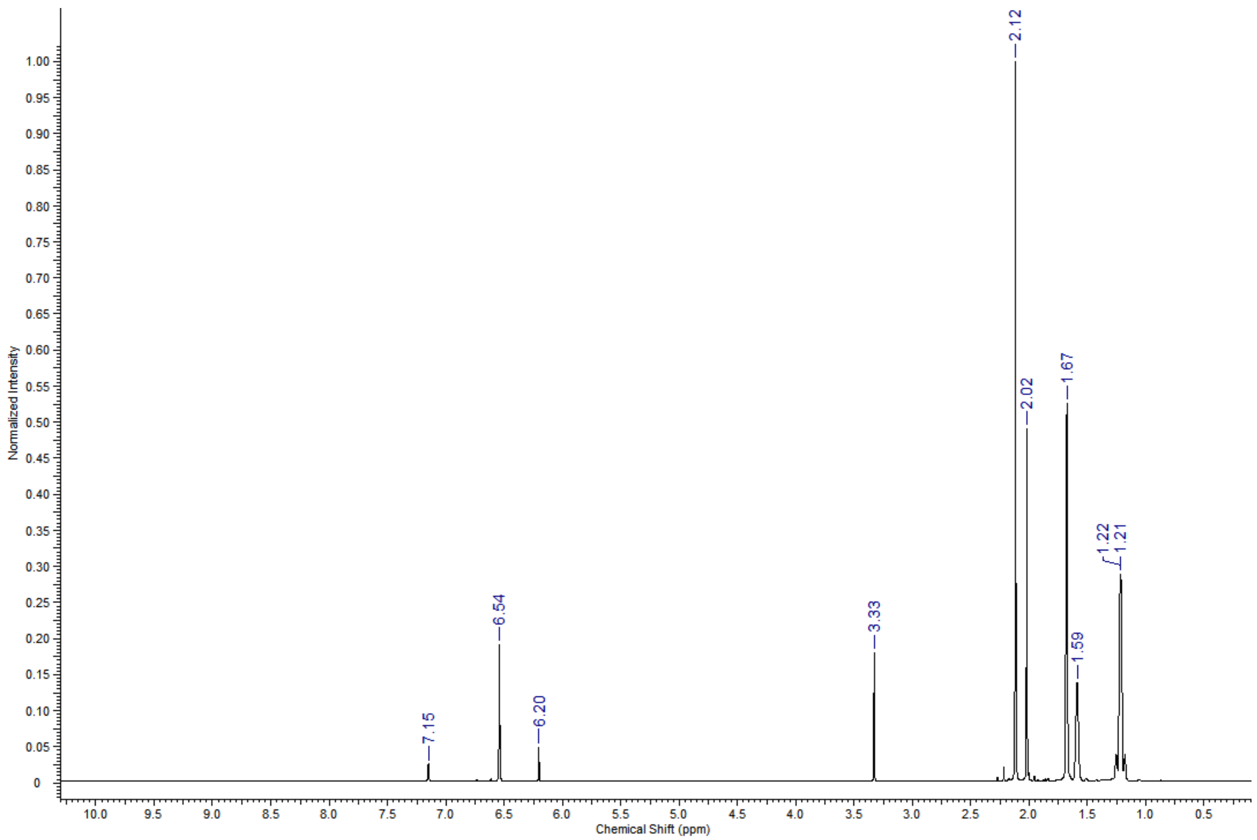

Figure S15. ^{1}H NMR of mesityl azide and 1-adamantyl isocyanide prior to the addition of the catalyst (the resonances at 3.33 and 6.20 belong to the internal standard TMB).

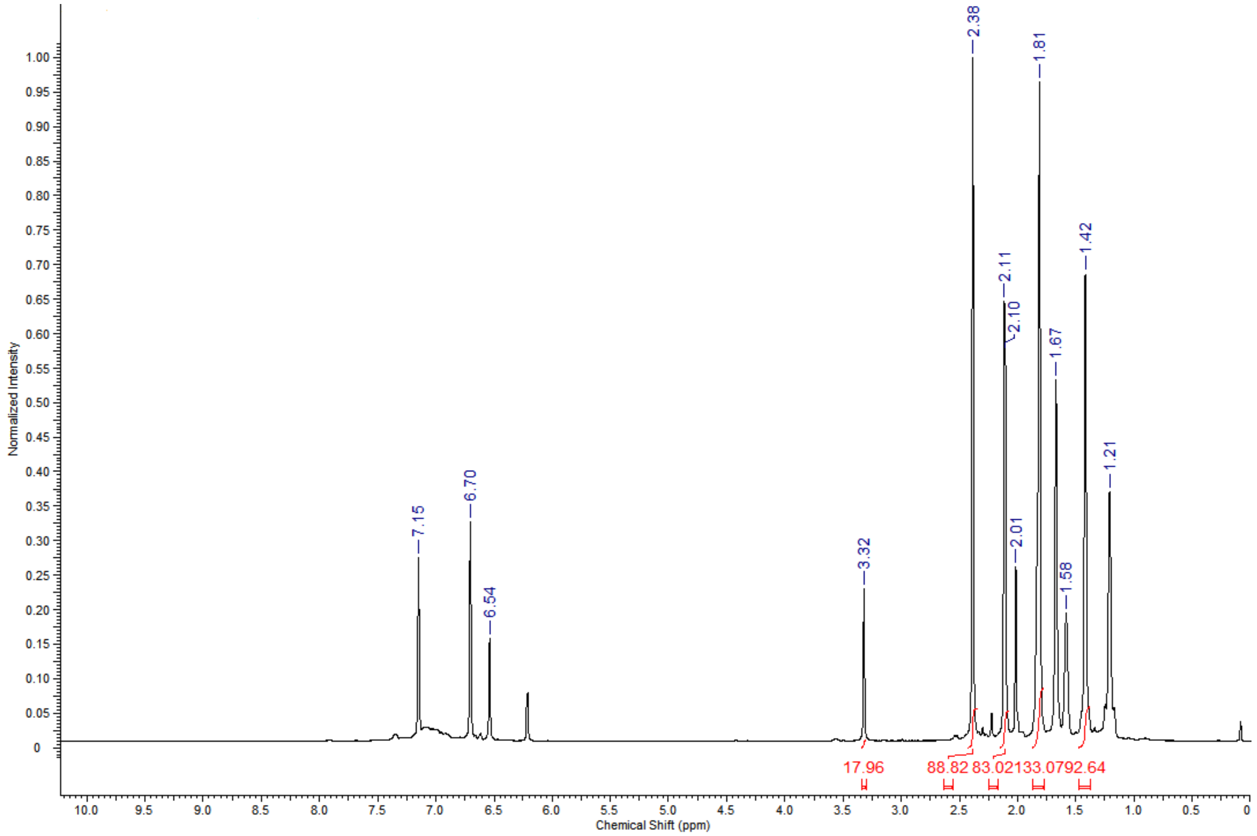

Figure S16. ^{1}H NMR of the reaction between mesityl azide, 1-adamantyl isocyanide, and **2**, showing formation of respective carbodiimde (carbodiimide peak at 2.10 ppm overlaps with unreacted mesityl azide peak at 2.11 ppm).

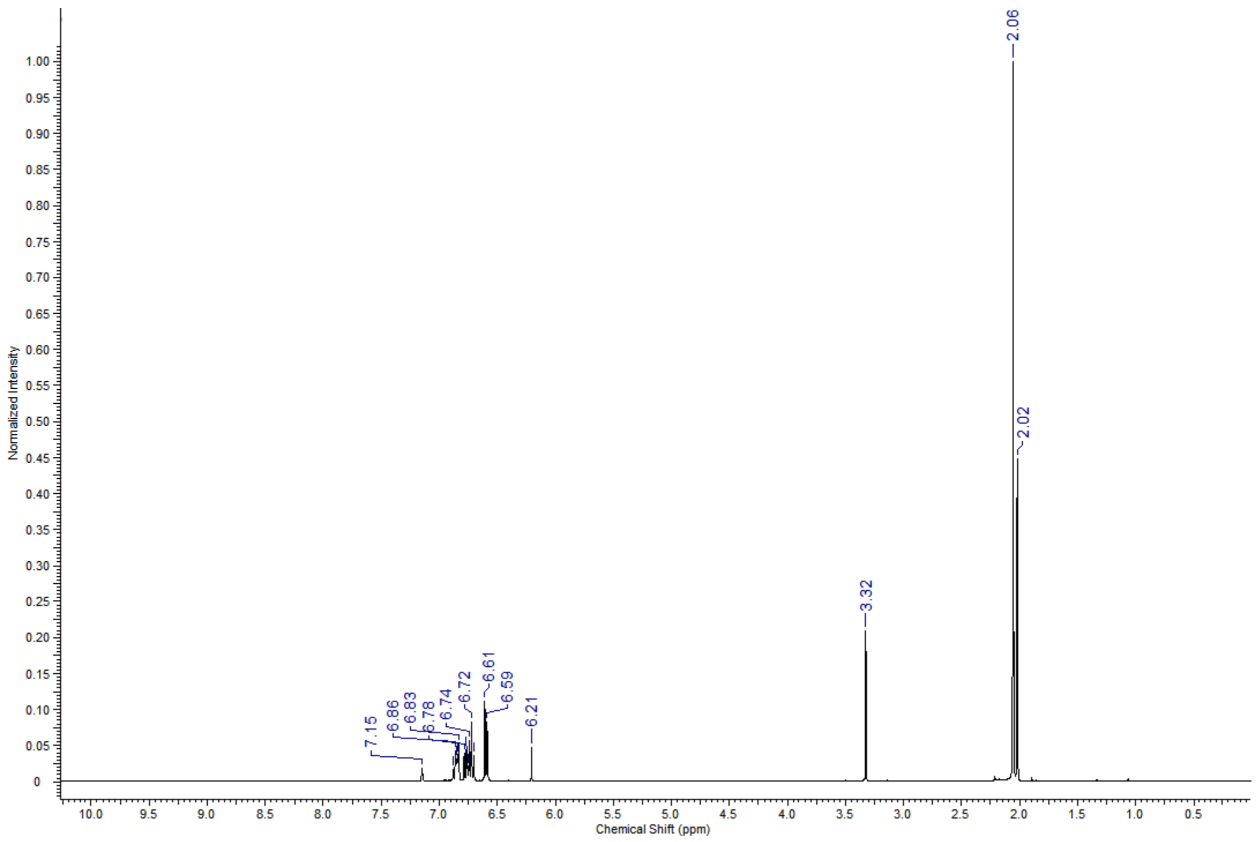

Figure S17. ^{1}H NMR of o-tolyl azide and xylyl isocyanide prior to the addition of the catalyst (the resonances at 3.32 and 6.21 belong to the internal standard TMB).

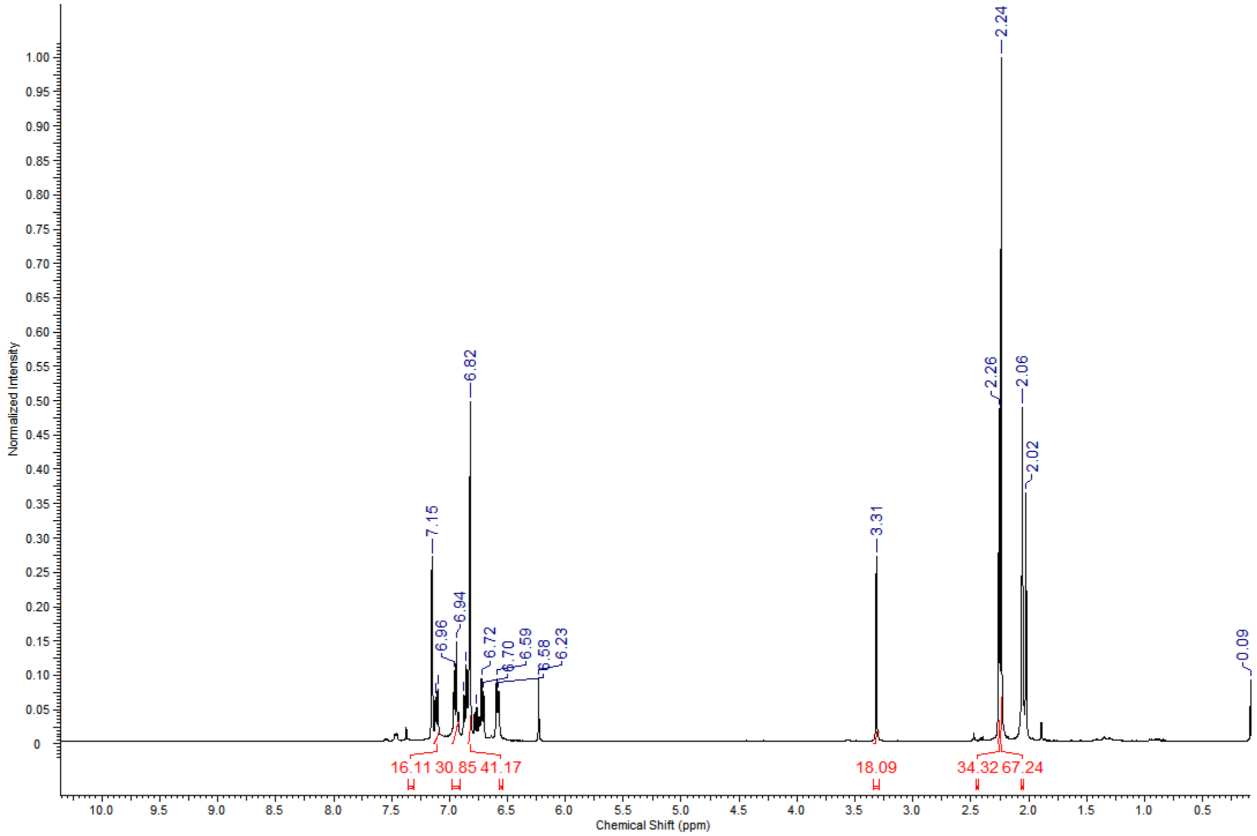

Figure S18. ^{1}H NMR of the reaction between o-tolyl azide, xylyl isocyanide, and **2**, showing formation of respective carbodiimide.

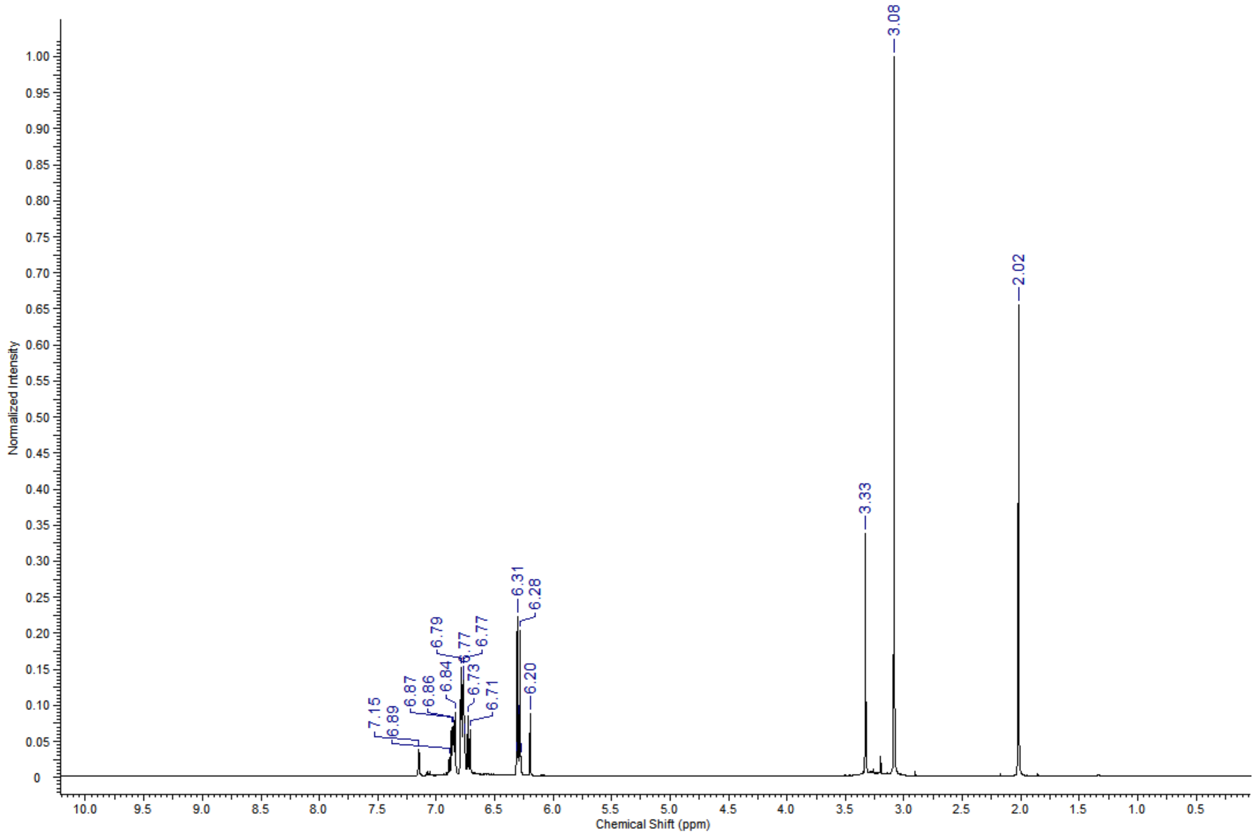

Figure S19. ^{1}H NMR of o-tolyl azide and 4-methoxyphenyl isocyanide prior to the addition of the catalyst (the resonances at 3.33 and 6.20 belong to the internal standard TMB).

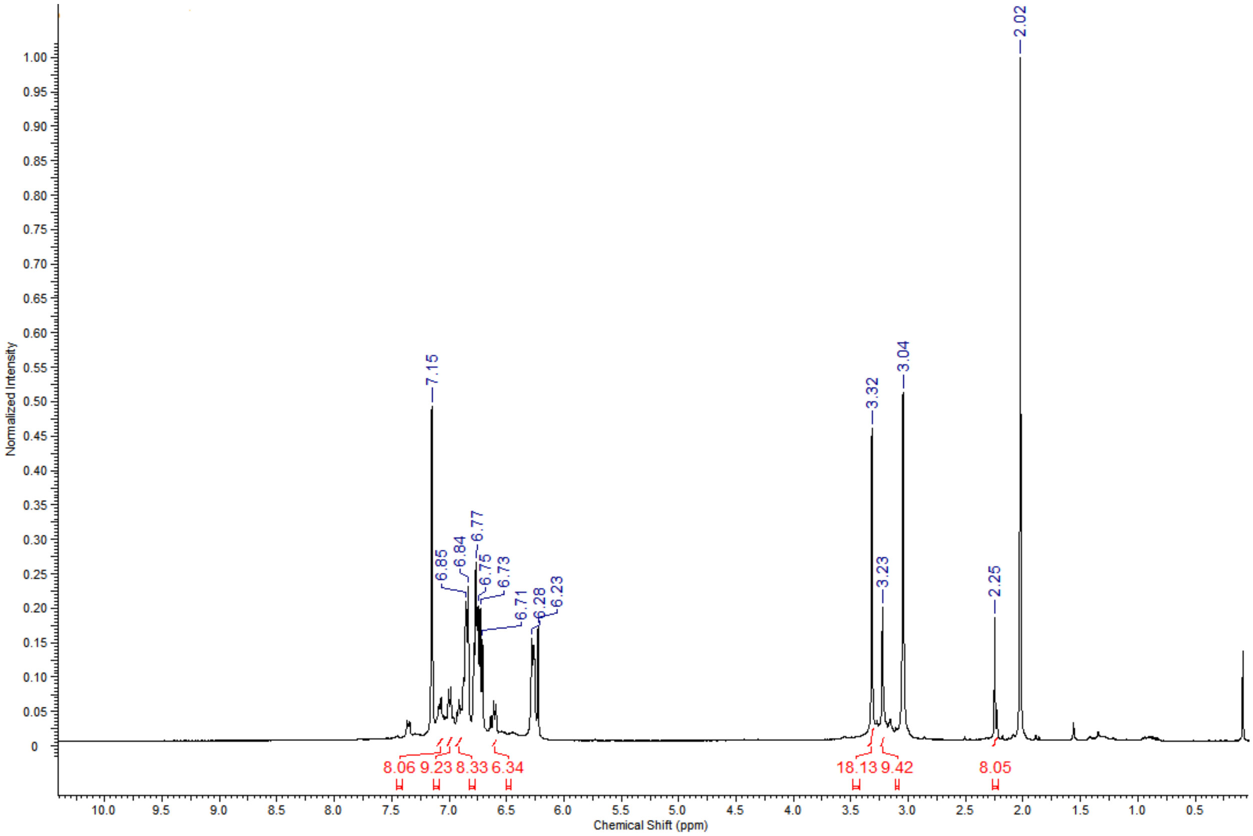

Figure S20. ^{1}H NMR of the reaction between o-tolyl azide, 4-methoxyphenyl isocyanide, and **2**, demonstrating formation of respective carbodiimide.

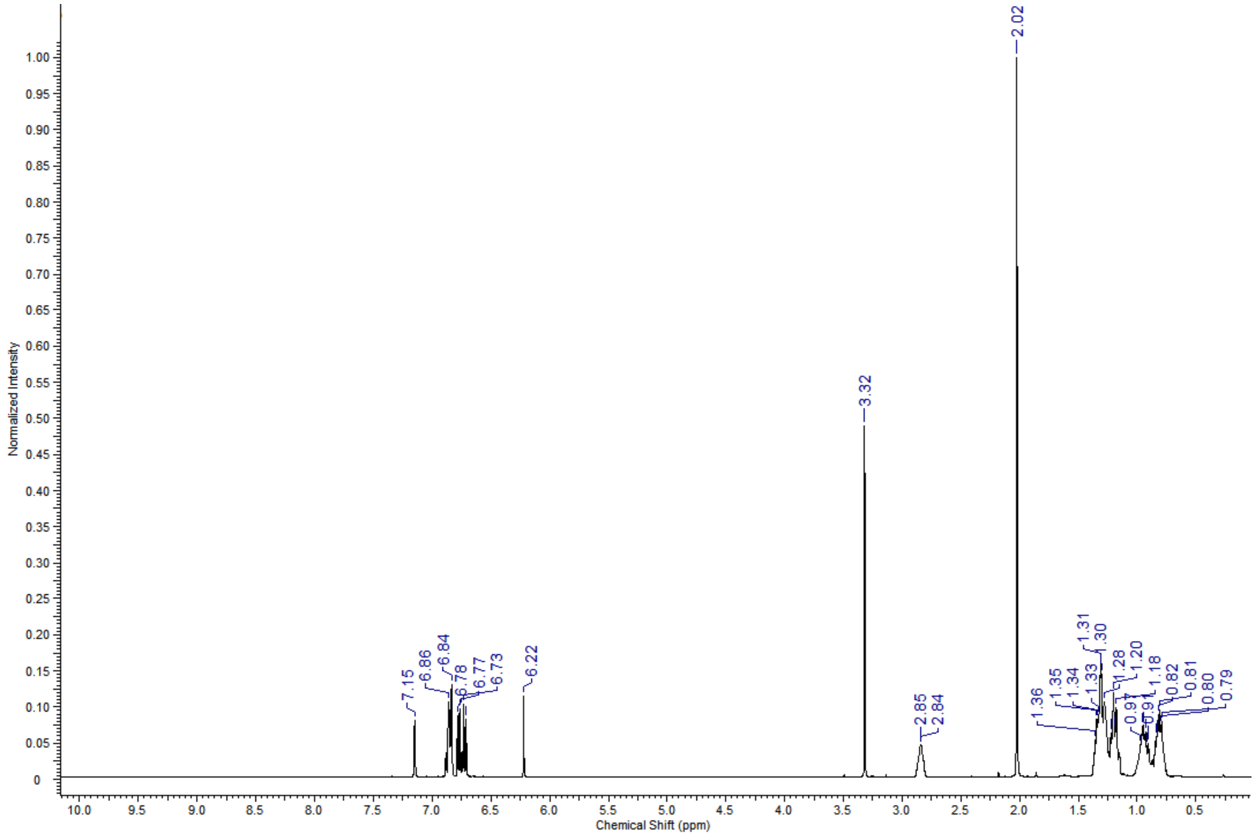

Figure S21. ^{1}H NMR of o-tolyl azide and cyclohexyl isocyanide prior to the addition of the catalyst (the resonances at 3.32 and 6.22 belong to the internal standard TMB).

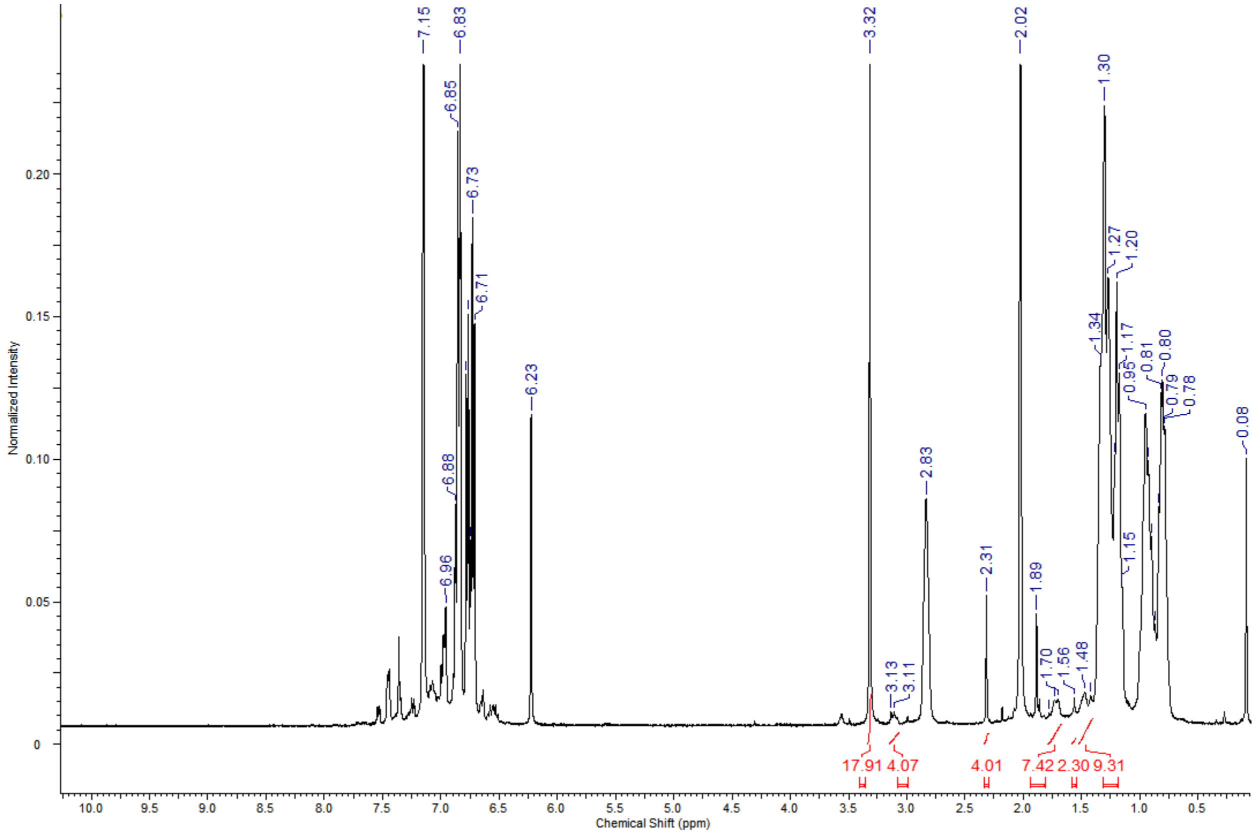

Figure S22. ^{1}H NMR of the reaction between o-tolyl azide, cyclohexyl isocyanide, and **2**, showing formation of respective carbodiimide.

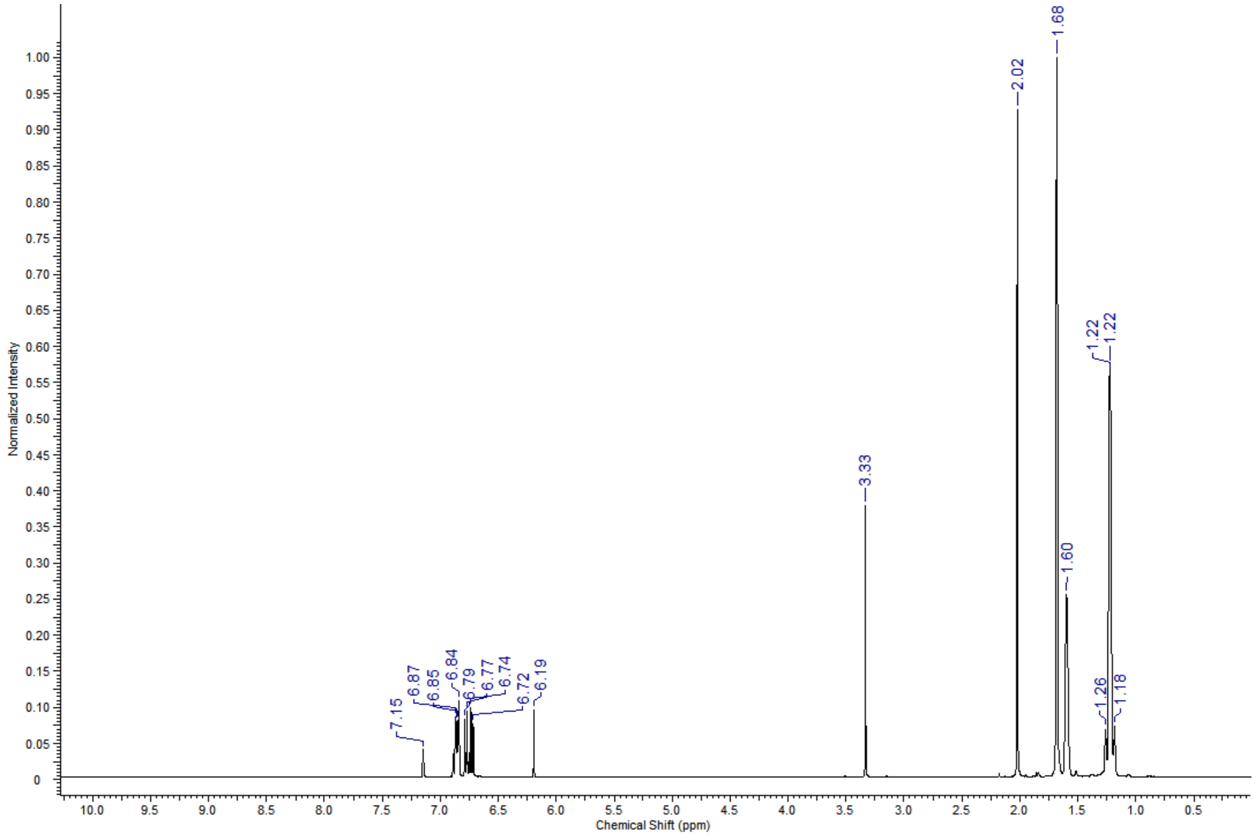

Figure S23. ^{1}H NMR of o-tolyl azide and adamantyl isocyanide prior to the addition of the catalyst (the resonances at 3.33 and 6.19 belong to the internal standard TMB).

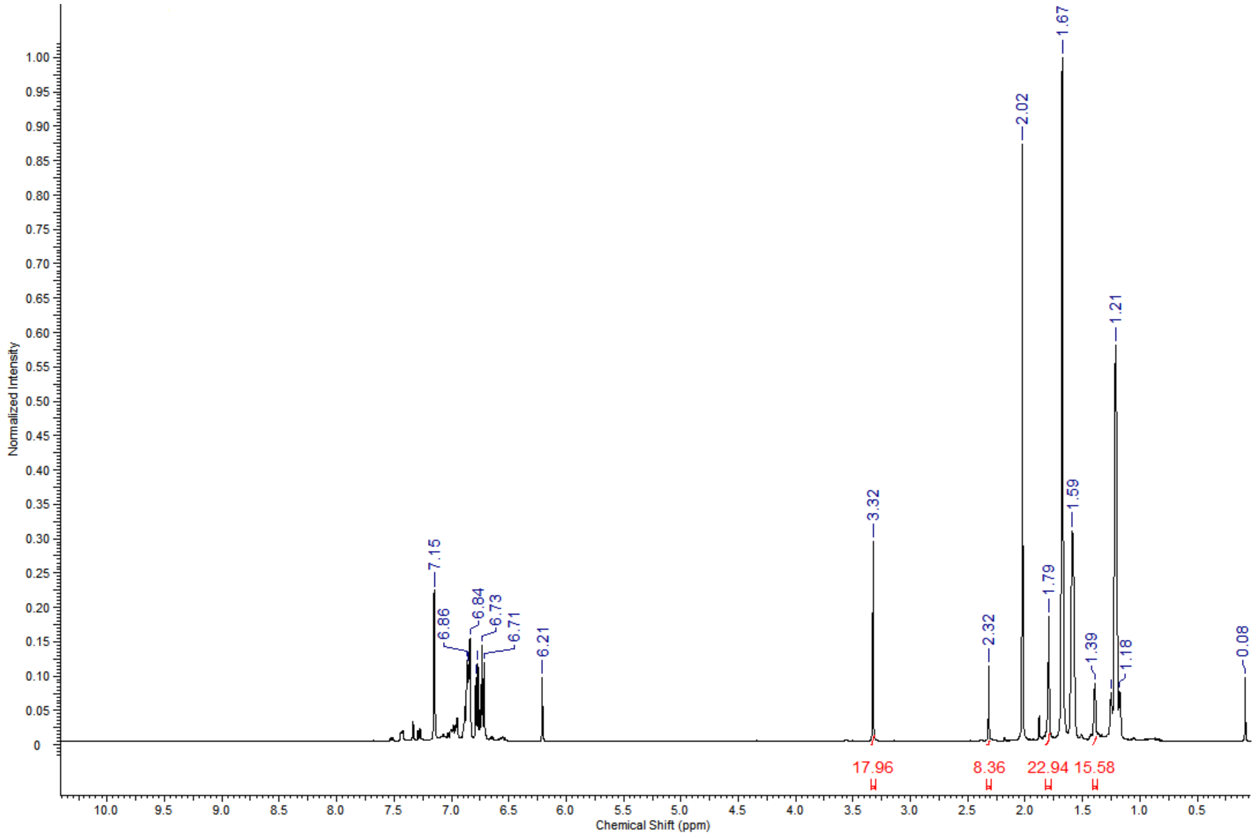

Figure S24. ^{1}H NMR of the reaction betweeb o-tolyl azide, adamantyl isocyanide, and **2**, showing formation of respective carbodiimide.

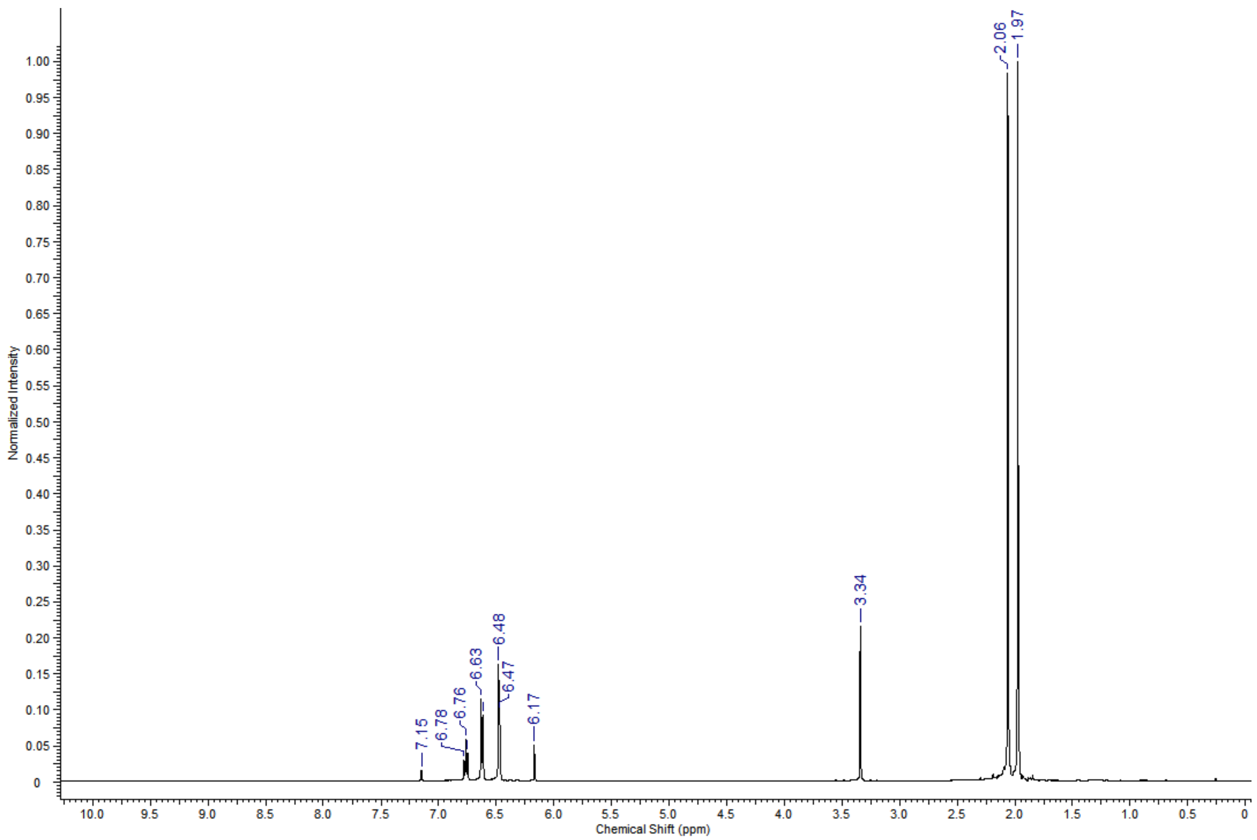

Figure S25. ^{1}H NMR of 3,5-dimethylphenyl azide and xylyl isocyanide prior to the addition of the catalyst (the resonances at 3.34 and 6.2217 belong to the internal standard TMB).

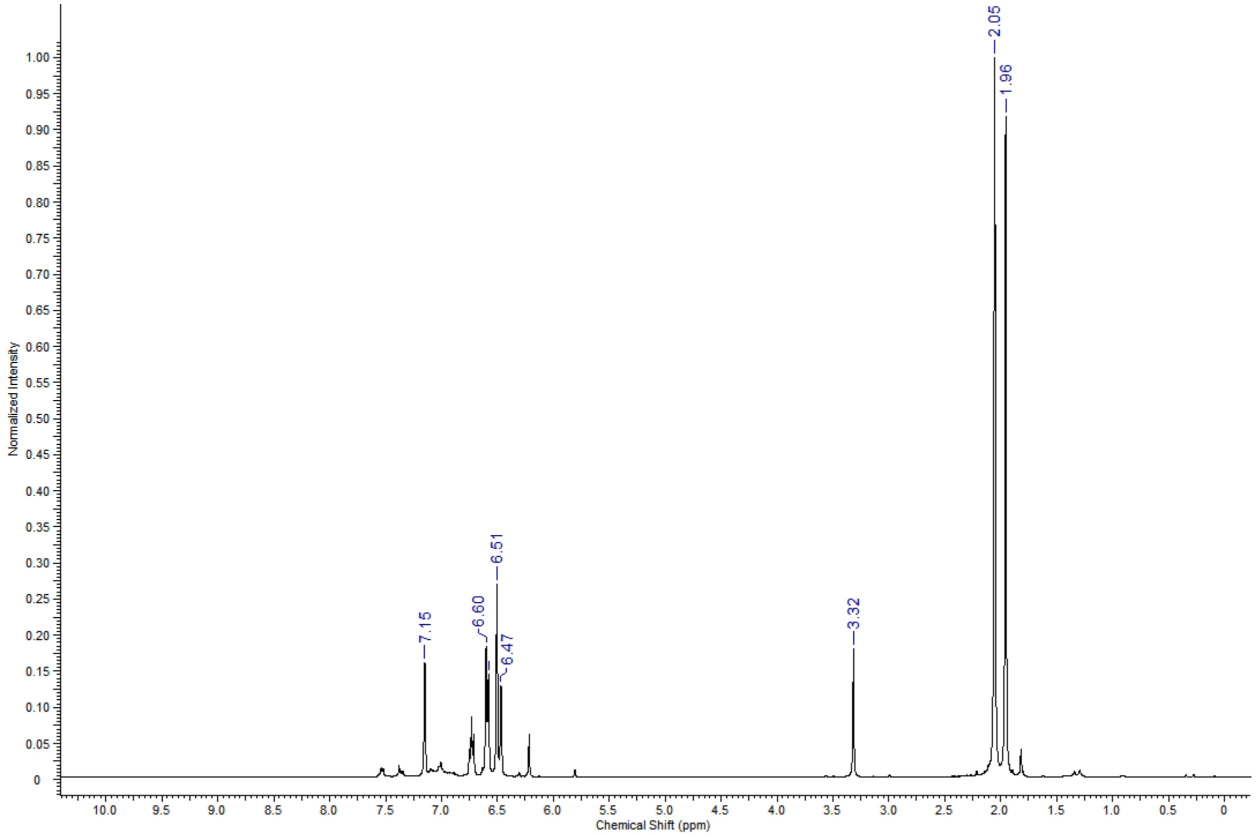

Figure S26. ^{1}H NMR of the reaction mixture of 3,5-dimethylphenyl azide, xylyl isocyanide, and 2 (see experimental for the reaction conditions), showing no formation of respective carbodiimide.

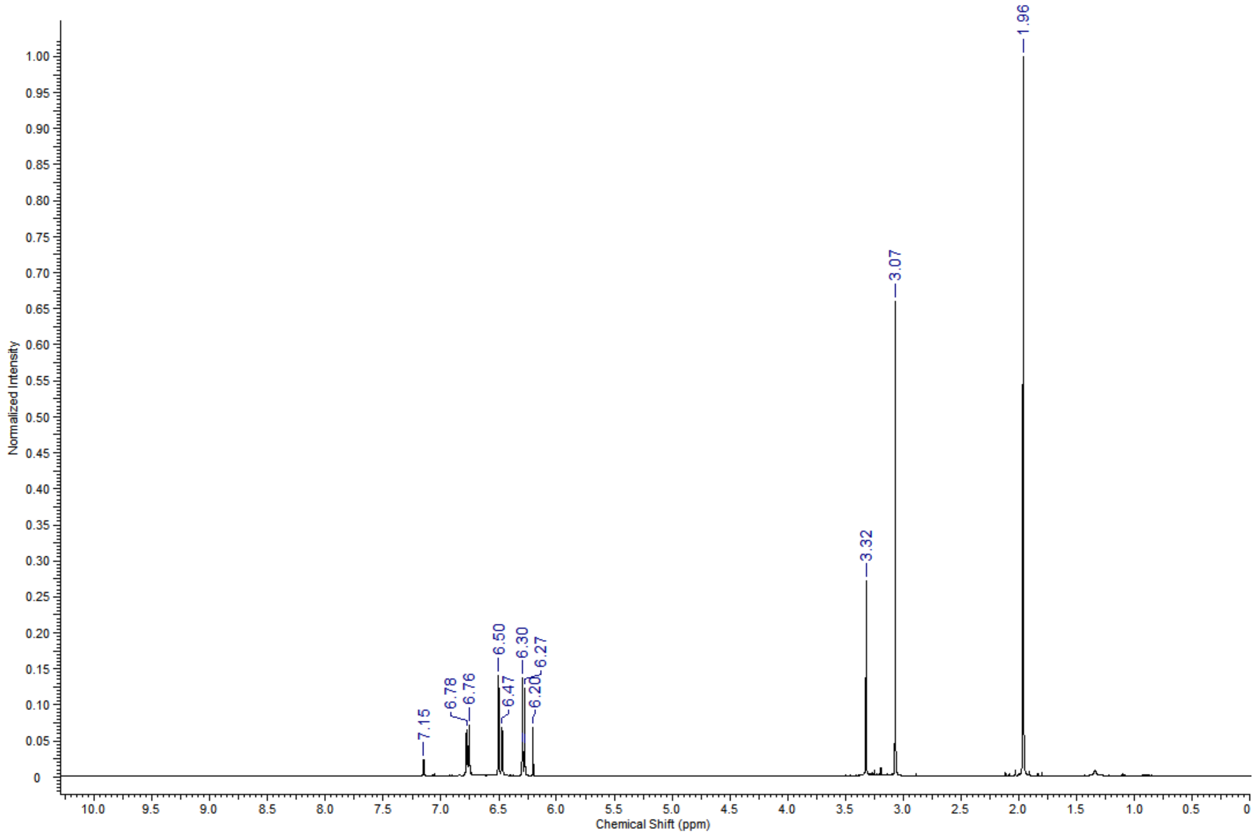

Figure S27. ^{1}H NMR of 3,5-dimethylphenyl azide and 4-methoxyphenyl isocyanide prior to the addition of the catalyst (the resonances at 3.32 and 6.20 belong to the internal standard TMB).

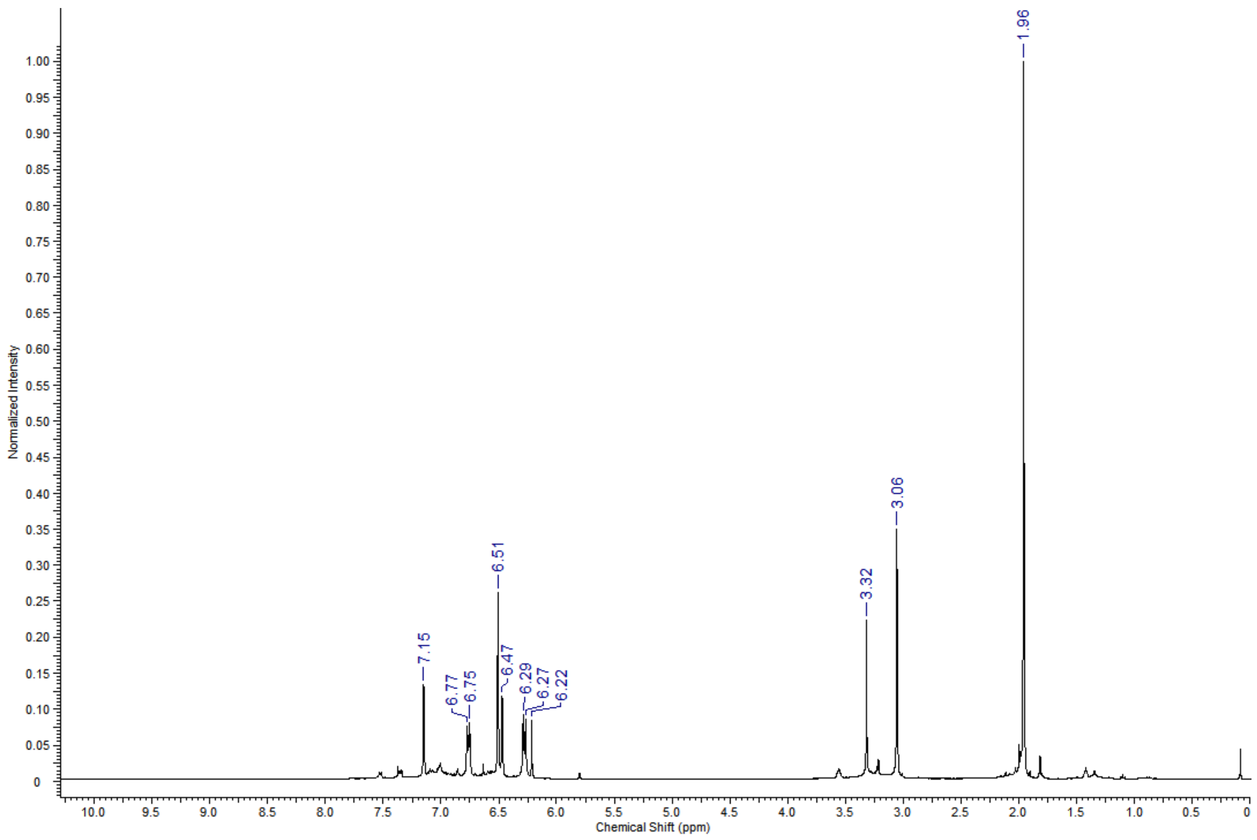

Figure S28. ^{1}H NMR of the reaction mixture containing 3,5-dimethyl phenyl azide, 4-methoxyphenyl isocyanide, and **2**, showing no formation of respective carbodiimide.

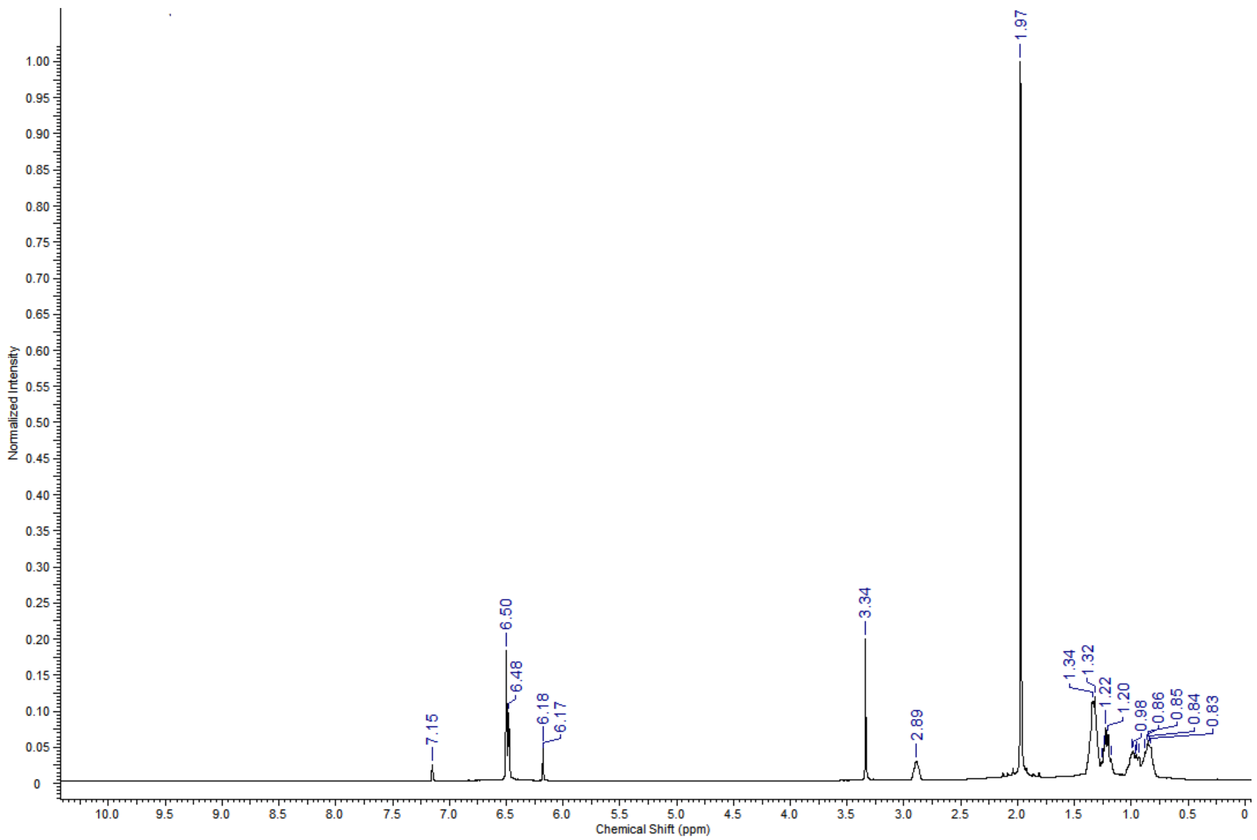

Figure S29. ^{1}H NMR of 3,5-dimethylphenyl azide and cyclohexyl isocyanide prior to the addition of the catalyst (the resonances at 3.34 and 6.18 belong to the internal standard TMB).

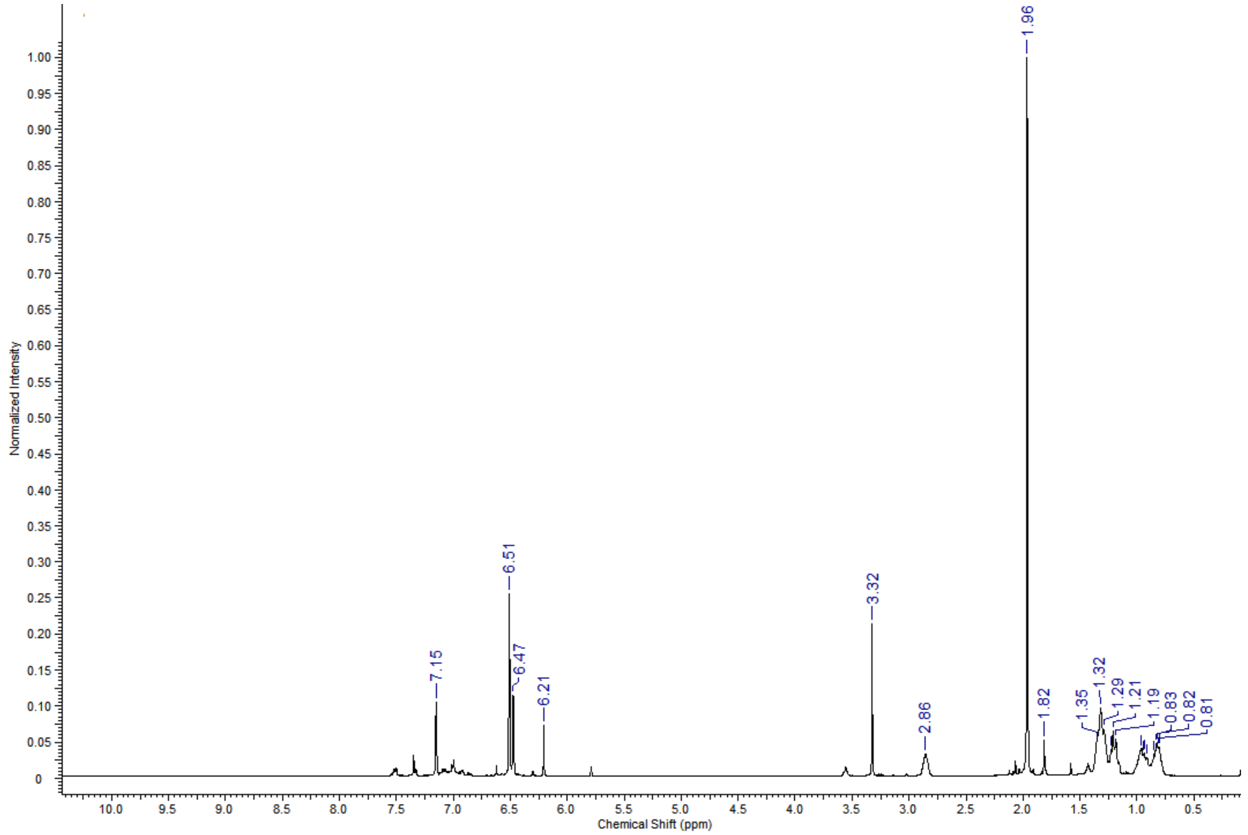

Figure S30. ^{1}H NMR of the reaction mixture containing 3,5-dimethylphenyl azide, cyclohexyl isocyanide, and **2**, showing no formation of respective carbodiimide (see experimental for the reaction details).

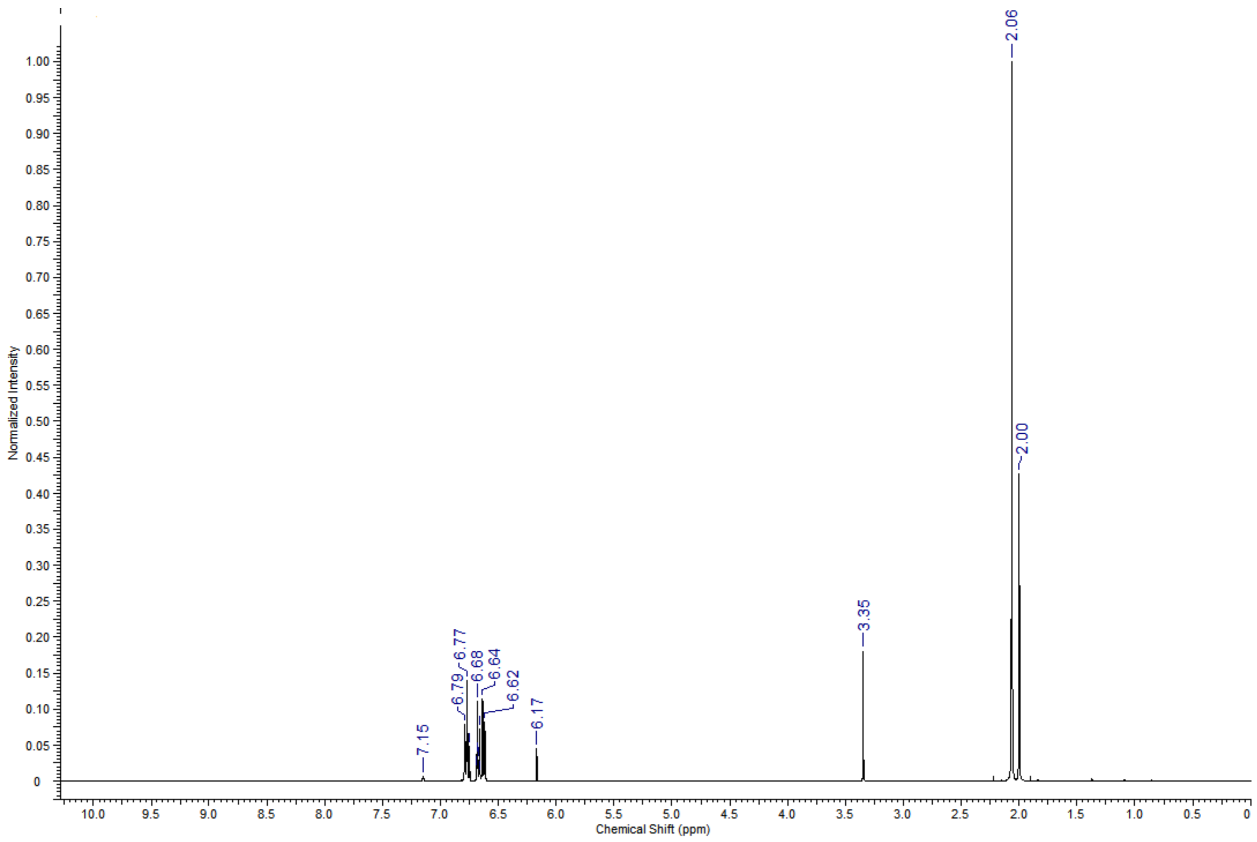

Figure S31. ^{1}H NMR of p-tolyl azide and 2,6-dimethylphenyl isocyanide prior to the addition of the catalyst (the resonances at 3.35 and 6.17 belong to the internal standard TMB).

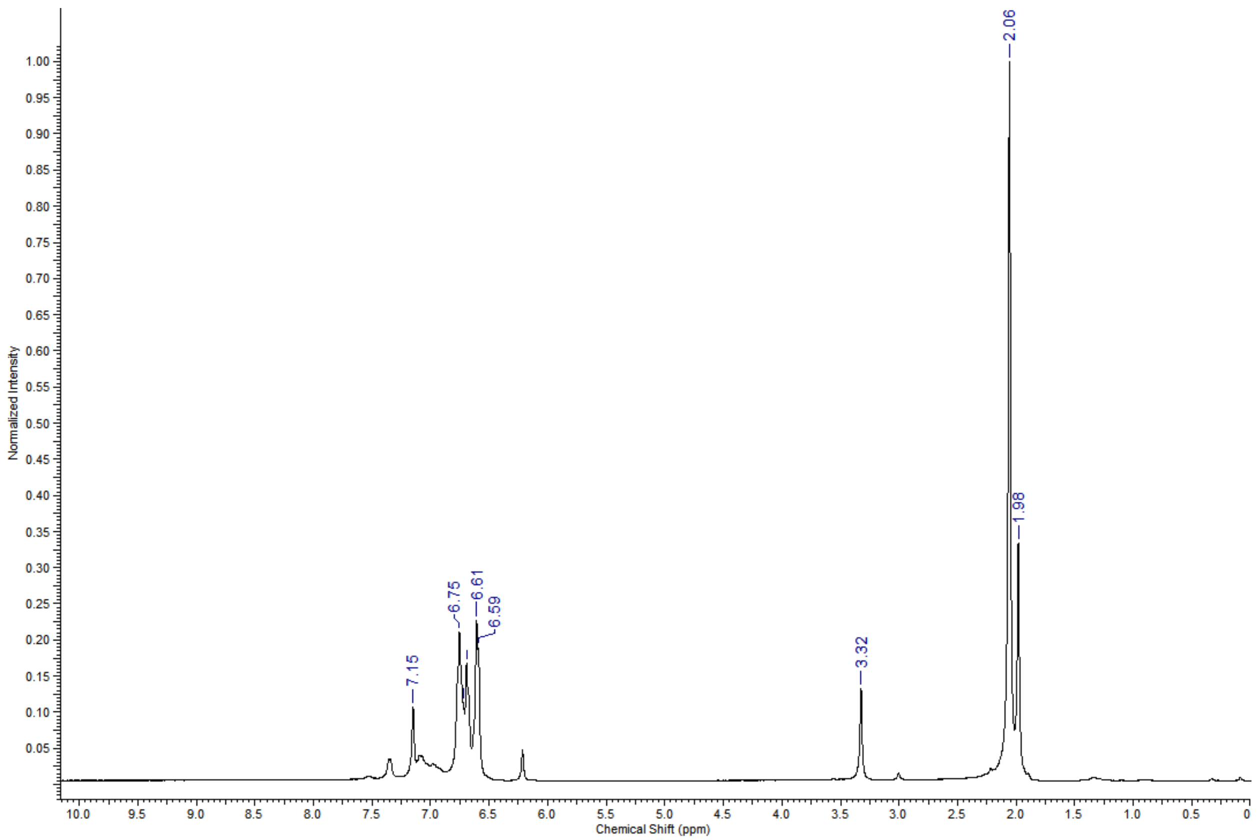

Figure S32. ^{1}H NMR of the reaction mixture containing p-tolyl azide, 2,6-dimethylphenyl isocyanide, and **2**, showing no formation of respective carbodiimide.

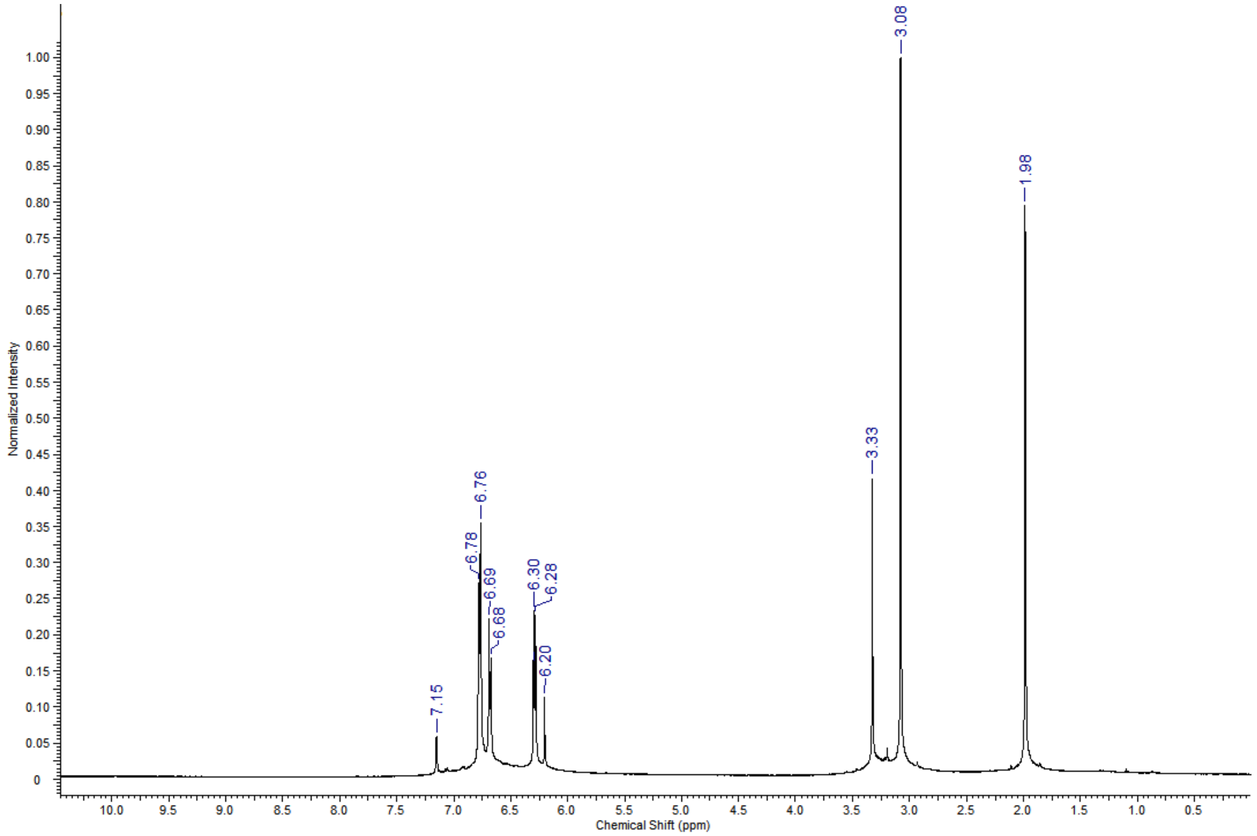

Figure S33. ^{1}H NMR of p-tolyl azide and 4-methoxyphenyl isocyanide prior to the addition of the catalyst (the resonances at 3.33 and 6.20 belong to the internal standard TMB).

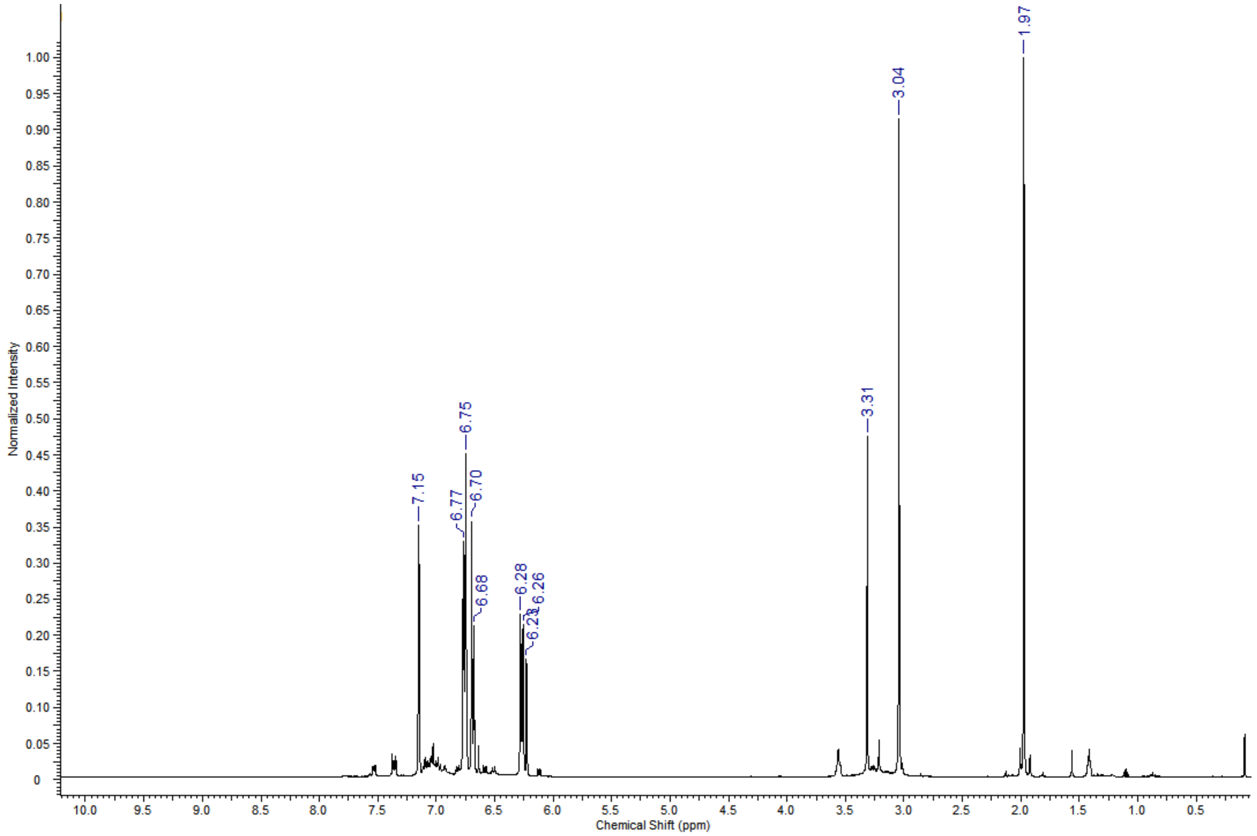

Figure S34. ^{1}H NMR of the reaction mixture containing p-tolyl azide, 4-methoxyphenyl isocyanide, and **2**, showing no formation of respective carbodiimide.

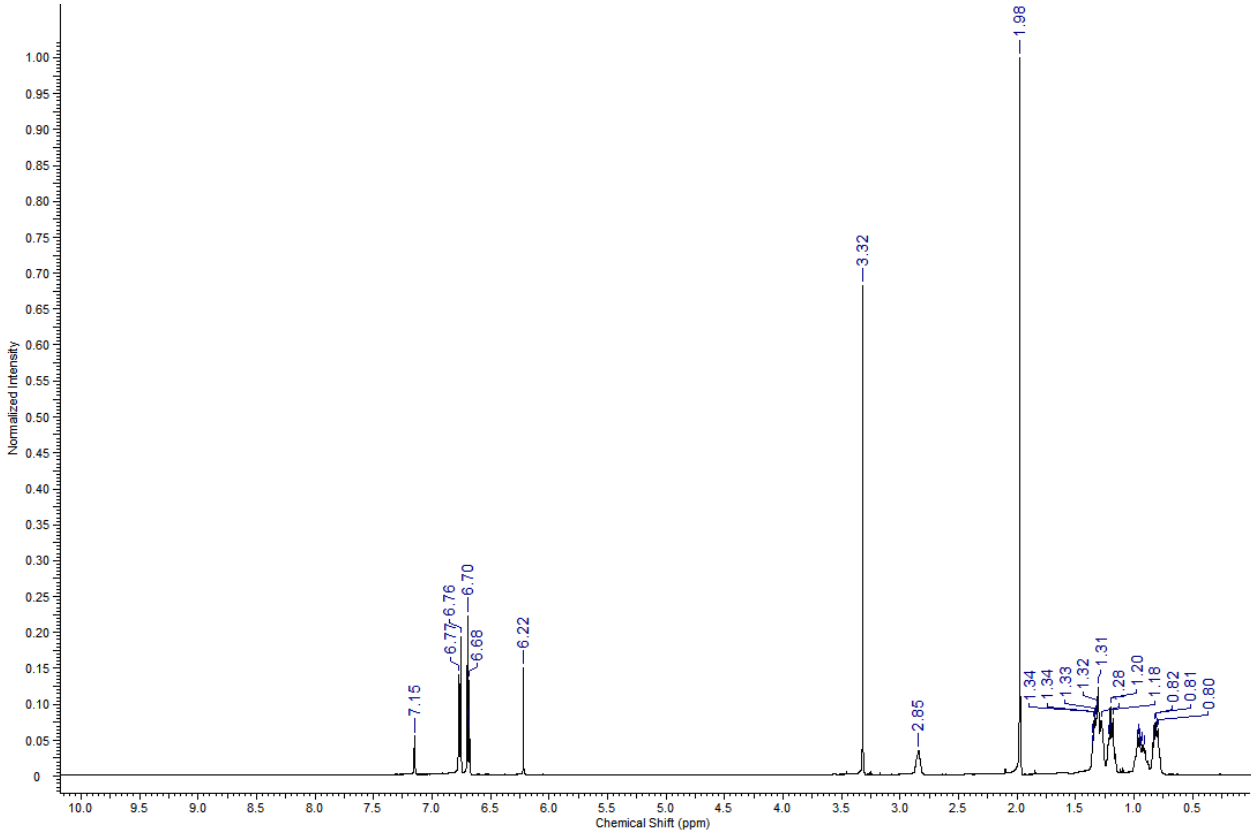

Figure S35. ^{1}H NMR of p-tolyl azide and cyclohexyl isocyanide prior to the addition of the catalyst (the resonances at 3.32 and 6.22 belong to the internal standard TMB).

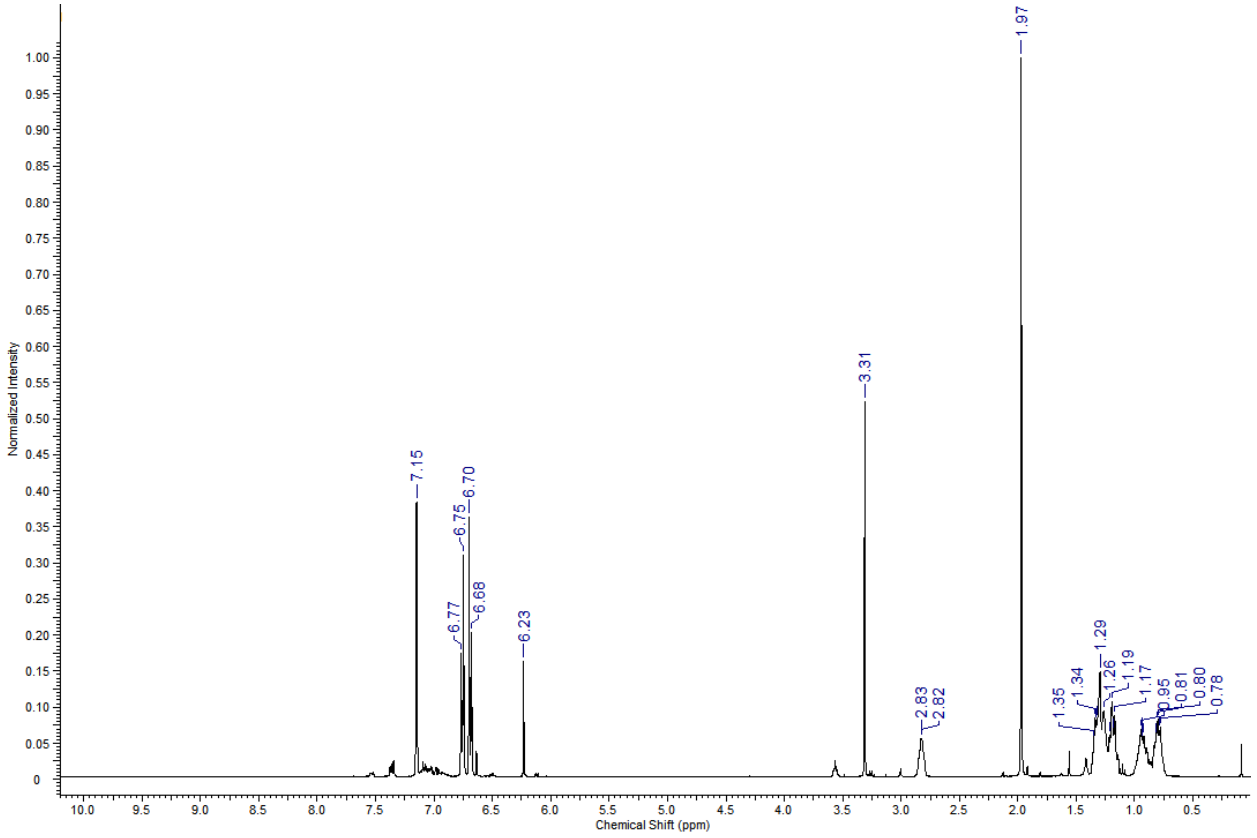

Figure S36. ^{1}H NMR of the reaction mixture containing p-tolyl azide, cyclohexyl isocyanide, and **2**, showing no formation of respective carbodiimide.

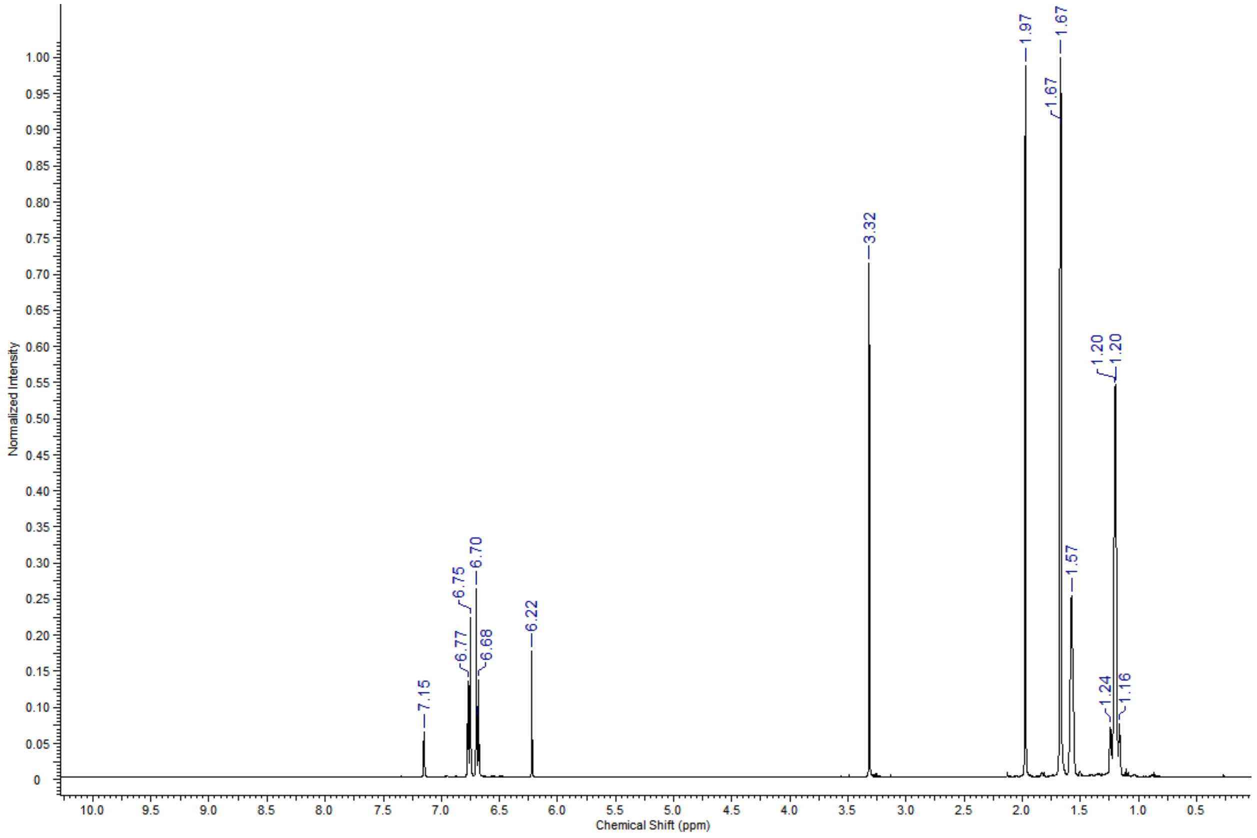

Figure S37. ^{1}H NMR of p-tolyl azide and adamantyl isocyanide prior to the addition of the catalyst (the resonances at 3.32 and 6.22 ppm belong to the internal standard TMB).

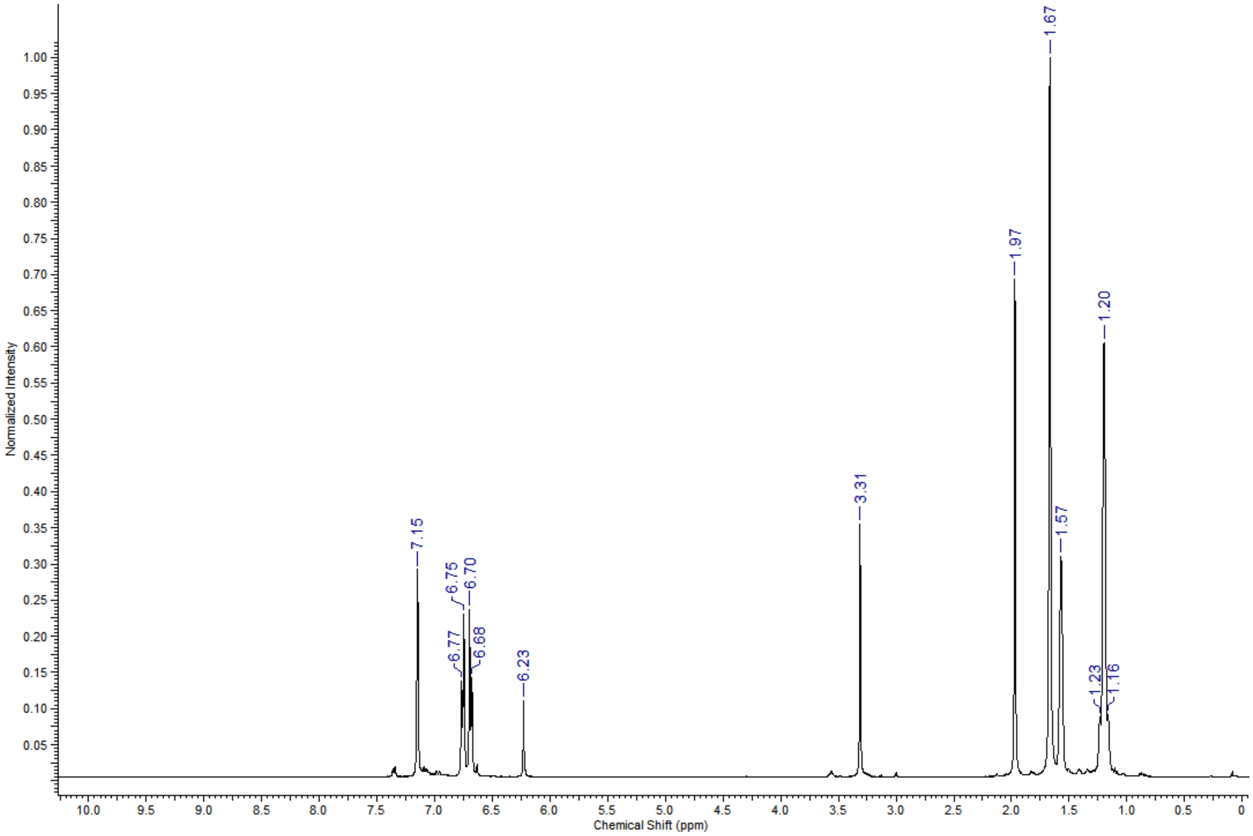

Figure S38. ^{1}H NMR of the reaction mixture containing p-tolyl azide, adamantyl isocyanide and **2**, showing no formation of respective carbodiimide.

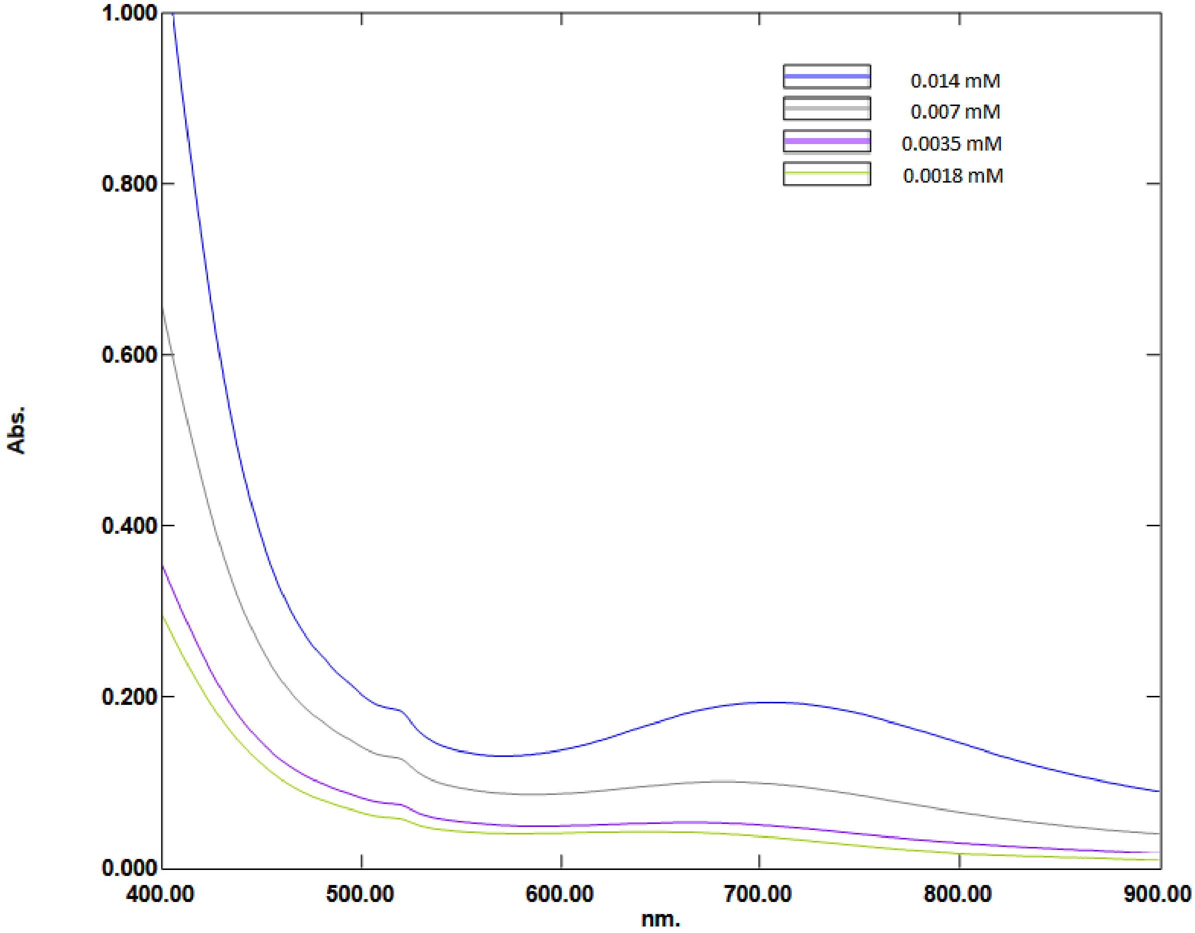

Figure S39. UV-Vis spectrum of $Cr_2([OO]^{Ph})_2$

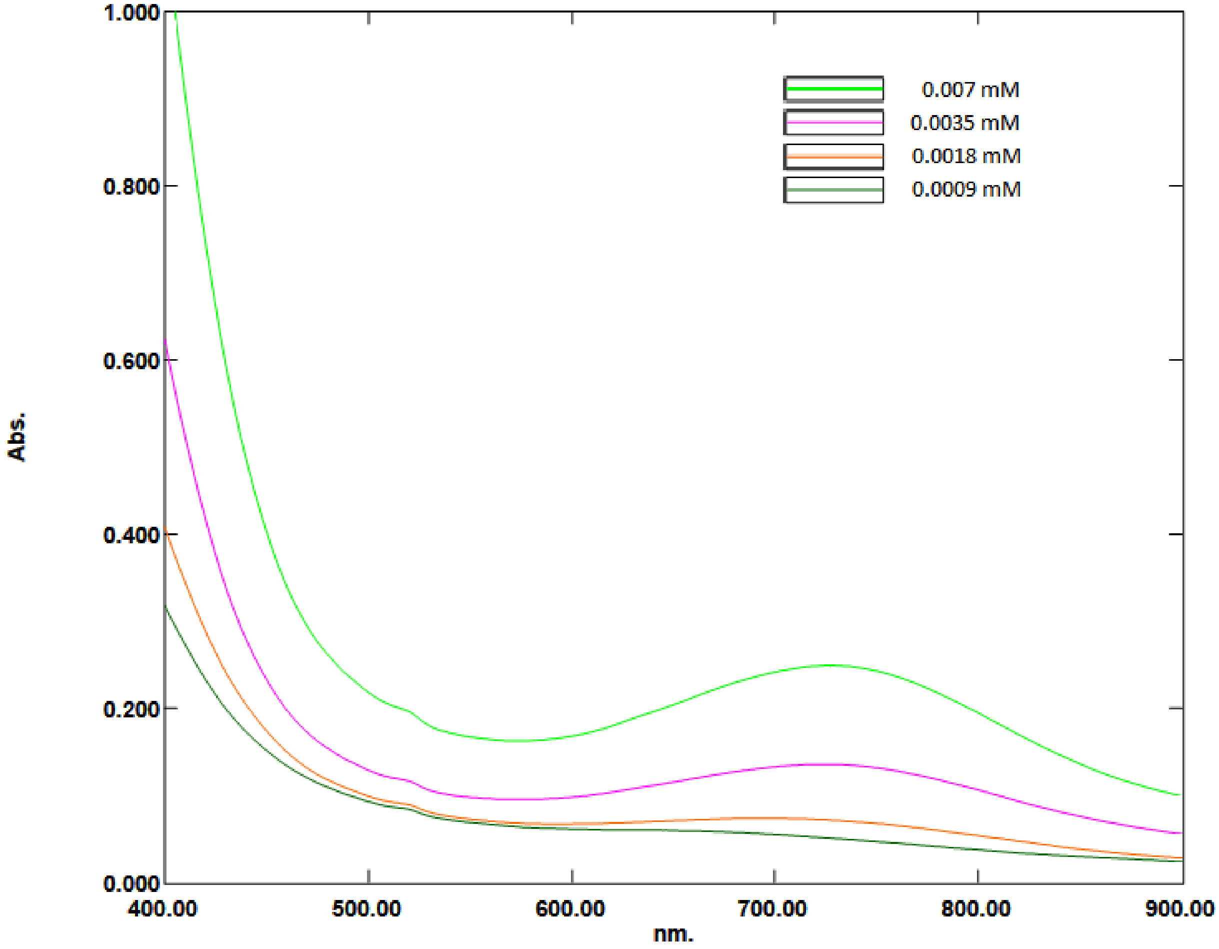

Figure S40. UV-Vis spectrum of Cr[OO]^{Ph}(THF)$_2$

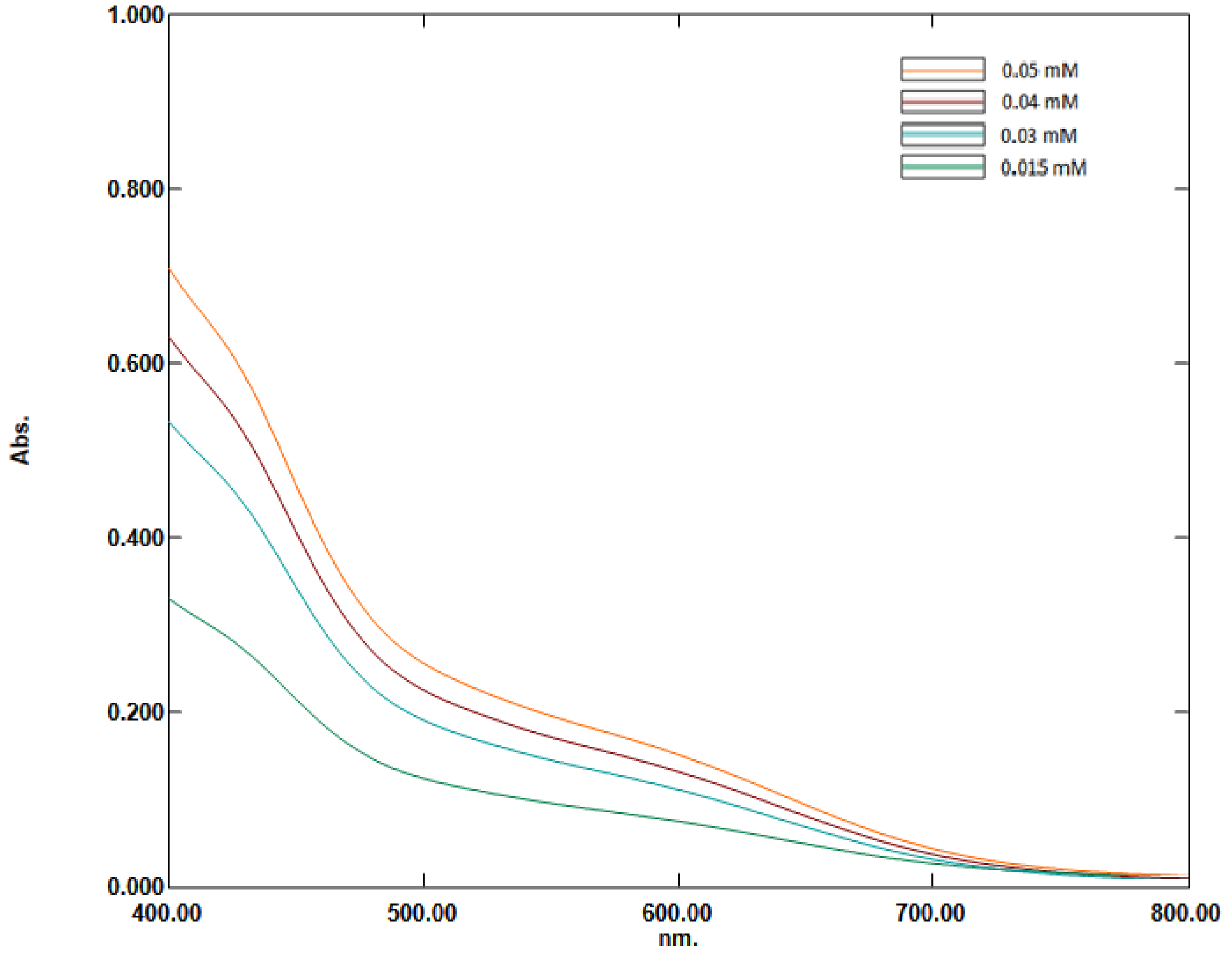

Figure S41. UV-Vis spectrum of Cr[OO]^{Ph}(N(4-CH_3Ph))$_2$.

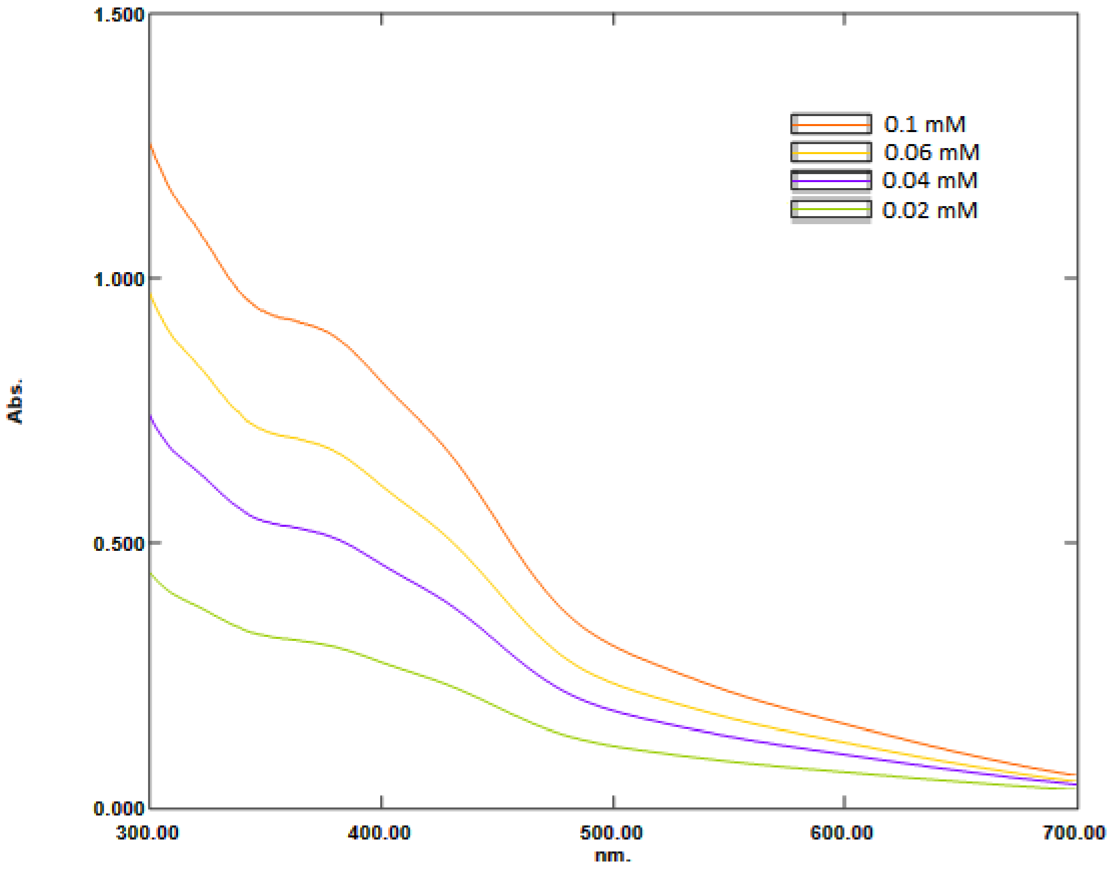

Figure S42. UV-Vis spectrum of $Cr[OO]^{Ph}(N(4\text{-}CH_3OPh))_2$.

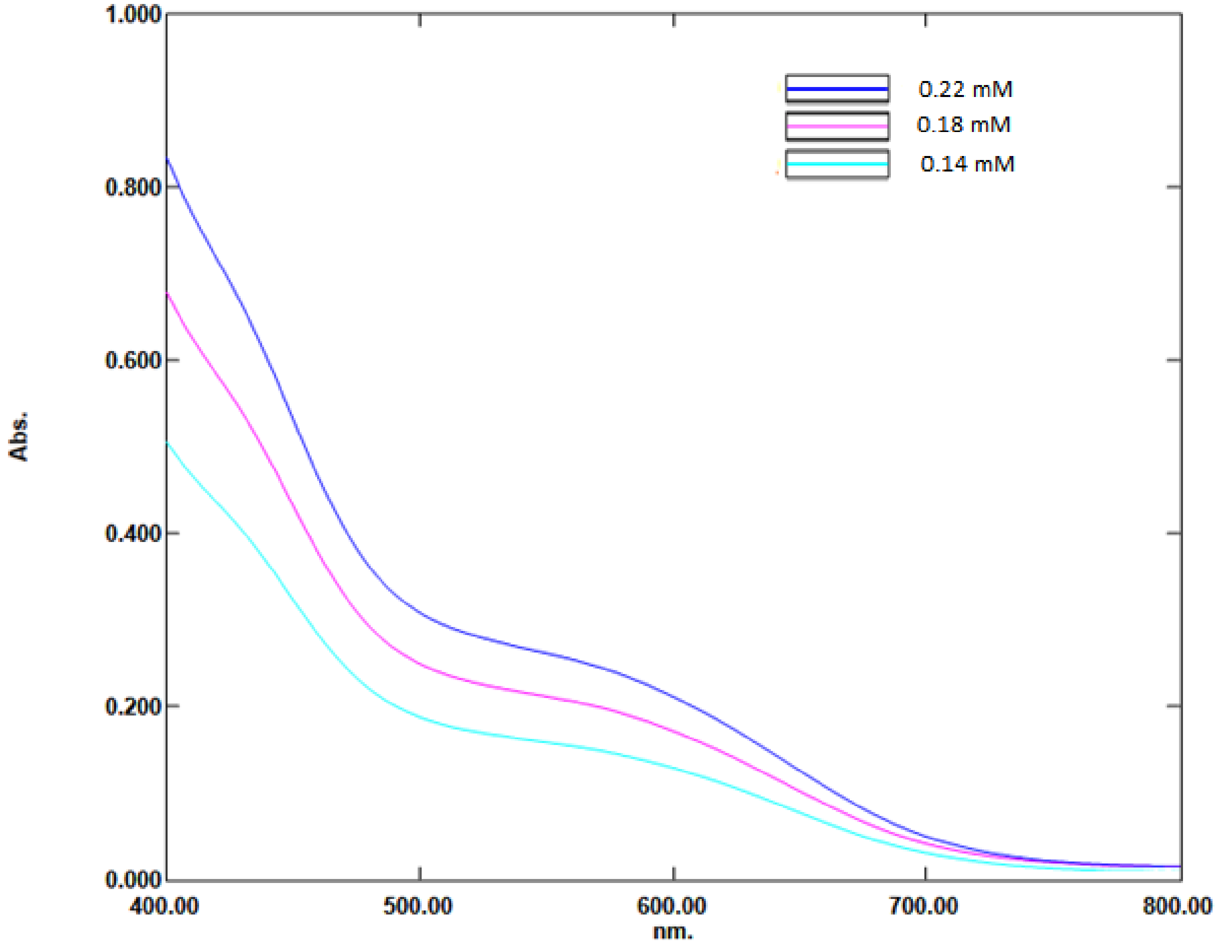

Figure S43. UV-Vis spectrum of $Cr[OO]^{Ph}(N(3,5\text{-}(CH_3)_2Ph))_2$.

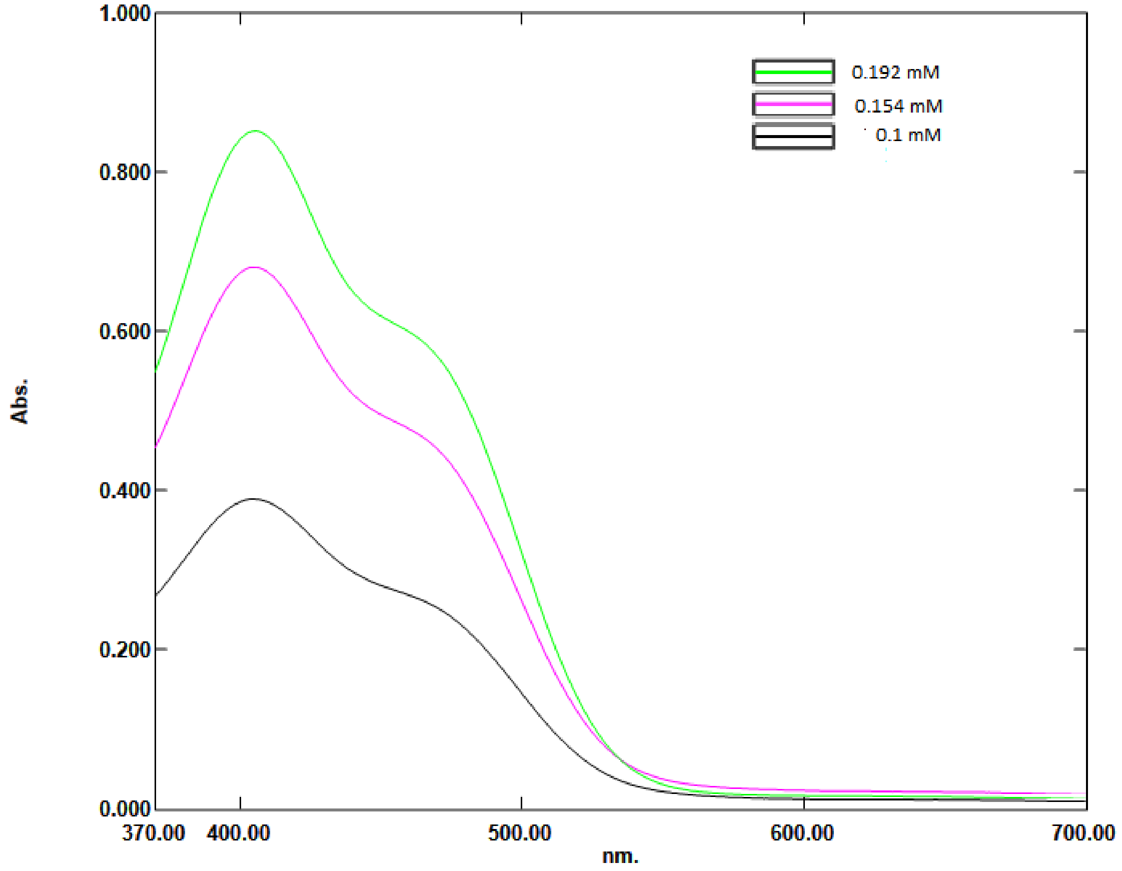

Figure S44. UV-Vis spectrum of Cr(OO)^{Ph}(CN(2,6-$Me_2C_6H_3$))$_4$. λ_{max} (ε_M) : 405 (5050) and 466 (3500)

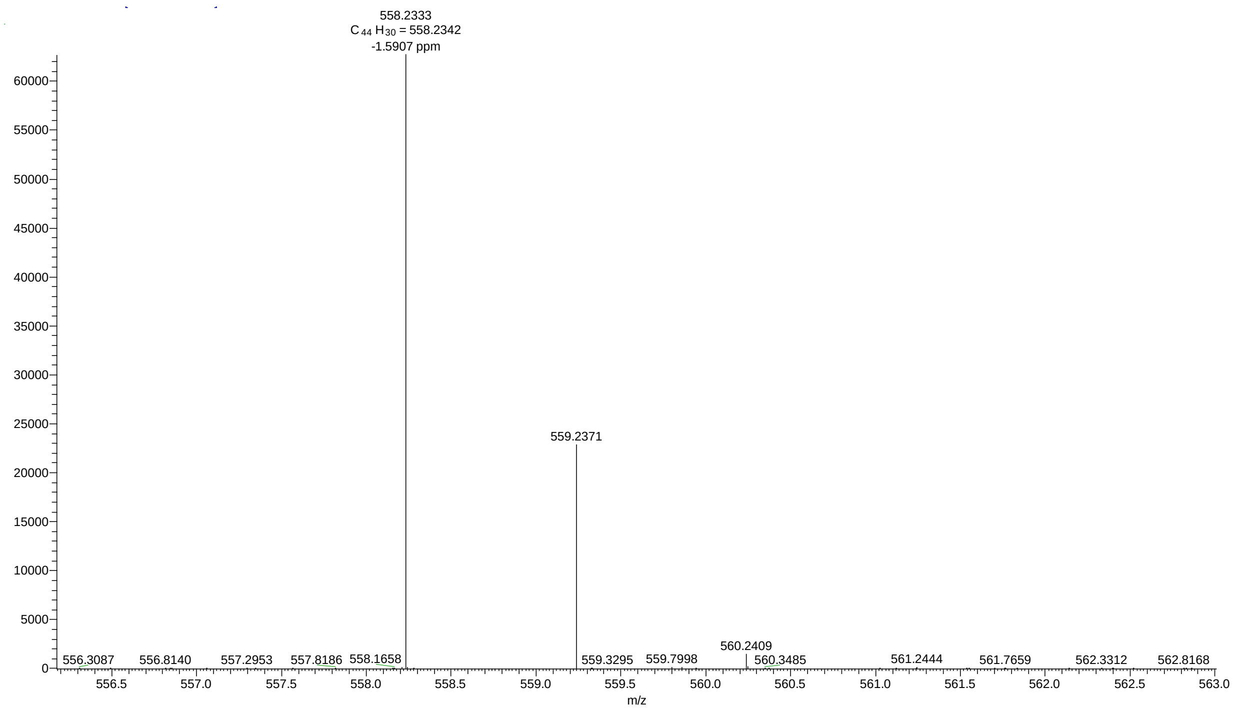

Figure S45. HRMS of 6,6,12,12-tetraphenyl-6,12-dihydroindeno[1,2-b]fluorine.

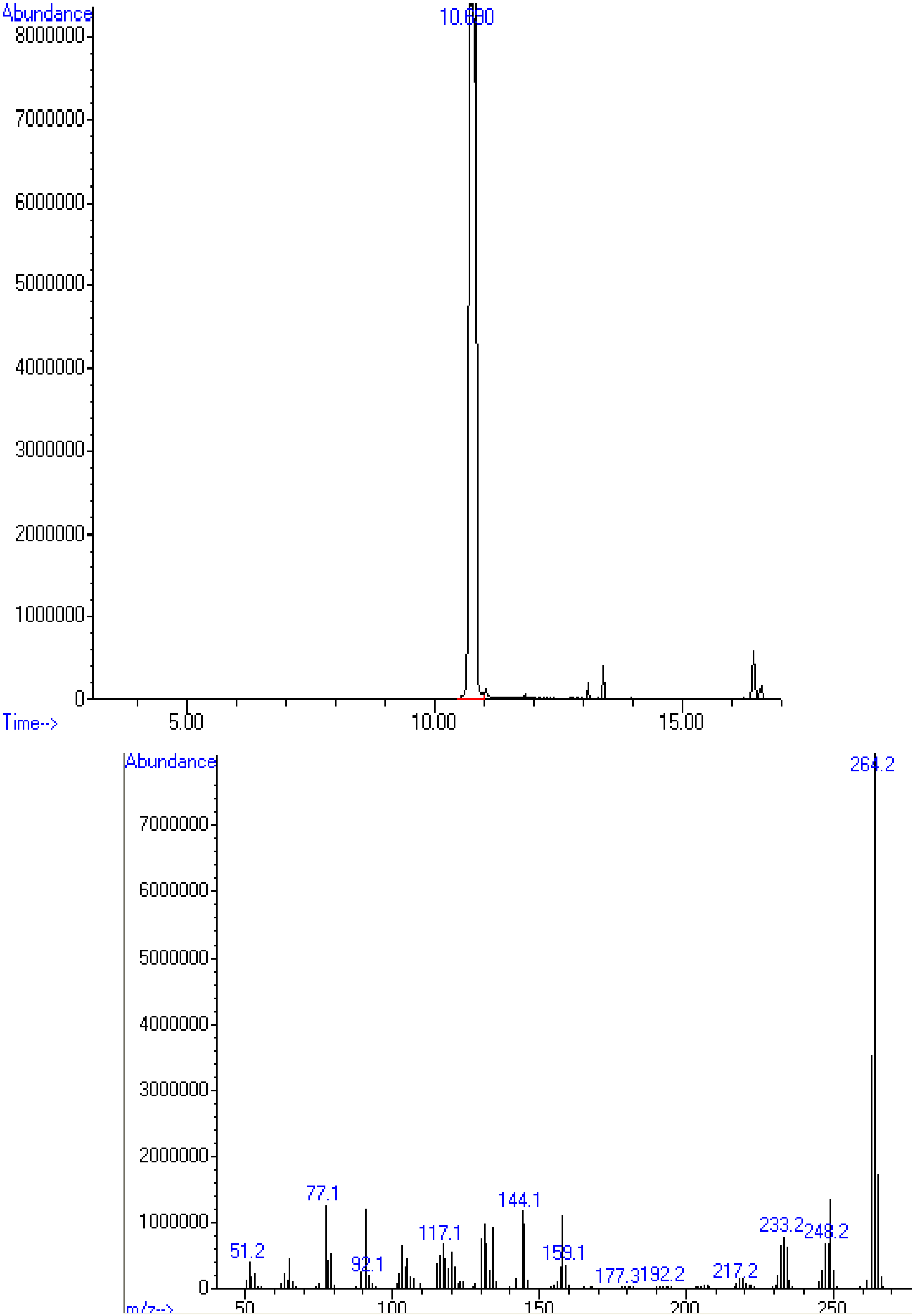

Figure S46. GC-MS of MesPhN=C=N(2,6-$Me_2C_6H_3$).

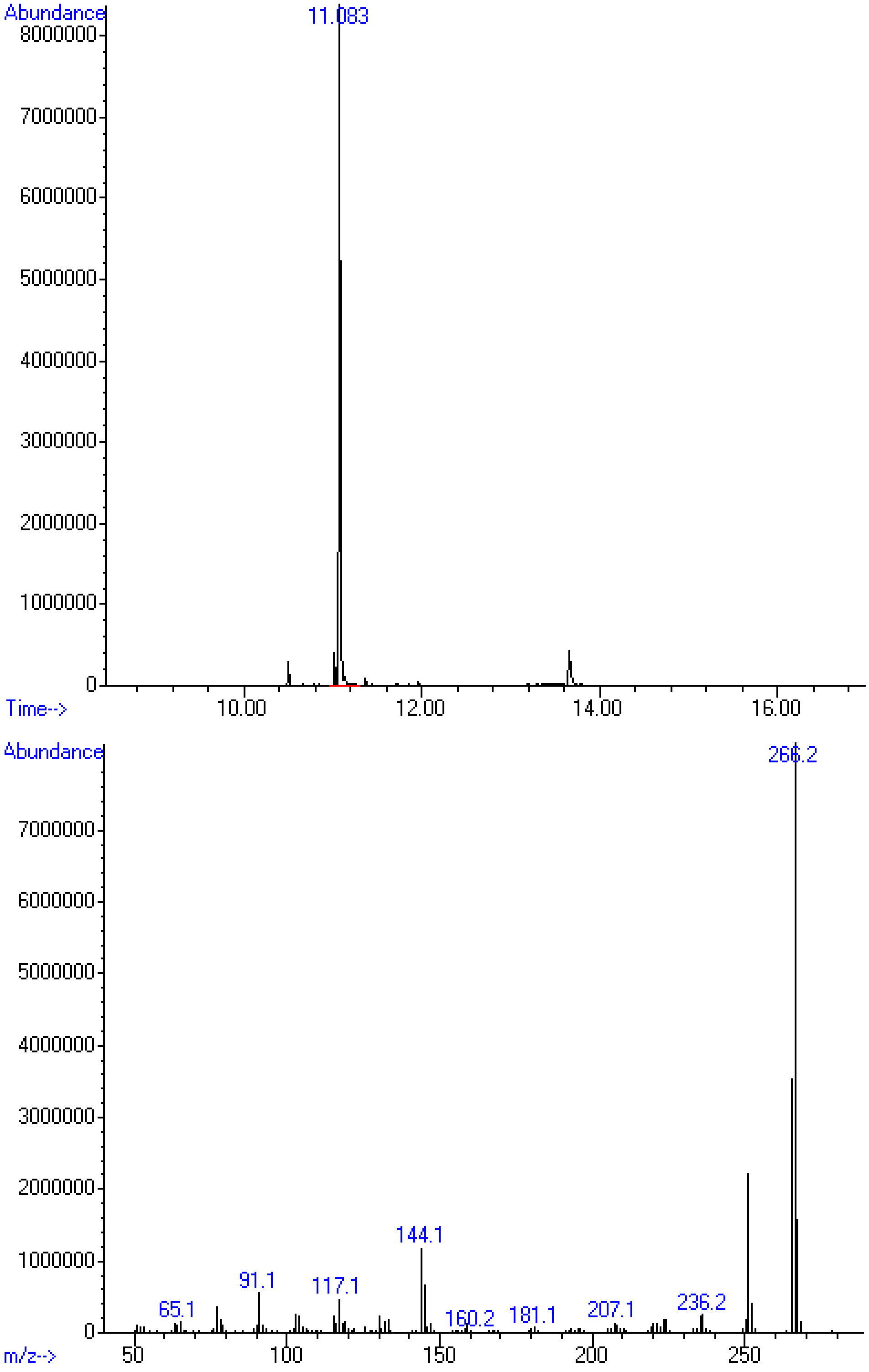

Figure S47. GC-MS of MesPhN=C=N(4-$OMe_3C_6H_4$).

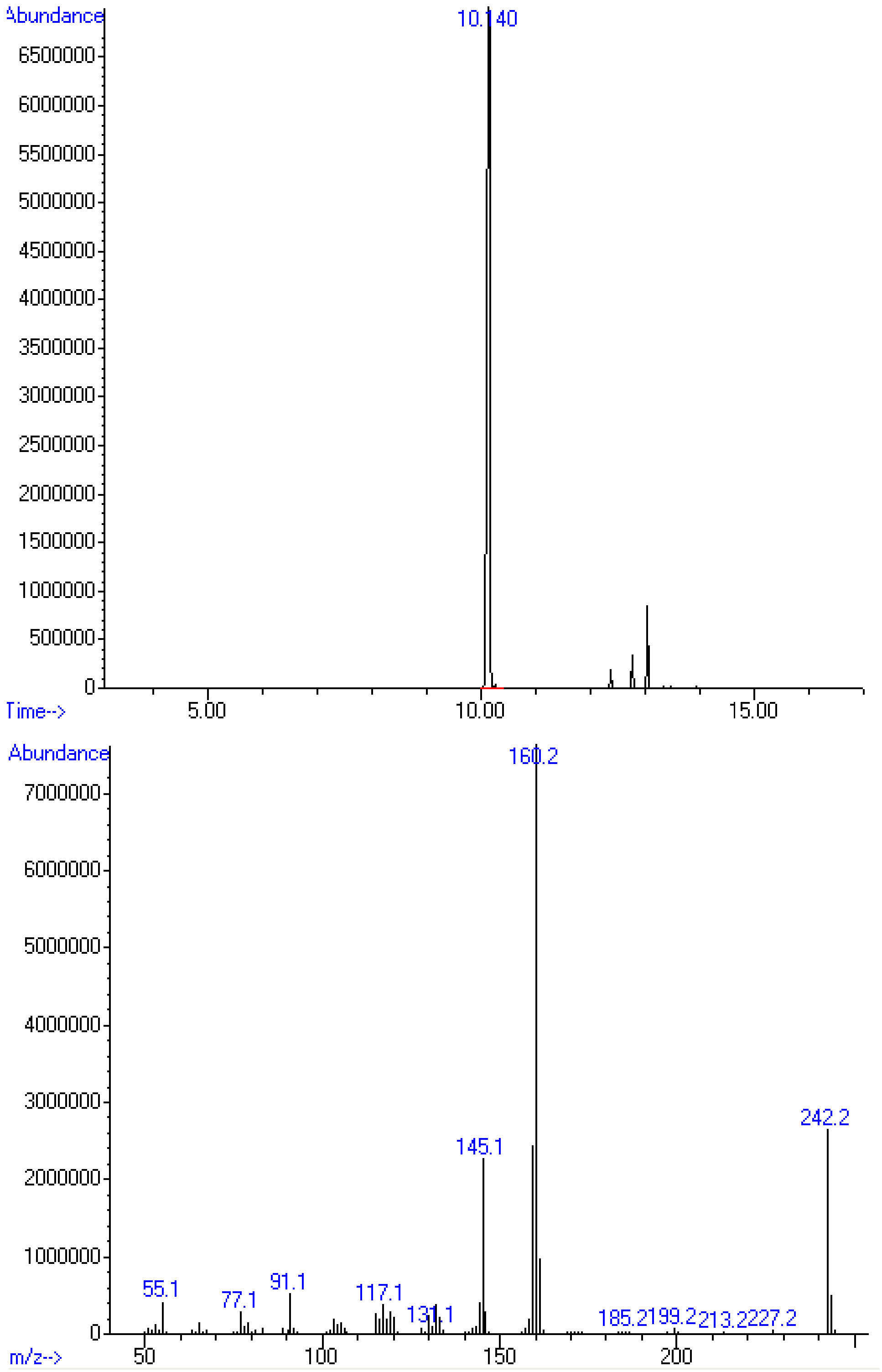

Figure S48. GC-MS of MesPhN=C=NCy.

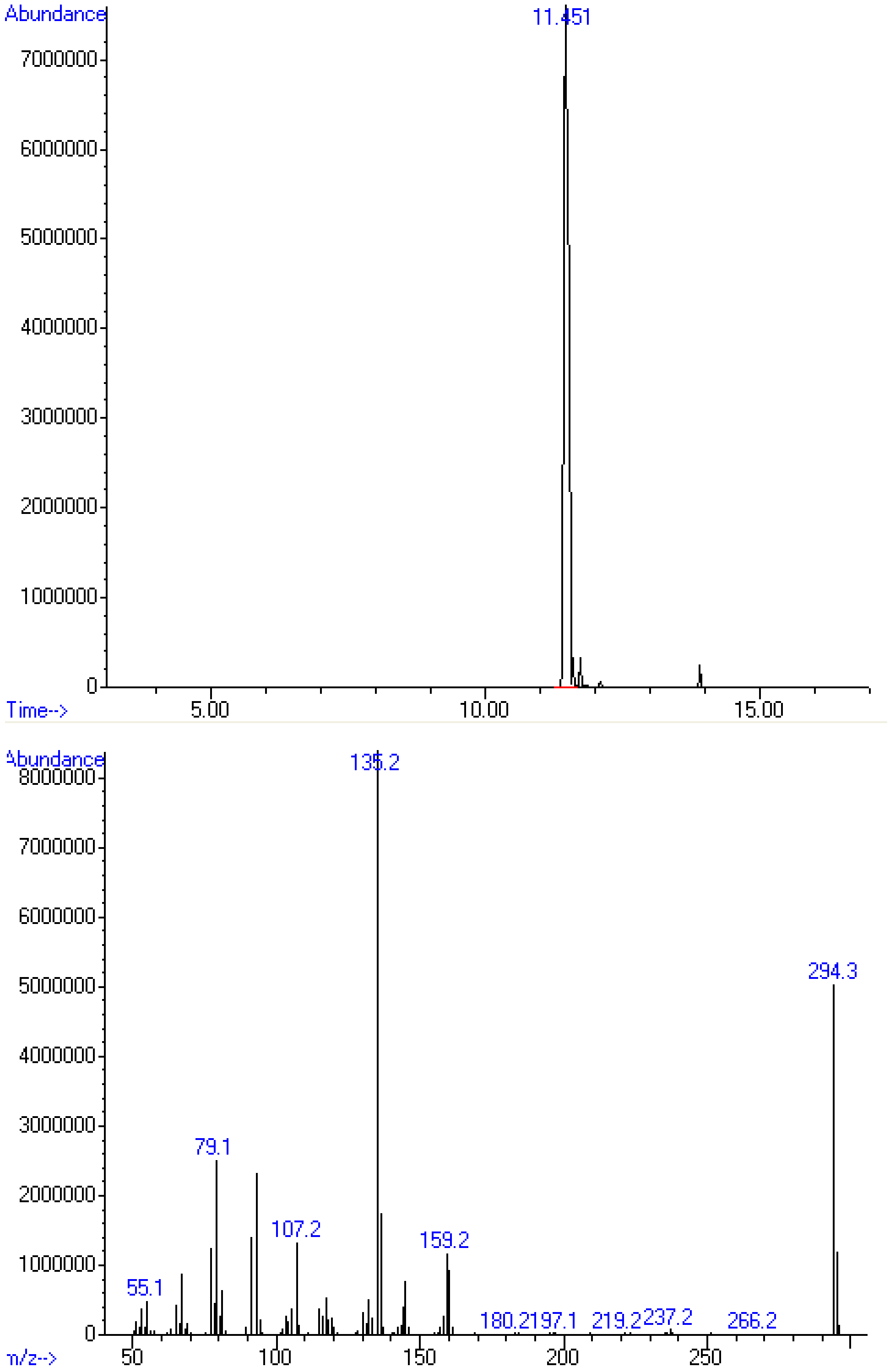

Figure S49. GC-MS of MesPhN=C=NAd.

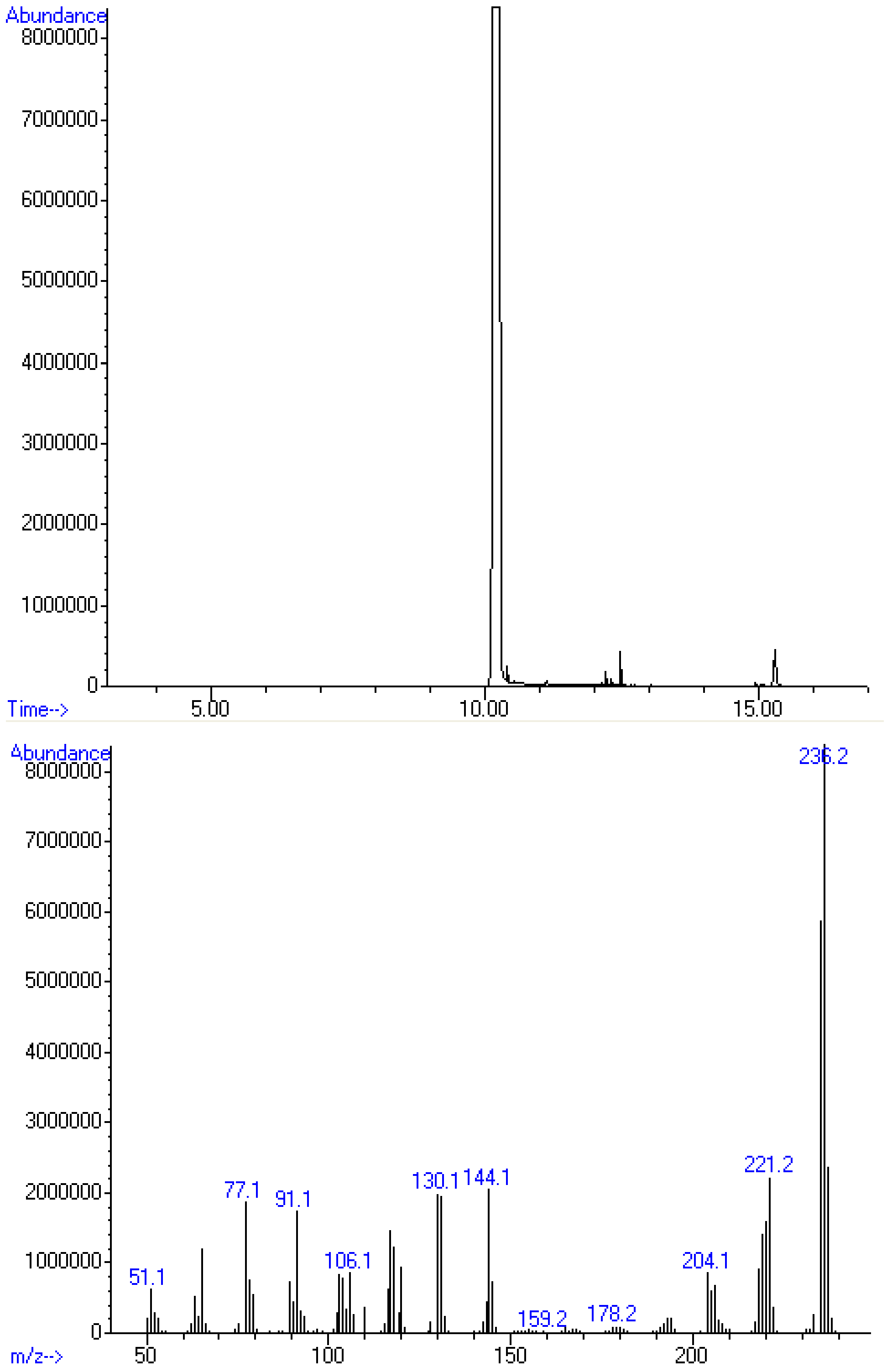

Figure S50. GC-MS of $(2\text{-}MeC_6H_4)N{=}C{=}N(2,6\text{-}Me_2C_6H_3)$.

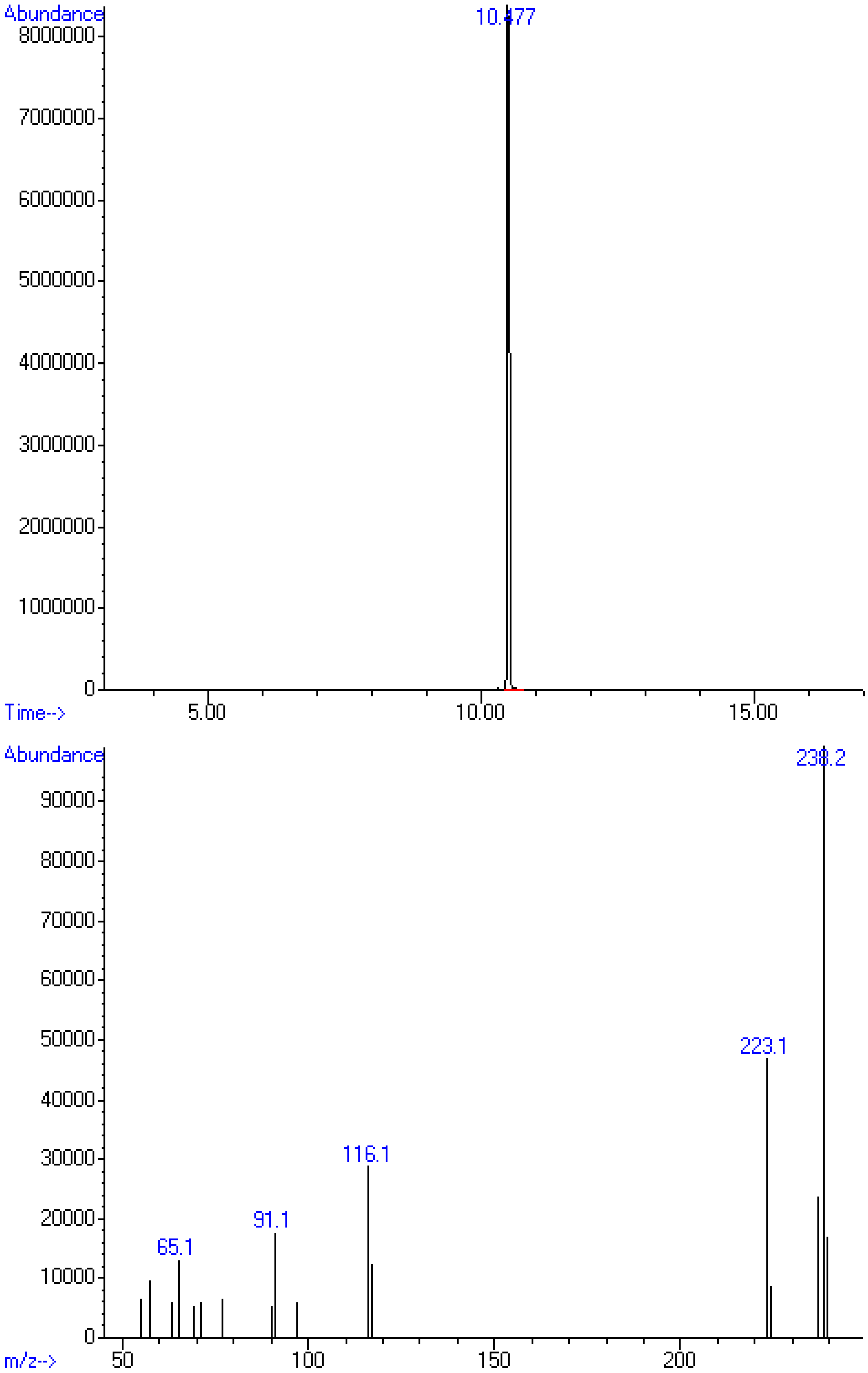

Figure S51. GC-MS of (2-MeC_6H_4)N=C=N(4-$OMe_3C_6H_4$).

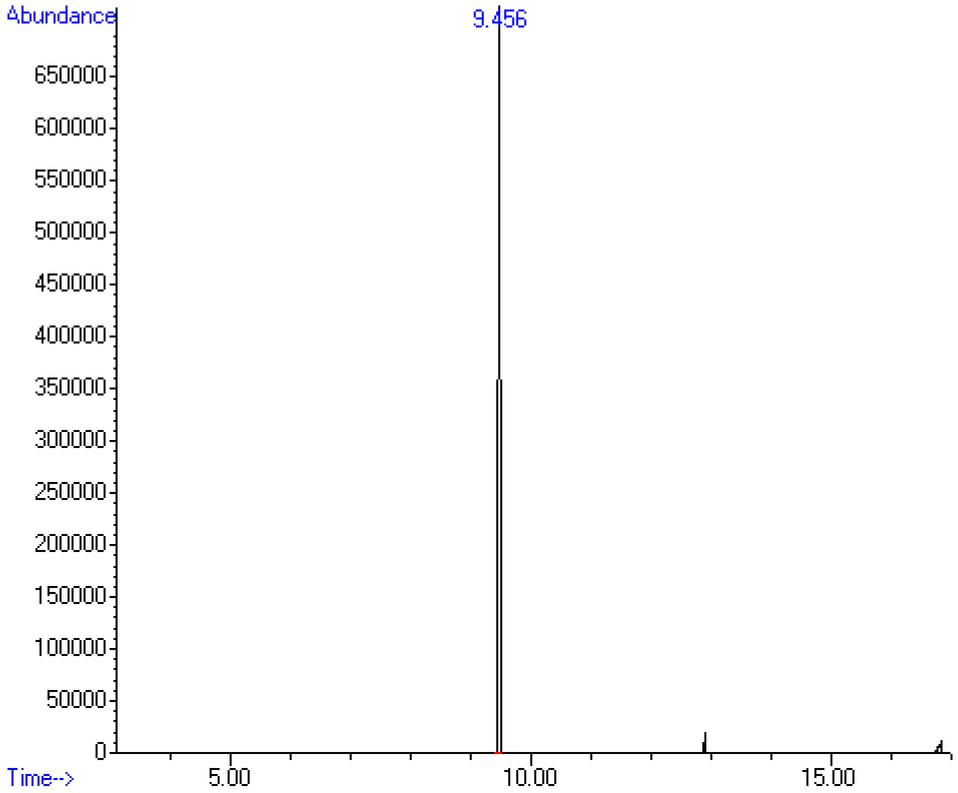

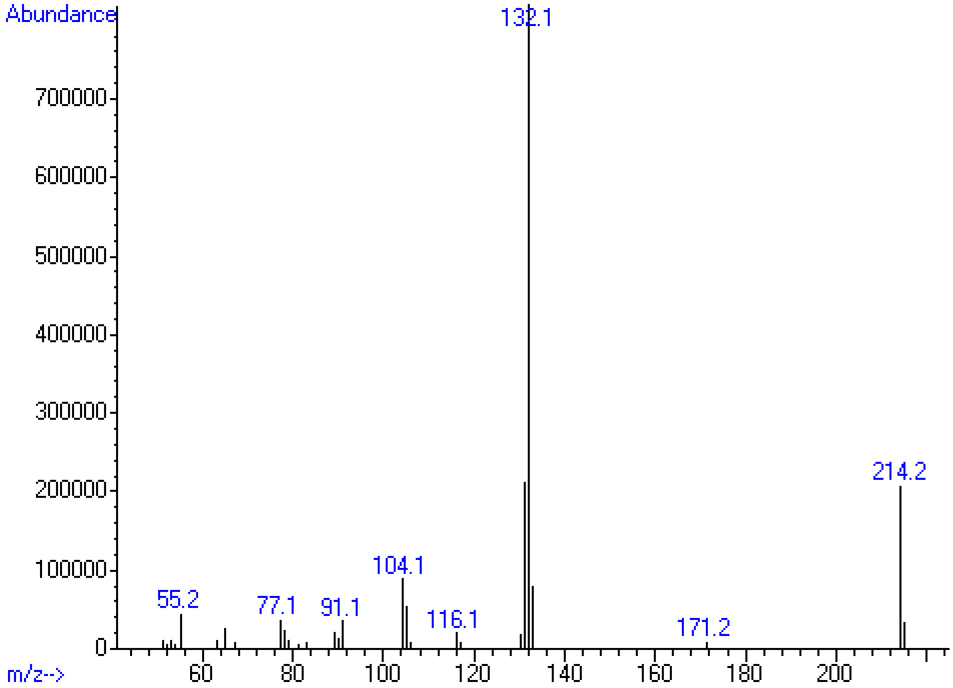

Figure S52. GC-MS of (2-MeC_6H_4)N=C=NCy.

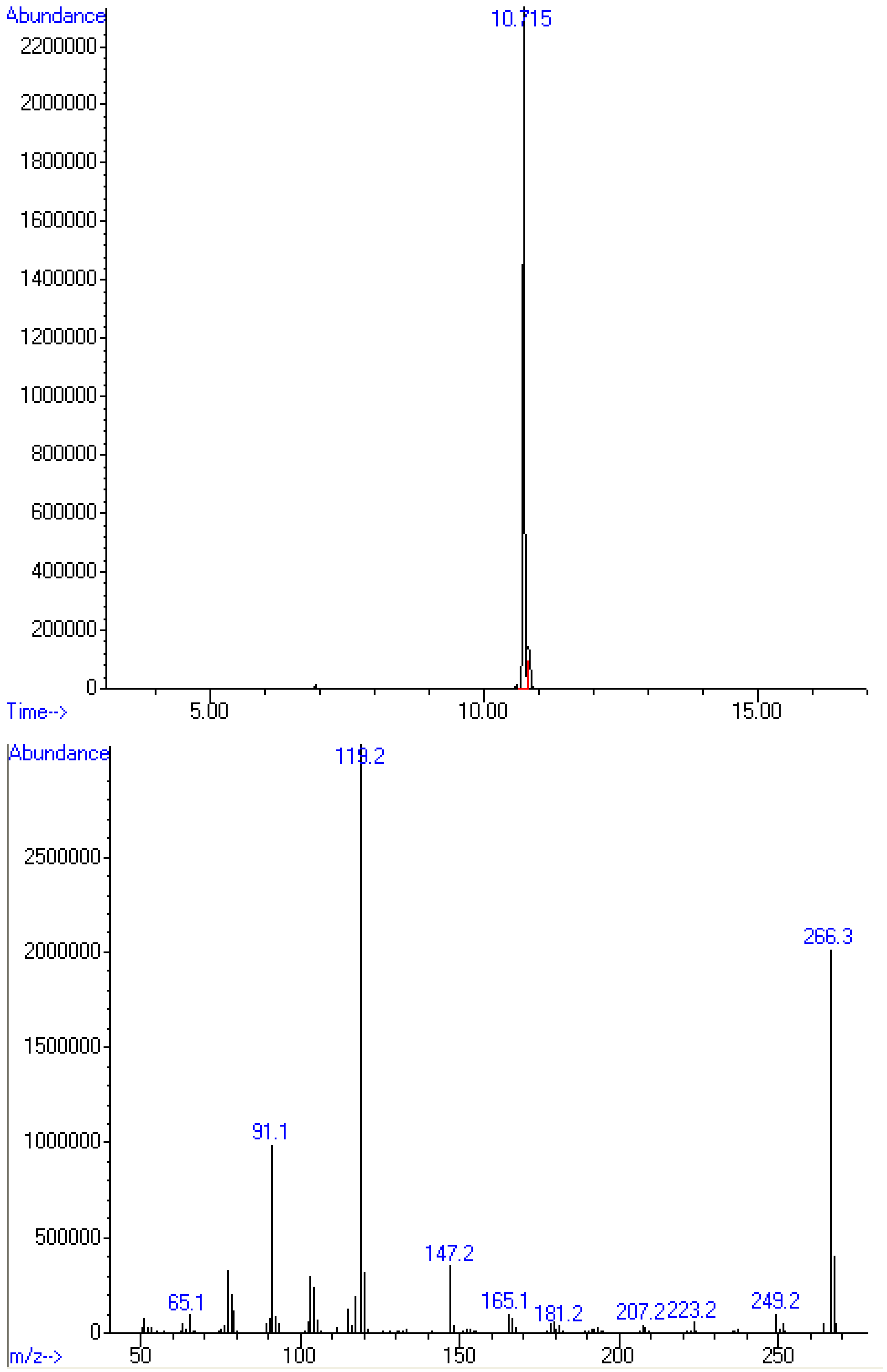

Figure S53. GC-MS of (2-MeC_6H_4)N=C=NAd.

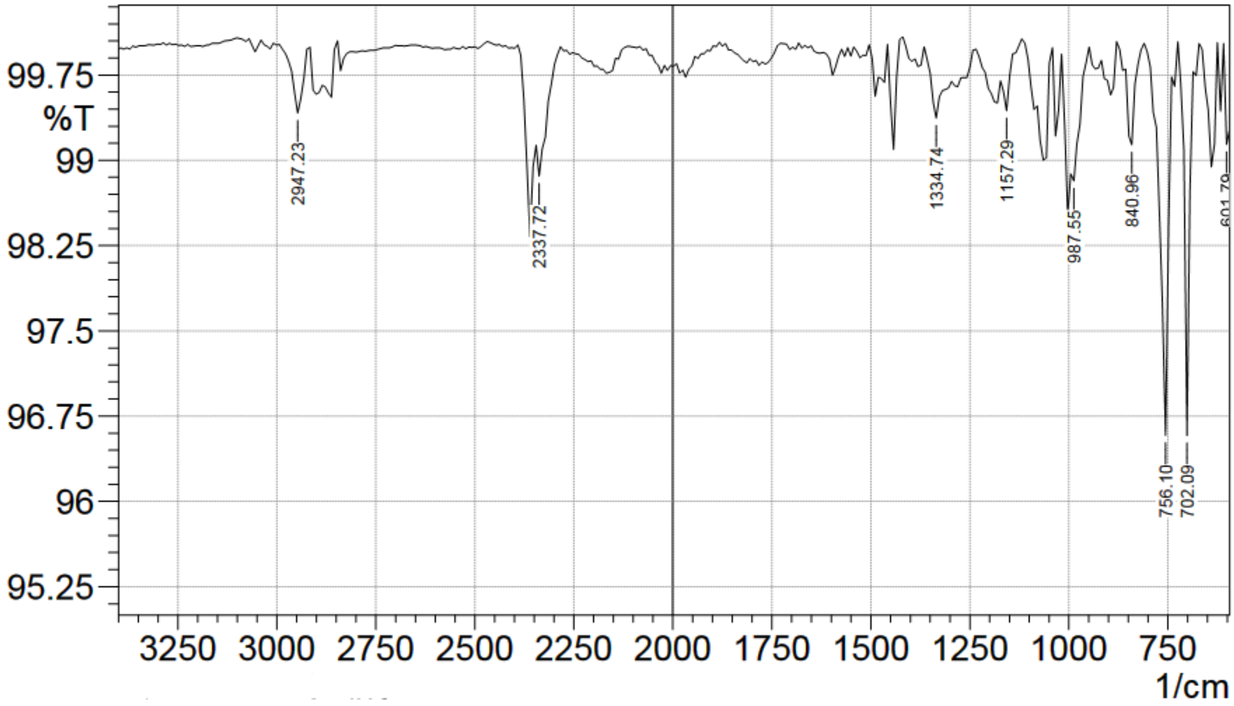

Figure S54. IR spectra of $Cr_2([OO]^{Ph})_2$

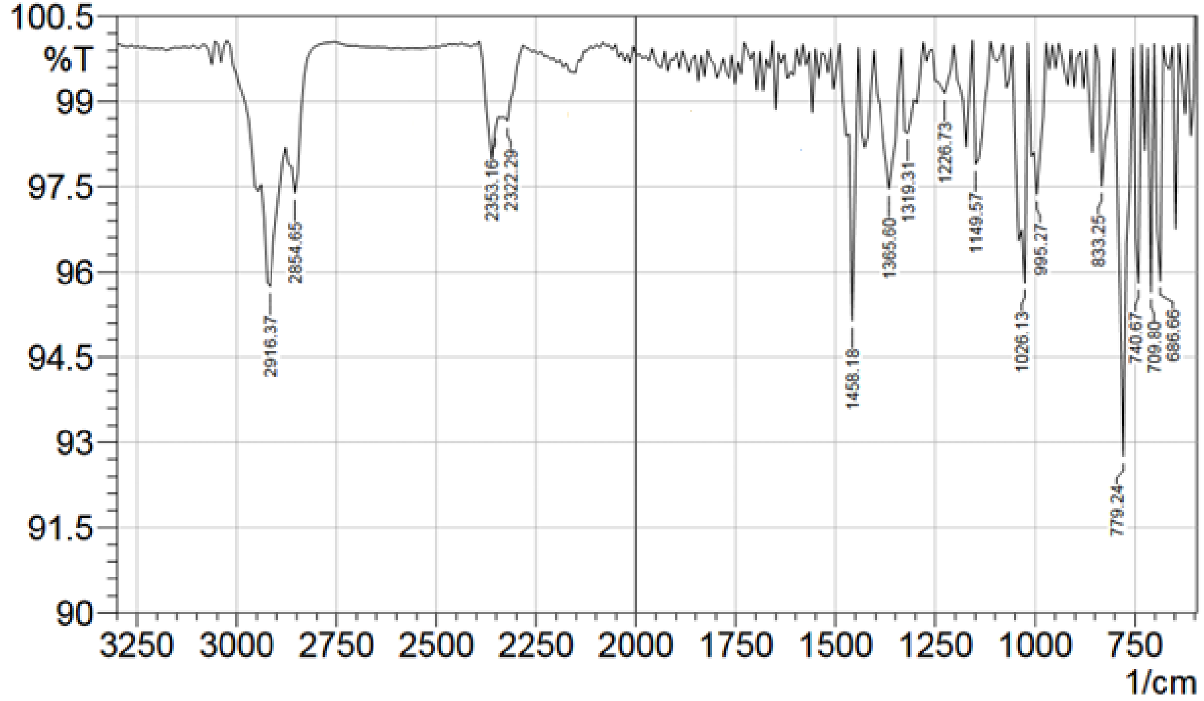

Figure S55. IR spectra of Cr[OO]^{Ph}(THF)$_2$

6. Computational data

Cartesian coordinates of all optimized structures are provided in the accompanying xyz file.

Table S1. Comparison of metal-ligand bond lengths (Å) in the optimized structures of **1** vs. the crystallographically determined parameters. O1/O2 correspond to the alkoxide arms, O3/O4 to the THFs, and C1/C2 to the bridging phenyl group (see Figure 1 in paper).

	**Cr–O1**	**Cr–O2**	**Cr–O3**	**Cr–O4**	**Cr–C1**	**Cr–C2**
**1**	1.924	1.935	2.156	2.277	2.49	
$\mathbf{1_Q}$	1.915	1.928	2.203	2.281	2.475	2.525
$\mathbf{1_T}$	1.858	1.857	2.586	2.230	2.116	2.127
$\mathbf{1_S}$	1.885	1.889	2.364	2.120	2.173	2.183

Table S2. Comparison of the Mayer bond orders in $\mathbf{1_Q}$, $\mathbf{1_T}$, and quintet Fe[OO]^{Ph}(THF)$_2$.

	**M–O1**	**M–O2**	**M–O3**	**M–O4**	**M–C1**	**M–C2**
$\mathbf{1_Q}$	0.69	0.67	0.31	0.25	0.25	0.23
$\mathbf{1_T}$	0.64	0.87	0.17	0.29	0.65	0.64
$\mathbf{Fe_Q}$	0.63	0.63	0.22	0.25	0.09	0.06

Figure S1. Spin density isosurface (iso = 0.002 au) plots for $\mathbf{1_Q}$ (left) and $\mathbf{1_T}$ (right). The transparent blue and white surfaces correspond to alpha and beta spin, respectively.

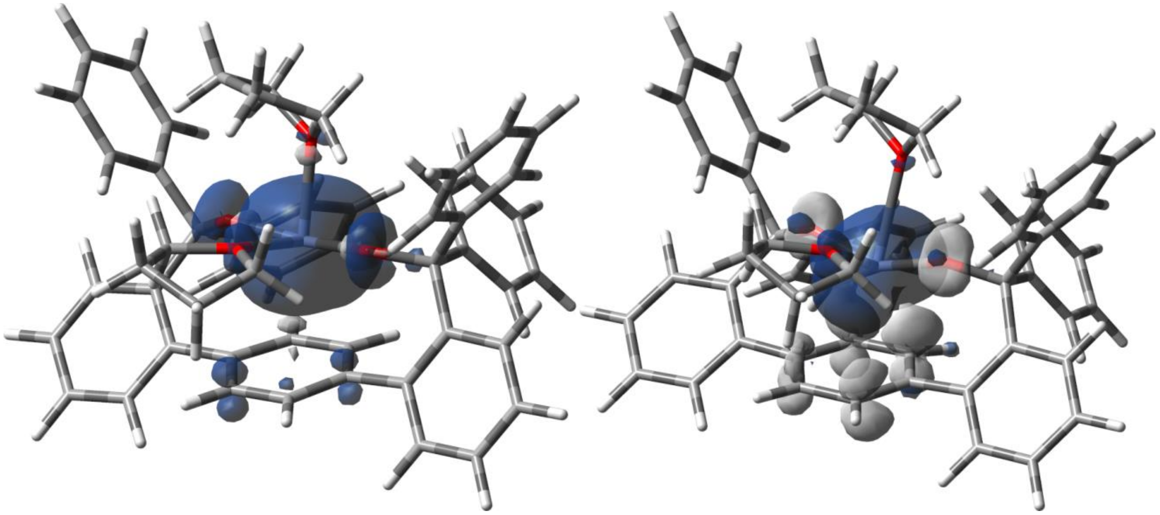

Table S3. Comparison of metal-ligand bond lengths (Å) and angles (^{o}) in the optimized structures of **6** vs. the crystallographically determined parameters. See Figure 3 in paper for atom labels.

	Cr–O1	Cr–O2	Cr–N1	Cr–N2	N1–N3	N2–N4	Cr–N1–N3	Cr–N2–N4
**6**	1.807	1.777	1.645	1.716	1.305	1.301	159.8	131.2
$\mathbf{6_Q}$	1.774	1.757	1.861	1.923	1.220	1.231	133.6	123.3
$\mathbf{6_T}$	1.760	1.751	1.683	1.815	1.239	1.231	160.8	133.8
$\mathbf{6_S}$	1.776	1.773	1.640	1.699	1.273	1.277	159.7	139.3

Table S4. Summary of thermodynamics of various spin states of **1** and **6**. All energies are listed in hartrees.

	E(SCF)	H(gas)	G(gas)
$\mathbf{1_S}$	-3354.455635	-3353.563256	-3353.698116
$\mathbf{1_T}$	-3354.466550	-3353.574521	-3353.712175
$\mathbf{1_Q}$	-3354.496495	-3353.603870	-3353.743635
$\mathbf{6_S}$	-4110.956897	-4109.893549	-4110.059202
$\mathbf{6_T}$	-4110.928821	-4109.867629	-4110.034745
$\mathbf{6_Q}$	-4110.897778	-4109.836275	-4110.004595

Table S5. Harmonic frequencies (cm^{-1}) for all optimized species. A small imaginary frequency for $\mathbf{6_T}$ was ignored since the singlet was much lower in electronic and free energy.

1_S

```
  14.5936     25.8106     28.8729
  33.5653     40.1415     42.3876
  49.1897     52.6772     55.6878
  60.5028     62.3817     63.3694
  66.3415     68.1072     75.0269
  81.1583     86.9660     89.9743
  96.8582    105.8751    110.5301
 111.4030    114.8848    118.8141
 126.8668    127.2522    133.3310
 141.1662    149.4872    173.5217
 176.3917    193.0275    194.4532
 197.1574    207.6308    220.0240
 225.2397    236.1277    243.1269
 247.8920    255.7526    259.3627
 266.2055    269.4169    277.7600
 287.3274    288.7996    305.2978
 308.3608    321.5534    335.2767
 340.3276    349.1947    357.7086
 388.4205    401.6809    402.1598
 406.3975    412.2624    414.4868
 424.0214    447.7754    451.1894
 470.4136    478.0902    488.5912
 498.9082    510.5587    515.0479
 521.5716    539.9122    546.1618
 556.4998    592.4076    608.3100
 611.0613    613.2546    613.4427
 613.6683    615.5729    625.0754
 628.5097    631.1630    633.1077
 635.4305    635.9578    642.9689
 650.7766    677.2863    683.1504
 694.2369    697.5923    698.1342
 700.7304    703.0156    708.7534
 721.9846    744.4009    745.8401
 748.8309    753.6014    756.9307
 766.0427    774.6765    775.9984
 780.0711    782.6711    800.5670
 817.6852    822.1459    829.1955
 830.6271    832.0776    834.5851
 846.3516    848.7627    861.8378
 862.6278    873.0425    876.9317
 884.9655    888.0035    894.1181
 898.4446    899.8252    900.5601
 904.0650    908.4834    915.4527
 918.1341    922.1563    928.7000
 932.3131    933.6774    934.7125
 936.9684    945.8053    946.2744
 957.7049    958.7012    960.5254
 970.9700    973.7923    974.2023
 978.2960    979.1889    979.7710
 981.4061    984.8342    986.6856
 988.1170    988.2225    989.5884
 990.2756   1009.3358   1015.3475
1024.7148   1025.4233   1026.7542
1027.8978   1029.0712   1032.4026
1037.2264   1039.6457   1042.5718
1053.1463   1055.8291   1070.4295
1071.1241   1074.1349   1075.5185
1078.9029   1092.5008   1096.5483
1097.7925   1119.9711   1133.0849
1133.1832   1133.7061   1134.8252
1134.9978   1138.8290   1140.1610
1142.3287   1145.6342   1146.0365
1152.7827   1153.6653   1155.2536
1156.7339   1161.1614   1167.5514
1168.0992   1169.2866   1178.8266
1179.4727   1188.5470   1202.7643
1207.0292   1208.4218   1216.5017
1233.5420   1235.9934   1256.3338
1256.9790   1258.1530   1263.6469
1268.5418   1277.3487   1278.8848
1283.5879   1284.5946   1290.6616
1291.5287   1308.4019   1312.7660
1321.9157   1325.5338   1332.5754
```

1348.8237	1361.3341	1366.0231
1366.5463	1368.3442	1369.6613
1386.9333	1402.9265	1407.0286
1410.1776	1412.0344	1419.8130
1423.9486	1425.2536	1428.0998
1429.1637	1430.8929	1431.8255
1438.3642	1445.4214	1447.6954
1450.2458	1465.5728	1468.1724
1469.4770	1471.2847	1471.3374
1474.8141	1532.5268	1540.4775
1571.5167	1574.2784	1588.6249
1589.8090	1590.9587	1592.1487
1601.4133	1602.5173	1605.4387
1605.7994	1608.7392	1609.2210
2926.1939	2932.6534	2949.9688
2974.5227	2988.5948	2992.5647
2996.1173	3002.8671	3041.9059
3042.5399	3051.4454	3058.3431
3066.1404	3078.9555	3090.3428
3091.5109	3091.7304	3092.7564
3092.9624	3095.4713	3096.6135
3097.5613	3097.7389	3100.3986
3100.6220	3101.7282	3104.7714
3106.7594	3107.8752	3109.8071
3110.9243	3111.5037	3112.3392
3113.7943	3114.3376	3114.9726
3115.2209	3115.8830	3121.3566
3122.0592	3122.5410	3123.2043
3124.6474	3124.6851	3126.4231
3126.9047	3134.6995	3140.1314

1_T

12.7876	26.2922	28.6776
32.8261	37.2695	40.8532
47.6778	49.8562	53.3450
54.6023	58.2924	62.0327
65.1614	68.8583	70.4249
71.8542	81.0753	85.3881
87.5095	90.1547	101.5207
105.0568	111.1861	113.0783
119.8340	125.3328	132.6920
133.1736	140.2012	164.0306
170.4789	178.6131	184.5164
195.0521	199.2548	213.7389
219.8351	234.1030	243.6921
248.1536	256.3082	259.9747
268.3845	270.4273	277.1217
287.2955	289.5304	307.1808
311.0159	320.3187	337.1353
337.9729	348.1719	359.8986
385.3737	401.3616	401.9525
405.6106	407.3822	411.8702
413.5124	445.0772	453.9612
465.8269	488.6455	490.2656
507.1635	512.8919	514.4903
521.7821	541.4210	544.5427
557.4415	606.4906	609.4157
611.8232	613.2321	613.4183
613.6381	616.0037	618.3054
631.8840	633.6010	636.2501
636.6639	636.9220	649.2012
654.0063	671.7220	685.5759
691.1151	697.6844	698.3258
701.4266	704.5146	708.5285
723.3309	745.3560	746.6172
749.3476	753.1868	756.8857
764.8047	774.3308	775.1932
782.5277	783.8996	795.5557
807.9362	813.4113	819.5234
828.8963	830.8031	831.8286
835.3316	847.8854	851.4559
862.9354	864.2895	876.3829
880.6263	885.1699	889.1273
892.6923	899.3891	903.1943
906.1370	911.7868	914.9522
919.5750	922.6854	928.2730
932.7923	933.6576	935.7257
938.9336	947.1136	947.5429
957.9837	959.3830	960.5953
972.3325	975.3375	975.7949
978.9354	979.6731	979.8653
981.0791	983.8644	985.5326
987.2834	988.3260	988.8088
994.1627	1005.9629	1014.2536
1014.4959	1025.0812	1026.1409
1027.0677	1028.2960	1029.3484
1030.9675	1043.1548	1051.7475
1053.8232	1054.8245	1060.3816
1071.7778	1072.0541	1075.2214
1079.0912	1093.8478	1094.9529
1095.9030	1122.0728	1131.8949
1133.6877	1133.8274	1134.1005
1135.5108	1139.0245	1140.1299
1143.2447	1145.1549	1146.7177
1153.8609	1155.2886	1156.5912
1157.0094	1164.3304	1167.5246
1168.2019	1169.9010	1182.2675
1183.7242	1191.5279	1204.0869
1207.1235	1210.7141	1216.6717
1229.5792	1235.3489	1253.7663
1256.5083	1257.4462	1261.7907
1267.0901	1272.9715	1280.7703
1284.6418	1285.5630	1290.7845
1294.1318	1307.6965	1312.4095
1319.4154	1330.2667	1335.2434
1347.7158	1360.8967	1366.1048
1366.8614	1368.6180	1369.9209
1378.9254	1397.4448	1402.4301
1407.2857	1411.9951	1419.8962
1422.7604	1424.7985	1428.6880
1428.8649	1429.8173	1432.4609
1433.5346	1440.1351	1443.0350
1451.2445	1460.5050	1466.2235
1471.0380	1472.0244	1472.0770
1476.9787	1532.4623	1538.2071
1571.9648	1574.9338	1589.5762
1590.4434	1591.4490	1592.8797
1602.0444	1603.2415	1606.2595
1606.7156	1609.4631	1610.1153
2908.3291	2920.3648	2936.7927
2975.9319	2983.6240	2988.8897
2992.4381	3004.8046	3039.8719
3043.0659	3047.8784	3053.1473
3061.7467	3063.4505	3089.7811
3092.7118	3093.5442	3094.3703
3096.5487	3096.7352	3096.9707

3097.4543	3099.2039	3099.7015
3101.9825	3102.7646	3104.7429
3105.8958	3107.5883	3109.4243
3110.5047	3112.4191	3113.8767
3115.3170	3115.5338	3115.8654
3117.4645	3118.1289	3121.6598
3123.0400	3123.1095	3124.7208
3126.4571	3127.6446	3128.1974
3129.1741	3134.7858	3145.0586

1_Q

11.6436	21.7555	25.9547
30.4243	38.4028	40.9800
48.5456	51.0127	54.0321
56.8126	58.5096	60.2310
62.9392	67.5573	70.5211
72.0687	80.1781	83.0973
88.9923	91.0672	98.9614
103.8856	106.2883	113.9778
115.6319	119.9013	124.3162
129.9282	135.3001	156.7249
163.6917	168.9815	176.8450
193.4803	194.7427	200.2840
214.2202	216.8359	227.1210
230.0322	240.5802	251.7638
253.2266	265.3786	267.1901
276.4463	280.0092	287.6509
295.5963	310.1693	321.0985
334.0975	341.7868	348.5165
380.2531	400.8710	401.4216
404.4276	407.6659	408.6925
410.2677	420.0943	433.3789
464.6022	470.8789	485.7366
498.3745	509.4085	516.5963
521.6929	537.1452	548.1196
558.8569	583.2271	611.3088
612.5359	613.0472	613.2892
613.5392	621.9413	623.1900
628.3891	629.8295	633.4819
635.2621	636.4671	641.4997
649.5521	672.2102	681.6748
696.9917	698.3101	700.4771
702.1014	707.2125	717.9763
722.0490	745.2206	747.1109
749.8701	755.5790	759.0742
769.2088	775.2478	776.1884
780.0397	782.7582	815.4127
826.1514	828.6329	829.2029
830.3448	831.6168	833.0362
841.9509	851.2274	863.1855
864.2547	878.1926	881.2900
885.4793	889.8546	893.2482
898.0781	900.8383	905.0546
913.9450	917.2572	923.8748
928.1511	930.3370	931.8827
932.9205	936.3816	937.2467
944.2418	945.7998	947.6382
957.1553	957.9131	958.1769
966.0886	975.1431	975.3493
978.0613	978.0885	979.8529
982.3238	984.3252	986.6864
987.1304	988.0247	988.4319
998.4756	1013.6982	1019.0301
1024.4510	1024.8932	1025.9747
1027.5417	1027.7657	1033.0030
1045.8538	1048.5737	1051.0724
1061.2303	1064.4124	1069.8731
1070.5108	1082.9095	1087.0152
1093.0979	1097.2819	1098.7301
1105.3228	1121.6027	1131.9137
1132.4141	1132.9639	1134.4940
1138.4998	1139.9667	1142.7122
1145.2017	1145.9861	1151.7425
1153.3287	1156.1844	1156.8049
1158.0607	1160.5450	1166.1824
1168.5591	1171.7968	1176.5175
1184.2976	1186.7018	1201.6477
1207.3687	1210.3456	1219.6302
1238.1674	1238.6070	1255.3384
1260.6357	1262.8914	1267.0592
1274.5191	1277.3044	1281.3337
1281.9622	1283.9481	1288.4978
1289.2058	1309.1781	1311.9345
1322.3376	1328.8804	1334.4511
1348.2649	1354.3307	1365.8120
1366.3746	1368.3687	1369.5728
1396.8127	1398.7173	1406.5732
1408.4413	1417.4259	1421.5063
1424.2060	1425.0481	1427.3412
1428.4689	1430.5225	1431.1315
1443.3818	1447.0139	1456.1459
1456.6559	1467.4123	1468.8331
1470.2166	1470.3878	1474.4462
1497.0927	1540.8387	1573.6277
1576.8282	1587.7816	1589.0223
1590.3121	1591.3572	1593.2476
1602.6037	1603.5208	1604.7524
1605.0696	1608.0367	1608.4798
2921.7737	2939.4066	2945.9647
2974.0853	2985.9741	2987.9083
2997.6335	3006.1748	3035.2998
3043.7046	3049.4322	3050.6333
3064.2016	3064.5875	3084.4105
3090.4096	3090.5446	3091.2474
3093.3257	3094.9350	3095.3033
3096.5223	3099.0952	3099.3561
3100.1614	3100.2205	3102.0277
3102.5903	3104.9688	3110.9849
3111.1387	3112.7177	3112.9981
3113.2651	3114.1044	3114.2635
3115.5761	3120.7711	3120.9332
3121.7669	3122.5495	3122.7851
3124.4943	3125.5597	3125.9056
3129.1427	3133.5462	3138.0641

6_S

9.7493	11.2998	18.4475
20.3269	26.8579	35.8122
37.2225	42.7129	44.4685
45.4915	52.6229	54.1554
56.3321	57.9656	61.7054
63.5838	67.3342	68.0924
72.4341	77.5574	79.1967
80.4246	84.2222	91.8352
92.8450	96.1850	101.2747
102.0164	106.9225	109.7931

112.0087	114.6513	117.6444
122.4730	125.6221	129.2936
153.5104	172.6334	184.4189
190.5154	202.9676	214.9521
219.9736	222.1749	230.8784
233.1584	236.3481	239.0911
241.7623	248.8984	260.6667
269.9721	276.3957	282.7928
287.7701	289.7078	292.4501
295.1034	301.0715	314.9078
319.7757	326.8744	337.2481
342.0772	346.1080	357.0744
377.8605	391.7895	398.7623
399.0659	404.1035	405.9690
408.8402	409.4676	410.6105
415.5492	417.5359	418.5175
426.1806	428.8745	437.7335
441.1233	466.8384	476.6807
482.7992	489.3595	503.3533
508.1758	510.0593	521.8210
528.2248	533.3138	542.0796
552.4196	557.2745	577.9445
587.4632	607.6069	607.8783
611.1763	611.8655	612.6074
613.6569	613.7840	614.4407
616.3585	618.9214	626.9610
630.5368	632.1736	633.5163
635.2728	637.3323	643.2134
648.2894	661.1527	667.8815
687.8198	688.4066	694.6930
696.8223	696.9807	700.4857
701.4990	705.1452	706.4759
712.9296	719.3140	724.0094
729.1709	735.0937	737.5196
747.0920	749.0790	752.4630
755.2810	757.1223	760.9203
767.0446	768.5495	774.9993
779.1281	779.8242	789.5336
809.8192	813.2111	814.7311
826.9743	827.2679	829.9438
831.2920	834.9145	835.0415
835.8884	842.7519	843.4932
863.3489	866.9145	887.7488
893.6967	900.6789	905.0151
908.0675	911.3707	912.2137
916.6176	920.2266	925.0801
929.7132	932.9275	934.0691
942.5847	943.4971	946.3853
949.5102	954.5177	957.9776
961.0445	962.1255	963.6309
964.9592	968.4837	968.9259
970.2911	974.3381	975.8856
976.6272	981.3281	982.2621
983.4230	985.1669	985.4170
985.6472	985.9846	986.5847
986.6966	987.2512	987.9833
988.3136	988.8712	989.3598
990.8375	993.9235	1003.7851
1021.6601	1022.2212	1022.6777
1024.0418	1026.2069	1026.7316
1027.2984	1028.1899	1029.8960
1032.6860	1043.4078	1051.7771
1056.9460	1068.2013	1071.8992
1073.3491	1073.6671	1074.4253
1074.6926	1077.6669	1084.2679
1088.8359	1095.6266	1099.0706
1132.4563	1132.9554	1133.0328
1134.0509	1134.7251	1135.5975
1135.9876	1136.3530	1136.7161
1137.4731	1139.2525	1144.3364
1144.9899	1148.5160	1152.1472
1154.0233	1154.6102	1155.5800
1156.8064	1160.5514	1162.0258
1162.8453	1163.2802	1176.6526
1179.0277	1185.0889	1188.8295
1238.6137	1241.5376	1259.1927
1270.7839	1271.5453	1274.9965
1275.4238	1281.6208	1281.8344
1283.7576	1284.8962	1288.3154
1290.4031	1301.2576	1322.4159
1328.2981	1338.9614	1342.2368
1349.7944	1362.2411	1363.7358
1366.7740	1367.5263	1368.7694
1369.7041	1369.9363	1375.8512
1395.4059	1403.1453	1418.5548
1426.5190	1427.5657	1429.3738
1430.0098	1430.2850	1430.8135
1431.9902	1433.6540	1440.7378
1455.9449	1469.7226	1471.4172
1471.8129	1472.0986	1472.3584
1474.7743	1475.0932	1476.2243
1478.0583	1487.5415	1503.2455
1514.1482	1552.4828	1572.0036
1576.6661	1577.3283	1578.0972
1582.6517	1583.2746	1588.7601
1589.8095	1592.3033	1592.6621
1603.2354	1604.7992	1605.3599
1606.1552	1606.6896	1607.1434
1610.1807	1610.5936	1610.8905
1612.2149	1613.3901	3095.2656
3095.3568	3095.6745	3095.8341
3096.7183	3097.2719	3097.4256
3097.9537	3099.8941	3100.3375
3100.4520	3102.8509	3104.1033
3105.1576	3108.5352	3109.3883
3109.4459	3109.7577	3109.9139
3111.3643	3111.5859	3113.2012
3113.2567	3115.0725	3115.3237
3115.6006	3117.3536	3118.8051
3119.2857	3119.5172	3119.9529
3121.5241	3121.6990	3122.6231
3122.9058	3123.9017	3124.5417
3126.0827	3126.3107	3126.9178
3128.0032	3128.0896	3130.1136
3131.7471	3132.3135	3134.0898
3135.6313	3137.6189	3138.1817
3142.2156	3145.7576	3162.0238

6_T

-1.2730	7.2271	17.5145
19.2022	25.0209	26.5368
28.8120	36.2977	36.7590
42.0707	44.2150	46.5872
49.6846	52.3688	55.4895
58.2038	61.0829	69.6661
70.3231	72.5249	73.2819

77.2012	78.6115	81.5821
87.0582	91.3393	99.0724
101.3209	102.3066	106.1324
111.1208	113.7269	115.5955
118.2493	121.1908	129.0019
151.2173	158.5077	183.2729
188.7187	197.1650	208.9019
212.3359	216.5995	220.0501
222.8373	226.5000	236.3187
237.7054	244.9900	251.0197
261.6872	263.0614	271.7340
287.0035	288.5321	293.8506
295.2275	301.2036	302.2861
311.1179	319.5920	323.4996
330.7957	345.1427	348.5862
363.3172	379.4729	392.6772
399.6890	401.1538	403.2831
404.5853	408.4883	410.4063
411.6494	412.2668	413.1503
414.5472	420.0157	425.4185
431.3581	446.5673	456.8771
468.6799	476.3949	488.2988
496.4377	507.7663	515.2180
519.0768	527.3585	534.1063
541.3887	548.7837	553.1985
576.5115	581.7678	594.0986
597.6789	605.4958	612.1612
612.5588	612.9249	613.5638
613.9421	614.6454	615.6130
616.1347	617.1100	625.8429
630.5220	632.7175	634.2623
637.1969	638.6808	663.5315
670.2818	688.9954	690.4313
695.7783	697.6817	700.1046
700.3055	701.2083	703.5164
704.5901	705.7865	713.5083
727.7556	731.9610	737.0274
744.7221	748.3457	748.9231
751.7314	752.4288	756.9983
760.6107	763.4120	765.2811
768.8058	769.1279	778.4323
807.6043	814.3143	816.1235
820.9393	824.8124	826.7135
831.1142	833.9773	835.5784
839.1447	840.2480	842.4801
865.7795	866.8340	889.0167
895.8120	897.8336	900.2500
903.8970	908.5784	910.0197
911.0270	917.9912	923.0426
923.7321	930.2817	932.8205
933.8503	934.7503	945.1704
945.7128	955.9652	956.8550
957.7044	960.2649	964.6858
965.5908	966.5766	967.4244
968.2485	974.5185	976.6573
976.8012	977.0555	979.2304
981.2157	981.5338	982.4488
982.7716	983.8687	984.9936
985.3161	986.2453	986.9111
987.1053	987.3861	989.0623
991.0214	993.1147	995.8329
1003.6690	1021.6494	1023.1671
1023.4084	1024.0105	1026.0307
1026.5427	1028.9748	1030.0499
1030.1987	1044.8868	1046.9738
1058.8668	1071.8536	1072.3326
1073.4995	1074.2352	1074.8012
1075.7075	1077.7471	1079.9309
1091.0876	1097.0719	1097.9876
1128.1720	1132.1826	1134.0029
1134.6757	1135.5843	1136.2502
1136.5224	1136.5824	1137.8233
1137.9502	1138.9074	1140.5237
1146.6502	1147.1819	1154.0266
1154.7037	1156.7001	1156.8654
1157.4342	1159.6825	1160.5231
1163.4920	1166.3552	1176.6255
1187.8383	1194.2750	1196.6546
1238.5214	1241.0460	1258.5265
1269.8028	1272.6522	1273.0606
1275.5555	1280.1255	1280.6968
1283.3404	1284.4176	1289.3168
1289.5545	1303.0176	1316.2071
1327.1579	1336.5176	1341.7288
1345.8150	1346.3145	1360.8831
1361.2922	1368.3638	1368.9946
1371.3143	1372.3024	1375.4643
1375.9405	1395.8500	1420.3091
1426.5822	1428.8508	1430.3834
1430.7562	1430.9074	1432.9248
1434.0184	1440.4785	1440.9575
1457.1599	1467.4138	1470.4514
1471.9108	1472.0390	1473.6820
1473.8759	1474.3204	1478.8050
1480.6563	1504.7415	1554.6292
1570.8578	1572.3455	1573.3144
1574.6491	1575.4076	1580.6562
1583.9094	1590.7271	1592.1183
1593.3139	1594.1552	1599.4763
1601.1918	1603.8456	1604.7459
1607.5131	1607.8422	1608.6380
1610.6351	1611.4742	1614.4378
1624.1546	1690.5988	3095.0607
3095.7531	3096.3074	3096.5010
3096.7946	3097.0802	3097.6356
3098.9138	3099.5449	3099.7329
3101.0052	3102.8520	3103.6337
3104.4372	3104.5954	3105.5452
3105.5492	3105.8707	3106.2268
3107.9327	3109.6936	3112.4040
3112.5435	3113.1923	3113.6618
3114.0830	3115.2680	3116.1404
3116.9635	3117.5756	3117.8083
3117.8801	3119.7620	3120.5150
3122.4565	3122.5829	3123.5264
3123.8149	3124.3649	3124.9011
3125.5587	3125.8417	3126.1973
3127.1991	3127.2687	3130.0843
3130.7162	3130.8558	3131.2866
3132.1572	3138.8553	3148.3564

$\mathbf{6}_{Q}$

11.8397	16.4028	20.4524
26.5339	28.2991	31.0861
37.3762	41.9151	46.3200
47.1424	49.2966	54.2746

55.0613	57.7388	62.8531
64.3752	70.2287	70.7926
73.1304	77.3214	78.4130
82.0194	84.4817	89.2520
91.0279	95.0846	97.2536
101.2474	103.7346	107.9495
112.0427	115.1794	118.9000
121.4609	127.6859	136.2692
146.1349	148.8867	176.2151
183.6553	198.2937	202.0934
207.5204	213.6848	221.4846
225.8219	230.6075	234.9170
238.7279	247.2185	253.0533
258.4376	262.0901	272.1973
278.3148	282.3818	288.7481
293.7429	294.3017	298.4449
310.9918	319.2153	322.4891
336.0680	342.2461	351.9430
352.1529	371.6829	378.6685
386.5576	399.1405	401.1905
401.8485	404.8074	406.4505
407.8861	409.6279	410.6946
415.8969	416.2301	420.1211
421.5457	426.5808	459.4121
466.8263	472.4857	479.8704
486.8760	493.7119	508.3793
510.4567	514.4971	518.0260
530.7488	541.0238	546.4411
552.3285	563.2361	578.7358
587.4461	590.6064	611.5133
611.9747	612.5715	612.7332
612.9514	613.7886	614.3241
615.2492	616.1645	625.5827
627.4833	629.7916	631.1278
633.1560	633.7840	635.9151
663.4792	667.4493	687.2340
689.1734	694.0590	695.9993
698.5548	701.0430	701.8106
702.5820	708.8747	710.7123
711.3431	724.7189	730.9448
736.6134	746.2199	750.0157
750.5466	752.0586	752.8561
756.0523	760.6602	764.2721
765.0228	766.6514	778.2350
798.6194	812.9210	815.6403
818.8494	827.3468	828.3724
829.3453	833.8129	835.6540
836.6337	839.3110	849.1649
862.7352	868.6673	884.2258
893.9764	894.3299	901.4515
901.8107	904.4554	908.1739
910.6688	917.0373	922.2696
925.2809	925.3855	928.9117
931.1100	933.3974	944.7943
945.1338	954.8923	956.4182
956.9944	961.1590	963.5227
964.0908	964.9400	969.6209
973.2433	975.6668	976.4555
977.0591	977.9513	978.8902
980.9723	981.2632	982.4754
983.5526	983.8570	985.7884
986.0046	986.6391	987.0278
987.5946	988.2461	989.9183
992.3285	1000.4810	1003.4498
1016.5950	1022.6952	1023.2186
1024.4759	1025.2385	1025.6686
1026.2254	1027.0371	1028.2944
1029.7113	1042.6258	1048.2993
1050.6414	1069.3062	1073.6476
1073.7800	1074.7580	1075.7524
1077.1122	1078.6130	1082.0752
1088.0769	1095.1369	1097.5178
1127.1414	1131.3358	1133.7891
1134.3723	1134.9080	1135.0909
1136.3053	1136.4910	1136.7671
1136.8706	1137.8198	1138.1643
1145.7878	1148.1234	1152.9108
1153.1828	1156.0294	1156.4091
1159.3374	1159.7651	1162.2151
1163.1825	1167.4192	1177.2762
1181.3074	1194.5455	1196.8489
1237.2120	1241.1626	1260.3650
1266.3351	1269.3828	1270.3373
1275.2858	1278.6560	1280.4084
1283.3763	1286.0337	1286.6876
1296.4253	1303.5404	1306.6162
1326.0530	1331.2255	1340.2142
1342.5111	1345.8962	1360.5234
1361.4837	1366.0050	1369.5940
1370.6592	1373.5215	1374.5640
1376.8695	1392.6066	1420.7699
1425.8923	1427.3586	1428.9276
1430.5904	1431.4882	1434.0484
1434.4408	1439.5012	1441.5510
1455.9006	1468.8884	1470.4574
1472.0127	1473.6693	1473.7557
1474.9693	1476.2322	1479.1438
1481.6015	1505.0670	1555.0660
1571.1726	1572.8913	1574.8108
1575.6561	1577.5228	1581.3416
1586.8041	1592.1317	1592.4609
1593.3082	1596.1434	1599.7396
1600.7966	1605.2443	1605.8757
1606.8098	1607.7909	1608.8377
1610.7815	1613.3153	1615.5436
1642.8692	1739.8098	3086.0132
3093.9214	3094.6627	3095.0255
3095.1044	3096.0836	3096.3088
3096.4301	3096.7215	3097.0457
3098.2872	3101.7201	3102.1950
3102.7926	3104.5304	3106.0646
3106.1236	3107.2396	3108.5970
3110.1692	3110.1940	3110.3181
3110.7802	3111.0331	3113.4469
3113.6370	3114.3879	3117.3554
3117.4067	3117.5245	3117.7195
3118.0381	3119.4958	3121.3590
3121.8238	3122.5788	3122.7110
3126.2356	3126.8445	3126.9834
3127.4629	3128.4646	3128.6052
3129.4140	3129.9220	3130.7807
3132.4771	3135.5043	3136.9443
3137.9984	3138.1600	3163.8400
